# Pentacyclic Cytochalasins and Their Derivatives from the Endophytic Fungus *Phomopsis* sp. xz-18

**DOI:** 10.3390/molecules26216505

**Published:** 2021-10-28

**Authors:** Guichon Huang, Weiwen Lin, Hanpeng Li, Qian Tang, Zhiyu Hu, Huiying Huang, Xianming Deng, Qingyan Xu

**Affiliations:** 1 State Key Laboratory of Cellular Stress Biology, School of Life Sciences, Xiamen University, Xiamen 361102, China; ghhuang1108@foxmail.com (G.H.); lin_wwen@163.com (W.L.); lihanpeng@stu.xmu.edu.cn (H.L.); tangqian2383@163.com (Q.T.); huzhiyu@xmu.edu.cn (Z.H.); hyinghuang@xmu.edu.cn (H.H.); 2 State-Province Joint Engineering Laboratory of Targeted Drugs from Natural Products, Xiamen University, Xiamen 361102, China

**Keywords:** cytochalasins, pentacyclic system, octant rule, antibacterial activity

## Abstract

Eight new cytochalasins **1**–**8** and ten known analogs **9**–**18** were isolated from the endophytic fungus Phomopsis sp. xz-18. The planar structures of the cytochalasins were determined by HR-ESI-MS and NMR analysis. Compounds **1**, **2**, **9** and **10** were 5/6/6/7/5-fused pentacyclic cytochalasins; compounds **3** and **4** had conjugated diene structures in the macrocycle; and compound **6** had a β,γ-unsaturated ketone. The absolute configuration of 6 was confirmed for the first time by the octant rule. The acid-free purification process proved that the pentacyclic system was a natural biosynthetic product and not an acid-mediated intramolecular cyclized artifact. The new compounds did not exhibit activities against human cancer cell lines in cytotoxicity bioassays or antipathogenic fungal activity, but compounds **1**, **3** and **4** showed moderate antibacterial activity in disk diffusion assays.

## 1. Introduction

Cytochalasans are fungal polyketide-amino acid hybrid metabolites with structural and bioactive diversities that have attracted continuous interest from chemists and biologists since the structure of the first cytochalasan was elucidated in 1966 [1]. These are microfilament-directed agents commonly used in basic research to understand cytoskeletal mechanisms [1]. Cytochalasins are a type of cytochalasans with phenylalanine incorporated into the polyketide backbone and a benzyl group-substituted hydrogenated isoindolone framework [2], to which typically an 11- or 13-membered carbocycle [3,4], a 14-membered lactone ring [5], or an open 8-carbon chain system [6] is fused. Recently, phomopchalsin A-B [7] and cytochalasin J3 [8], containing an 11-membered dicycle system and unprecedented pentacyclic structures, were discovered in cultures of the endosymbiotic fungus *Phomopsis* sp., which greatly increased the chemical diversity of known cytochalasins. Cytochalasins exert a wide range of biological effects, such as cytotoxic, anticancer, antimicrobial, and antiparasitic activities. For example, cytochalasin B was found to bind to the glucose carrier in human erythrocytes through hydrogen bonds and inhibit glucose transport [9]. Phomopsichalasin G, from mangrove fungal endophytes, exhibited inhibitory activities against several cancer cell lines with 50% inhibitory concentration (IC50) values at the micromolar level [10].

**Figure 1:**
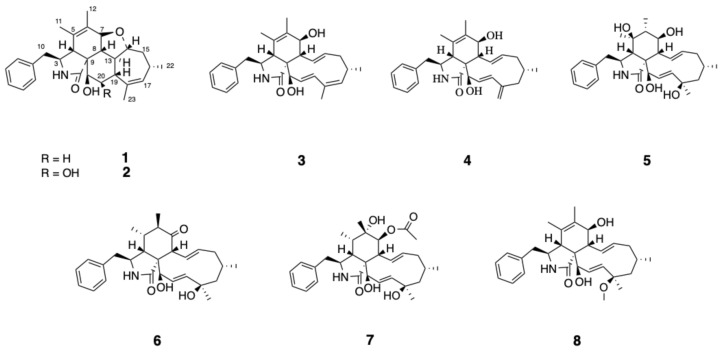
Structures of new compounds **1**–**8**.

Botanic endophytic fungi, including the genera *Penicillium*, *Phomopsis*, *Asepergillus*, etc., are the main cytochalasin-producing organisms. In our ongoing effort to discover microbial natural products from endogenous habitats, *Phomopsis* sp. strain xz-18, from the stem of *Camptotheca acuminatem* collected in Jiangshi Natural Reserve (Fujian Province, China), showed noticeable anticancer activities during primary screening of the crude extract. Extensive purification gave rise to four 5/6/6/7/5-fused pentacyclic cytochalasins **1**, **2**, **9**, **10** and fourteen related 11-membered macrocyclic compounds **3**–**8**, **11**–**18**. Compounds **1**–**8** (Figure 1) were identified for the first time.

## 2. Results and Experiments

### 2.1. Structure Elucidation

The extract of a 20 day scaled up potato dextrose agar (PDA) solid medium cultivation of *Phomopsis* sp. strain xz-18 in a mixture of ethyl acetate (EA)/MeOH/acetic acid (HOAc) (80/15/5, volume ratio) was concentrated in vacuo. Then, the crude extract was subjected to reversed-phase column chromatography, and the 63–68% MeOH eluate fraction was subjected to Sephadex LH-20 purification in MeOH and then in acetone, affording compounds **1**, **2**, **9** and **10**. The remaining compounds were purified with similar column chromatography steps.

Phomopchalasin C_1_ (**1**) was isolated as a colorless powder. The molecular formula was deduced as C_28_H_35_NO_3_ by high resolution-election spray-ionization-mass spectrometry (HR-ESI-MS) (*m/z* 434.2682, [M + H]^+^; calculated for C_28_H_36_NO_3_: 434.2690), which indicated 12 degrees of unsaturation. The IR spectrum exhibited bands due to NH and/or OH (3266 cm^−1^) and amide groups (1697 cm^−1^). The *1H*-NMR spectrum (Table 1) showed the presence of four methyl, three methylene, and fifteen methine (including one olefinic and five phenyl methine) moieties at δ_H_ 7.21–7.36.

The ^13^C-NMR spectrum (Table 1) showed a total of 26 carbons. Taking into account two methines (δ_C_ 128.8 and δ_C_ 129.4) on the single-substituted phenyl, the total number of carbons conformed to the predicted molecular formula. The HSQC spectrum revealed five quaternary carbons, including one carbonyl carbon (δ_C_ 176.0) and three olefinic carbons (δ_C_ 126.9, 135.0, 137.5). With regard to the 12 degrees of unsaturation, deducting four for a benzene ring, one for a carbonyl group, and two for two olefinic bonds, the remaining five degrees of unsaturation indicated a five ring system.

**Figure 2:**
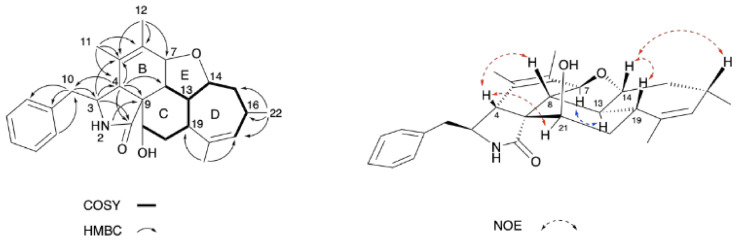
2D correlations for compound **1**.

Extensive analysis of the HMBC and 1H-1H COSY spectra led to the elucidation of the planar structure of **1**. The HMBC correlations (Figure 2) H-2′ to C-10; H-10 to C-3, C-1′, and C-2′; H-3 to C-1, C-4, and C-5; H-4 to C-1, C-5, C-8, C-9, and C-10; H3-11 to C-4, C-5, and C-6; and H3-12 to C-5, C-6, and C-7; as well as H-2 to C-4 and C-9; established the benzyl tetrahydro-isoindolone bicyclic lactam. This characteristic substructure was similar to that of rings A and B of cytochalasins. The other part of this compound was deduced mainly based on ^1^H-^1^H COSY correlations (Figure 2). The cross peaks of H-8/H-13/H-14/H-15/H-16/H-17, H-19/H-20/H-21, and H-13/H-19 established the 11-membered bicyclic system of rings C and D. The last ring E was formed via an oxygen bridge between two hydroxyl groups at C-7 and C-14 mainly on the basis of the downfield chemical shifts of C-7 (δ_C_ 77.2) and C-14 (δ_C_ 87.9); thus, the planar structure of **1** was established. To the best of our knowledge, **1** is the third 5/6/6/7/5-fused pentacyclic backbone cytochalasin ever discovered, with a 5,6-ene structure at ring B.

The relative configuration of **1** was established by NOE analysis (Figure 2) and its coupling constants. The large coupling constants of *J ^7/8^* (10.7 Hz), *J ^8/13^* (9.8 Hz) and *J^13/14^* (9.7 Hz) implied that H-7 and H-8 were antiplanar, H-7 and H-13 were co-facial, and H-8 and H-14 were also co-facial, respectively. These NOE correlations could unambiguously deduce the relative configurations of all chiral atoms. Considering that the absolute configuration of cytohexane and isoindole moieties (ring A and B) are the same in all cytochalasans isolated thus far [6], we assumed a 3*S*,4*R*,7*S*,8*R*,9*R*,13*S*,14*R*,16*S*,19*R*, 21*R* stereochemistry, which is that of known compound **10** [7]. The similar circular dichroism (CD) spectra of **1** and **10** (Figure S78), as well as the same biogenic origin from the endogenous fungus *Phomopsis* sp., support this hypothesis.

The molecular formula of phomopchalasin C_2_ (**2**) was assigned as C_28_H_35_NO_4_ based on its positive HR-ESI-MS ion at m/z 450.2629 [M + H]^+^ (calcd. 450.2639), containing one more oxygen atom than **1**. Comparing the NMR data (Table 1) of **1** and **2** revealed that the major structural difference was at C-20. An oxygen-containing methine (δ_H_ 4.57, δ_C_ 71.8) took the place of the methylene (δ_H_ 2.07, 2.38, δ_C_ 34.4) of **1**. After acetylation with acetic anhydride in pyridine (to form acetylated compound **2**), δ_H_-20 shifted from 4.57 ppm to 4.80 ppm (Figure S69), indicating that OH-20 was free in compound **2**. This compound was another 5/6/6/7/5-fused pentacyclic cytochalasin. The relative R* configuration of C-20 was established based on the NOE correlation of H-20 to H_3_-23, indicating an α-orientation. Comparing the CD spectrum of **2** (Figure S79) with that of compound 1 established its absolute configuration as 3*S*, 4*R*, 7*S*, 8*R*, 9*R*, 13*R*, 14*R*, 16*S*, 19*R*, 20*R*, 21*S*.

**Figure 3:**
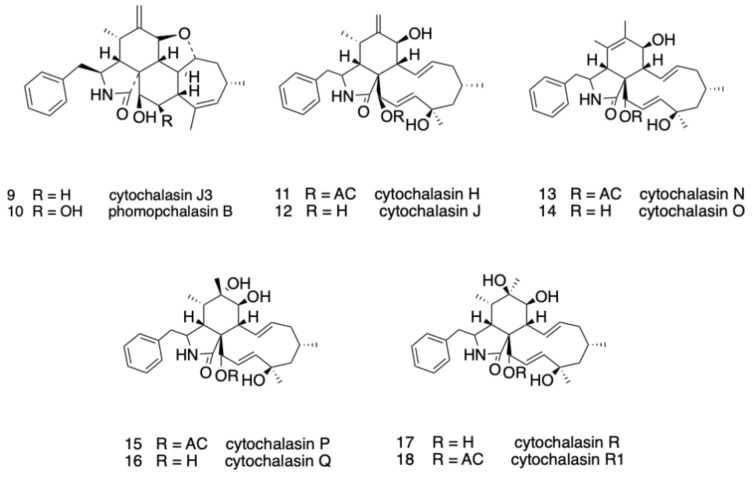
Structures of compounds **9**–**18**.

Comprehensive interpretation of 1D and 2D NMR data (Tables S3–S8) suggested that phomopchalasin C_3_ (**3**)-phomopchalasin C_8_ (**8**), as well as ten known compounds **9**–**18** (Figure 3), belonged to the 10-phenyl-[11] cytochalasans class.

Phomopchalasin C_3_ (**3**) and phomopchalasin C_4_ (**4**) had the same molecular formula of C_28_H_35_NO_3_ based on their positive HR-ESI-MS ions at *m/z* 434.2689 and 434.2686, respectively (calcd. [M + H]^+^ C_28_H_36_NO_3_: 434.2690). Spectral analysis revealed that a structural difference existed at C-17 and C-23. while **3** had a 17,18-ene bond, **4** had a 18,23-ene bond. With a 19,20-olefinic bond, a conjugated diene structure was formed in both compounds.

The conjugated diene structure gives rise to a strong Cotton effect (CE) at approximately 250–260 nm in the CD spectrum. For **3**, the CE was negative, and for **4**, it was positive (Figure S86). Correlation analysis between the CE and spiral chirality of the conjugated diene is shown in Figure S86. Considering the results of isotope labeling experiments and biogenetic studies [2] of all cytochalasans isolated thus far showing the configurations of C-4 and C-9, two identical bridgehead carbons of ring A/ring B, the absolute configuration of **3** and **4** was determined to be 3*S*, 4*R*, 7*S*, 8*R*, 9*R*, 16*S*, 21*R*.

Phomopchalasin C_5_ (**5**) had a molecular formula of C28H39NO5 deduced by HR-ESI-MS (*m/z* 470.2897, [M + H]^+^; calcd. for C_28_H_40_NO_5_: 470.2901). Analysis of the NMR spectrum (Table S5) revealed that **5** had an identical planar structure to cytochalasin S [3]. The NOE correlation (Table S5) from H-3 and H-7 to H3-11 and H3-12, respectively, and from H-6 to H-8 indicated that CH_3_-11 and CH_3_-12 were α-oriented in **5**, different from cytochalasin S, in which CH_3_-11 and CH_3_-12 are β-oriented; that is, cytochalasin S and **5** were epimers at C-5 and C-6. Cytochalasins with OH-7 and OH-6 are common, but compounds with OH-5 were formerly reported as artifacts when cytochalasin with a 5,6-epoxide underwent acidic hydrolysis with sulfuric acid in vitro [11]. Compound **5** and cytochalasin S are natural metabolites with a OH-5 substituent, which is very rare to date. The absolute configuration of **5** was proposed to be 3*S*, 4*S*, 5*R*, 6*R*, 7*S*, 8*R*, 9*S*, 16*S*, 18*R*, 21*R* based on the above-mentioned reasons.

Phomopchalasin C_6_ (**6**) had a molecular formula of C_28_H_37_NO_4_ deduced by HR-ESI-MS (*m/z* 452.2799, [M + H]^+^; calcd. for C_28_H_38_NO_4_: 452.2795), 18 mass units less than **5**, and their NMR data differed at C-5, C-6 and C-7. The NOE correlation (Table S6) from H-3 to H_3_-11 and H-6 and from H-4 to H_3_-12 suggested that CH_3_-11 and CH_3_-12 were α- and β-oriented, respectively. The δ_C_ 215.4 ppm and HMBC correlation (Table S6) of H-12 to C-5, C-6 and C-7 determined the presence of a 7-ketone functional group. Based on this information and that outlined previously, the absolute configuration of **6** was determined to be 3*S*, 4*R*, 5*S*, 6*R*, 8*R*, 9*R*, 16*S*, 18*R*, 21*R*.

**Figure 4:**
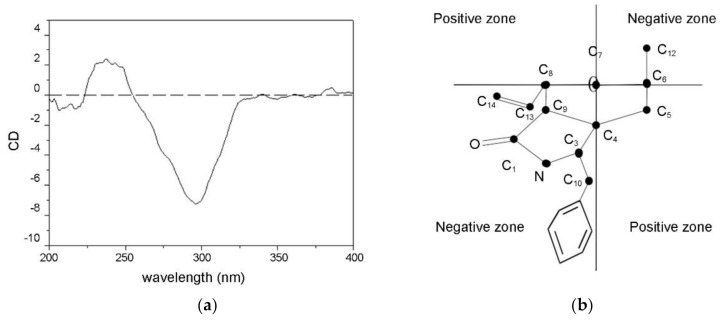
CD spectrum (**a**) and backfourregion of octant rule (**b**) for compound **6**.

The β,γ-unsaturated ketone formed by C-7/C-13/C-14 in compound **6** gave a strong, negative CE at approximately 300 nm (Figure 4), from which the absolute configuration of C-8 could be illustrated with the octant rule [12]: from the viewpoint of the C=O bond illustrated in Figure S87, a large proportion of this compound, including C-13, C-14 and ring A, are positioned in the negative zone of the back-four-regions of the ketone group (Figure 4), which indicates that the absolute configuration of C-8 is R. This result was consistent with the aforementioned stereochemistry. The octant rule is a reliable method to determine the absolute configuration of compounds but is not commonly used on complicated natural compounds. This was the first example of determining a cytochalasin chiral carbon’s absolute configuration with the octant rule.

Based on HR-ESI-MS analysis and NMR data (Tables S7 and S8), phomopchalasin C_7_ (**7**) and phomopchalasin C_8_ (**8**) were confirmed as 7-O-acetyl cytochalasin P [3] and 18-O-methyl cytochalasin O [3], respectively. Ten cytochalasin derivatives, cytochalasin J_3_ (**9**) [8], phomopchalasin B (**10**) [7], cytochalasin H (**11**), cytochalasin J (**12**), cytochalasin N (**13**), cytochalasin O (**14**), cytochalasin P (**15**), cytochalasin Q (**16**), and cytochalasin R (**17**) [3] and R1 (**18**) [13] (Figure 3), were purified from the same strain, and their structures were confirmed by comparison of NMR spectra (Figures S49–S68) and data from the literature.

**Figure 5:**
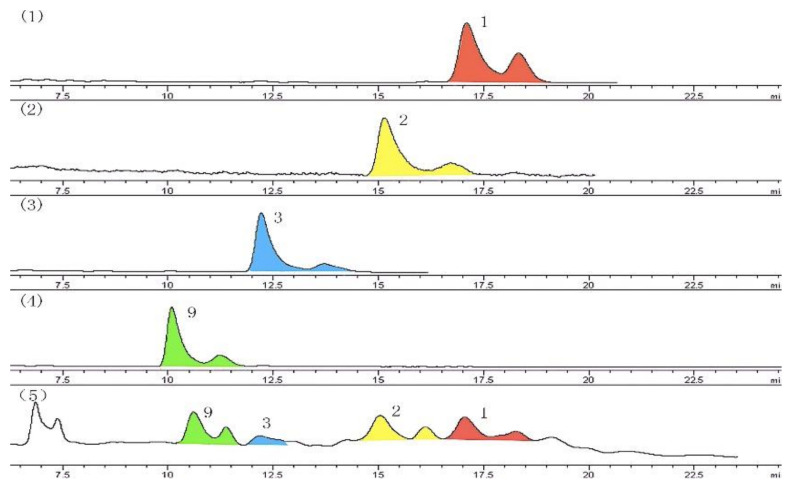
HPLC-DAD (210 nm) chromatograms. (1) Phomopchalasin C_1_ (**1**), (2) phomopchalasin C_2_ (**2**), (3) phomopchalasin C_3_ (**3**), (4) cytochalasin J3 (**9**), (5) the fraction of MeOH/H_2_O (80/20, *v/v*) eluted from the crude extract.

Atipathogenic fungal compounds **9**, **11** and **12** were previously reported to have been extracted from *Phomopsis* sp. (CMB-M0042F), a marine-derived fungus [8]. It has been reported that **9** is considered an acid-mediated conversion artifact produced from **12** during purification by repeated silica gel chromatography. As evidence, **12** was transformed to **9** under conditions invoving exposure to trifluoroacetic acid (TFA)/MeOH (1/100, *v/v*) at room temperature [8]. Similarly, under acidic conditions, where HOAc was used as the extractant during our purification process, **1** and **2** were also observed as artifacts of the possible precursor cytochalasin O (**14**). To confirm this hypothesis, a 21 d scaled-up PDA solid medium culture of *Phomopsis* sp. xz-18 was extracted with EtOAc/MeOH (50/50, *v/v*). The crude extract was then subjected to reversed-phase silica RP-18 purification with MeOH/H_2_O. The fraction of MeOH/H_2_O (80/20, *v/v*) was analyzed by HPLC-diode array detection (DAD). As shown in Figure 5, the chromatograms revealed that **1**, **2**, **3**, and **9** were all present in the crude extract, and purified compounds were used as standards for confirmation.

Without using acidic conditions, no normal-phase chromatography or potentially acidic CHCl_3_ or HOAc was employed during the whole process. These data suggested that **1** and **2** were not acid-mediated artifacts but rather naturally produced by the endophytic fungus *Phomopsis* sp. xz-18.

### 2.2. Bioactivity

Compounds **1**–**10** were evaluated in vitro for cytotoxic activity, antipathogenic fungal activity and antibacterial activity. All compounds were inactive against a panel of cancer cells, including A375 (human melanoma cells), A549 (human lung carcinoma cells), BGC-823 (human pancreatic cancer cells), HCT116 (human colon cancer cells), HePG-2 (human hepatoma carcinoma cells), HL-60 (human acute promyelocytic leukemia cells), Jeko-1(human mantle cell lymphoma cells), Kyse-450 (human esophageal cancer cell), MCF-7 (human breast cancer cells) and U-2 OS (human osteosarcoma cell), and were inactive against pathogenic fungi *Beauveria vuillemin*, *Curvularia lunata* and *Setosphaeria turcica*. However, **1**, **3**, and **4** showed moderate antibacterial activity against *Bacillus subtilis* CMCC63501, *Bacillus pumilus* CMCC63202, *Candida albicans* AS2.538, and *Asperillus niger* ACCC3005 with inhibition zone diameters between 7 and 10 mm in a disk diffusion assay (Table 2).

### 2.3. Proposed Biosynthetic Pathway of ***1**–**5*** and ***8***

**Scheme 1:**
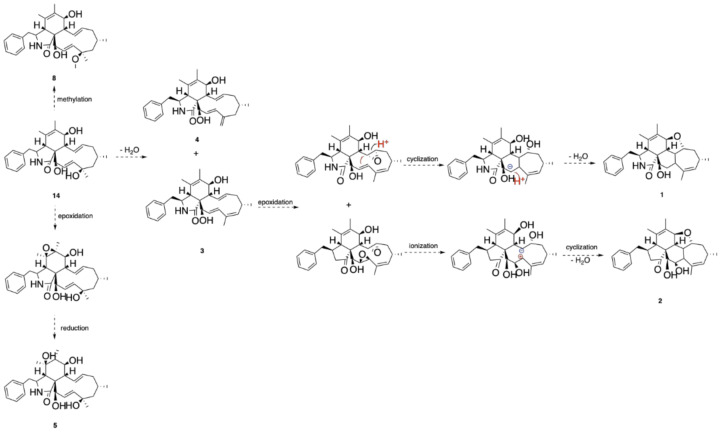
Proposed biogenesis of **1**–**5** and **8** from **14**.

In this study, eighteen cytochalasins were isolated from the endophytic fungus *Phomopsis* sp. strain xz-18. According to the features of ring B, the eighteen compounds could be divided into three groups: **1**–**4**, **8**, and **13**–**14** which had 5,6-double bonds; **9**–**12** which had 6,12-double bonds; and the remaining compounds had ring B hydrogenation. On the basis of these structural features, we can propose a biogenetic pathway (Scheme 1). It is well known that epoxidation of double bonds is a common biosynthetic reaction leading to the formation of alcohols, which could also be the initiating reaction resulting in cyclized metabolites in cytochalasins [14]. Compounds **1**–**5** and **8** might share common biosynthetic precursor compound **14,** which originates from a polyketide-amino acid hybrid. When the 5,6-double bond of **14** undergoes epoxidation, reduction, and then hydroxylation at C-5, compound **5** is obtained. When dehydration occurs at C-17/C-18 or C-18/C-23, **3** or **4**, respectively, are obtained. Compound **3**, having a 13,14-double bond, undergoes a series of reactions, including epoxidation, cyclization, and dehydration, leading to the production of **1**. Under the condition that both the 13,14- and 19,20-double bonds undergo epoxidation, compound **2** could be obtained as the final product (Scheme 1). Although this hypothesis needs to be confirmed by isotope labeling methods in situ or by enzyme catalytic reactions in vitro, the discovery of flavichalasine compounds supports the proposition that many structurally similar metabolites isolated from one fungal strain have a great possibility of originating from a common precursor [15].

## 3. Conclusions

In summary, since the first cytochalasin was isolated from the mycelial fungus *Helminthosporium dematioideum* in 1967 [16], over 100 cytochalasins have been isolated, featuring diverse characteristics and including 11- to 14-membered macrocycles or ring-opened derivatives, unprecedented 5/6/5/8-fused tetracyclic skeletons and 5/6/6/7/5-fused pentacyclic skeletons. In this article, we reported eight new and ten known cytochalasins, including all four 5/6/6/7/5-fused pentacyclic cytochalasins **1**, **2**, **9**, **10** identified to date, which provides new chemical diversity to the cytochalasin class. We have proven that the pentacyclic structure of cytochalasins is not an acid-mediated intramolecular cyclized artifact but rather they are natural metabolites produced by microorganisms. We verified the absolute configuration of cytochalasins by the octant rule for the first time, and the results were consistent with previous results from X-ray diffraction (XRD), electron capture detection (ECD) comparison, or chemical synthesis. Therefore, the octant rule has been extended to determine the absolute configuration of relatively complicated compounds, such as cytochalasans, compared with its previous application to cyclopentanone, cyclohexanone, and α, β- and β, γ-unsaturated ketone compounds. Additionally, the proposed biosynthetic pathway shed light on the great potential of producing diverse metabolites from this endophytic fungal strain, particularly via epoxidation at 13,14- and/or 19,20-double bonds of **4** or dehydration from compound **5**, implying that other 5/6/6/7/5-fused structures or other novel structures may be found in the near future.

## 4. Materials and Methods

### 4.1. General Experimental Procedures

Optical rotations were measured on a model 314 polarimeter (Perkin Elmer, Waltham, MA, USA). Fourier transform infrared (FT-IR) spectra were obtained using a Nicolet iS5 FT-IR spectrometer from Thermo Scientific (Waltham, MA, USA) equipped with a mid-IR ceramic emitter and a deuterated-glyceride detector at a 4 cm^−1^ resolution for 20 scans on KBr pellets. CD spectra were recorded on a J-815 spectropolarimeter (Jasco, Tokyo, Japan). NMR spectra were measured on an Avance-III 600 MHz spectrometer (Bruker, Karlsruhe, Germany) equipped with a Prodigy cryoProbe. The spectra were acquired in CDCl3, and the chemical shifts were reported in ppm referring to CHCl3 (δ_H_ 7.26 for protons and δ_C_ 77.0 for carbons). ESI-MS spectra were measured on a model 3100 mass spectrometer (Waters, Milford, MA, USA) coupled with a Waters 2545 HPLC system. HR-ESI-MS spectra were obtained at the National Center for Organic Mass Spectrometry in Shanghai on a Thermo Fisher Scientific LTQ FT Ultra spectrometer in DART-positive ionization mode. Analytical thin-layer chromatography (TLC) was performed on precoated silica gel plates (0.2 mm) obtained from Qingdao Ocean Chemical Plant (Qingdao, China) with detection provided by UV light (254 nm). MPLC purification was performed on a Sepacore Easy Extract purification system (Büchi, Uster, Switzerland) using Merck Silica gel 60 RP-18, whereas HPLC purifications were carried out on a 1200 series chromatograph (Agilent, Santa Clara, CA, USA) equipped with a DAD detector. Size-exclusion chromatography was performed on a Sephadex LH-20 column.

### 4.2. Experimental Biological Material

All cell lines were obtained from the Shanghai Institute of Biochemistry and Cell Biology (Shanghai, China). Medium, fetal bovine serum (FBS), and complementary reagents were purchased from HyClone (Boston, MA, USA) or Gibco (Waltham, MA, USA). 3-(4,5-Dimethyl-2-thiazolyl)-2,5-diphenyl-2*H*-tetrazolium bromide (MTT) was purchased from Sigma Chemical Co. (St. Louis, MO, USA). HL-60 cells and Jeko-1 cells were grown in Gibco RPMI 160 medium with 10% qualified FBS. HepG-2 cells, A549 cells, BGC823 cells, HCT116 cells, MCF-7 cells, U-2 OS cells, A375 cells, and Kyse-450 cells were grown in Dulbecco’s modified Eagle’s medium (DMEM) supplemented with 10% qualified FBS. Cell cultures were maintained in a humidified incubator at 37 °C with 5% CO_2_. Plant pathogenic fungal strains *B. vuillemin*, *C. lunata* and *S. turcica* were purchased from the Institute of Plant Protection, Fujian Academy of Agricultural Sciences (Fuzhou, China). The strains were grown on PDA solid medium at 28 °C for 48 h to activate the frozen strains. *B. subtilis* CMCC63501 and *B. pumilus* CMCC63202 were grown in LB medium, *C. albicans* AS2.538 and *A. niger* ACCC3005 were grown on PDA medium, and all four strains were obtained from collections of our laboratory.

### 4.3. Fungal Production and Cultivation

The producing organism *Phomopsis* sp. xz-18 was isolated from the stems of *C. acuminate* collected from the Jiangshi Natural Reserve (Fujian Province, China), in June 2004, and the isolation method has been previously described [16]. The ITS1-5.8 S-ITS4 region of the fungus was completely sequenced (GenBank Accession No. DQ14534.1). Compared with the GenBank database using the BLAST application, the similarity of the isolated fungus was 100% with *Phomopsis* sp. Frozen stock cultures were maintained in 10% glycerol at −80 °C in our laboratory. To scale up the fermentation to 40 L of solid culture, 10 mycelial discs of fungal strain grown on PDA (200 g of peeled potato was minced and boiled for 30 min, the residue was discarded, and the remaining mixture was combined with 20 g of dextrose, 20 g of agar, and distilled H_2_O to a total volume of 1 L) at 28 °C for 7 d were used to inoculate 50 mL of PD medium in a 250 mL flask. After incubation at 220 rpm and 28 °C for 5 d, a 0.1 mL aliquot of this culture was used to inoculate one disc (d = 9 cm) of PDA solid medium. The discs were incubated upside down at 28 °C for 20 d.

### 4.4. Extraction and Purification

The solid culture medium was cut into pieces approximately 0.5 × 0.5 × 0.5 cm^3^ in size and soaked in EA/MeOH/HOAc (80/15/5, volume ratio) five times. After filtration, the extracts were combined and concentrated in vacuo at 40 °C to obtain a residue. After dissolving the residue in methanol, it was degreased with petroleum ether three times. After concentration in vacuo again, the final extract (11.83 g) was loaded on a silica gel 60 RP-18 column (170 g, 8 × 45 cm^2^) and eluted with a MeOH-H_2_O gradient (MeOH 30–100% in 2.5 h) to yield 17 fractions.

Fraction 7 (1180 mg) was purified by a Sephadex LH-20 column (140 g, 2.5 × 150 cm^2^) in MeOH (15 s/drip, 1 tube/h), and two subfractions were obtained. Subfraction 1 (109 mg) was further purified with a Sephadex LH-20 column (140 g, 2.5 × 150 cm^2^) in acetone (15 s/drip, 1 tube/h) and then subjected to preparative TLC (CHCl_3_/MeOH = 10/1, *v*/*v*) to yield 8.5 mg of **6** (*R*_f_ = 0.3) and 19 mg of **7** (*R*_f_ = 0.5).

Fraction 8 (1570 mg) was purified by a silica gel 60 RP-18 column (80 g, 3.5 × 24 cm^2^) with a MeOH-H_2_O gradient (MeOH 34–74% in 1.5 h) to obtain four subfractions. Subfraction 3 (354 mg) was further purified with a Sephadex LH-20 column (140 g, 2.5 × 150 cm) in MeOH and preparative HPLC (Agilent 1200, 5 μm, 4.6 × 250 mm^2^, 3 mL/min, UV detection at 210 nm and 254 nm) with an eluent of MeOH/H_2_O (65:35, 1.5 mL/tube) to yield **8** (2 mg) and **10** (2 mg). Subfraction 4 (302 mg) was subjected to a Sephadex LH-20 column (140 g) in acetone (15 s/drip, 1 tube/h), and preparative HPLC (Agilent 1200, 5 μm, 4.6 × 250 mm^2^, 3 mL/min, UV detection at 210 nm) with MeOH/H_2_O (70:30, 1.5 mL/tube) to yield 10 mg of **1**, 14 mg of **2**, 21 mg of **3**, 2 mg of **4**, and 8 mg of **9**, respectively.

Fraction 10 (1360 mg) was purified by a Sephadex LH-20 column (140 g, 2.5 × 150 cm^2^) in MeOH, and then the same gel filtration chromatography was performed in acetone to obtain 2.0 mg of compound **5**.

Acetylation of phomopchalasin C_2_ was conducted with 1 mg of phomopchalasin C_2_, 0.1 mL of pyridine, and 0.1 mL of acetic anhydride that were mixed together in a sealed 5 mL sample bottle. After 24 h in a dryer at room temperature, the mixture in the sample bottle was mixed with 0.5 mL of CDCl_3_, and ^1^H-NMR correlations were determined.

### 4.5. MTT Cytotoxicity Assay

A total of 6 × 104~1 × 105 cells/well for all cell lines were cultured for 24 h before compounds were added at 1/100 dilutions in triplicate following a previously described method [16] (2 μL of 0.33 mM compound solution in DMSO into 198 μL fresh medium per well) for a 72 h assay. Cell-free wells and vehicle control wells were included in each plate. Cell viability was determined using MTT (3-(4,5-dimethylthiazol-2-yl)-2,5-diphenyl tetrazolium bromide). At the times indicated below, stock MTT solution (5 mg/mL in PBS) was added (10 μL per 100 μL medium) to all wells of an assay, and plates were incubated at 37 °C for 4 h. Acid-isopropanol (100 μL of 0.04 N HCl in isopropanol) was added to all wells and mixed thoroughly to dissolve the dark blue crystals. After a few minutes at room temperature to ensure that all crystals were dissolved, the absorbance values were read at 570 nm wavelength with a spectrophotometer (Varioskan Flash, Thermo, Waltham, MA, USA). The cell viability was calculated as cell survival = (ODcompd. − ODblank)/(ODcontrol − ODblank) × 100%. DMSO was used as control. IC50 was calculated using software GraphPad Prism 5 (GraphPad Software, San Diego, CA, USA).

### 4.6. Disk Difusion Methodology

Three plant pathogenic fungal strains (*B. vuillemin*, *C. lunata* and *S. turcica*) and four indicator organisms (*B. subtilis* CMCC63501, *B.*, *C. albicans* AS2.538 and *A. niger* ACCC3005) were used to determine the antibacterial activities of pure compounds. The spores of indicator organisms were diluted to 1 × 10^6^ spores/mL and poured into a Petri dish (ϕ = 9 cm). Fungal indicators were cultured in PDA medium at 25 °C, and bacterial indicators were cultured in LB medium at 28 °C. Five microliters of compound solution at 10 mg/mL was added to sterilized filter paper (ϕ = 5 mm) on the surface of the solidified medium. After 24–48 h of culture, the inhibition diameter was measured. Amphotericin B and gentamicin were used as positive controls for fungal strains and bacterial strains, respectively. To qualitatively describe the results, the term of moderate activity means the compound has the inhibition zone with the close size of the filter paper (ϕ = 5 mm). 

### 4.7. Identification of Metabolites

Phomopchalasin C_1_ (**1**). White powder; [α]^24.3 D^ +30° (*c* 0.10, MeOH); UV (MeOH) λ_max_ 237 nm; IR (KBr) *ν*_max_ 3266, 2925, 1697, 1454, 732 cm^−1^; NMR data see Table 1 and Table S1; (+)-HRESIMS *m/z* 434.2682 [M + H]^+^ (calcd for C_28_H_36_NO_3_: 434.2690).

Phomopchalasin C_2_ (**2**). White powder; [α]^24.3 D^ +38.2° (*c* 1.00, MeOH); UV (MeOH) λ_max_ 259 nm; IR (KBr) *ν*_max_ 3346, 2924, 1697, 1454, 1379, 1062, 1030, 700 cm^−1^; NMR data see Table 1 and Table S2; (+)-HRESIMS *m/z* 450.2629 [M + H]^+^ (calcd for C_28_H_36_NO_4_: 434.2639).

Phomopchalasin C_3_ (**3**). White crystal; [α]^24.3 D^ +77.2° (*c* 0.10, MeOH); UV (MeOH) λ_max_ 260 nm; IR (KBr) *ν*_max_ 3384, 1697, 1215, 1030, 701 cm^−1^; NMR data see Table S3; (+)-HRESIMS *m/z* 434.2689 [M + H]^+^ (calcd for C_28_H_36_NO_3_: 434.2690).

Phomopchalasin C_4_ (**4**). White crystal; [α]^24.3 D^ +24.0° (*c* 0.10, MeOH); UV (MeOH) λ_max_ 252 nm; IR (KBr) *ν*_max_ 3384, 2851, 1682, 1030, 755, 700 cm^−1^; NMR data see Table S4; (+)-HRESIMS *m/z* 434.2686 [M + H]^+^ (calcd for C_28_H_36_NO_3_: 434.2690).

Phomopchalasin C_5_ (**5**). White powder; [α]^24.3 D^ −94° (*c* 0.20, MeOH); UV (ET) λ_max_ 210 nm; IR (KBr) *ν*_max_ 3345, 1684, 1455, 1015, 702 cm^−1^; NMR data see Table S5; (+)-HRESIMS *m/z* 470.2897 [M + H]^+^ (calcd for C_28_H_40_NO_5_: 470.2901).

Phomopchalasin C_6_ (**6**). White powder; [α]^24.3 D^ −48.6° (*c* 1.00, MeOH); UV (MeOH) λ_max_ 260 nm; IR (KBr) *ν*_max_ 3392, 2924, 1684, 1456, 1373, 965, 755, 700 cm^−1^; NMR data see Table S6; (+)-HRESIMS *m/z* 452.2799 [M + H]^+^ (calcd for C_28_H_38_NO_4_: 452.2795).

Phomopchalasin C_7_ (**7**). White powder; [α]^24.3 D^ +24.0° (*c* 0.10, MeOH); UV (MeOH) λ_max_ 361, 259 nm; IR (KBr) *ν*_max_ 3398, 3026, 2924, 1697, 1453, 1378, 700 cm^−1^; NMR data see Table S7; (+)-HRESIMS *m/z* 512.3000 [M + H]^+^ (calcd for C_30_H_42_NO_6_: 512.3007).

Phomopchalasin C_8_ (**8**). White powder; [α]^28.9 D^ +34.4° (*c* 0.10, MeOH); UV (MeOH) λ_max_ 280 nm; IR (KBr) *ν*_max_ 3419, 1738, 1177, 1053 cm^−1^; NMR data see Table S8; (+)-HRESIMS *m/z* 466.2952 [M + H]^+^ (calcd for C_29_H_40_NO_4_: 466.2952).

## Figures and Tables

**Table 1:** NMR spectroscopic data (600 MHz, CDCl_3_) for phomopchalasin C_1_ (**1**) and phomopchalasin C_2_ (**2**).

Position	Phomopchalasin C_1_ (1)			Phomopchalasin C_2_ (2)	
	δ_C_, Type	δ_H_ (*J* in Hz)	HMBC (H→C)	δ_C_, Type	δ_H_ (*J* in Hz)
1	176.0, C			176.5, C	
2		5.70, s	4, 9		6.12, s
3	58.5, CH	3.46, dd (6.7, 7.7)	1, 4, 7	59.3, CH	3.44, dd (6.5,7.3)
4	49.1, CH	3.03, s	1, 5, 9, 10	48.7, CH	3.09, s
5	126.9, C			126.8, C	
6	135.0, C			134.4, C	
7	77.2, CH	4.07, d (10.7)		76.3, CH	3.96, dd (1.1,11.0)
8	47.1, CH	2.03, m		45.5, CH	2.17, dd (9.8,14.4)
9	47.3, C			47.6, C	
10	45.4, CH_2_	2.81, dd (8.1, 13.3)	3, 2′	45.4, CH_2_	2.81, dd (7.7, 13.3)
		2.92, dd (6.1, 13.3)	1′		2.88, dd (6.5, 13.3)
11	16.9, CH_3_	1.50, s	4, 5, 6	16.9, CH_3_	1.44, s
12	13.5, CH_3_	1.78, s	5, 6, 7	13.5, CH_3_	1.75, s
13	45.3, CH	2.14, t (9.7)		42.9, CH	2.01, m
14	87.9, CH	3.66, ddd (2.9, 9.2, 12.0)		89.3, CH	3.63, ddd (3.3, 9.6, 11.8)
15	40.4, CH_2_	1.46, d (11.6)		39.1, CH_2_	1.28, dd (3.0, 11.4)
		2.01, m			1.87, d (11.5)
16	30.5, CH	2.15, dd (7.1, 9.7)		29.5, CH	2.07, m
17	133.7, CH	5.29, s		135.1, CH	5.30, d (1.8)
18	137.5, C			138.4, C	
19	34.6, CH	2.32, dd (3.3, 11.7)		40.8, CH	2.30, t (10.8)
20	34.4, CH_2_	2.07, dt (3.3, 13.7)	13	71.8, CH	4.57, dd (3.2,10.2)
		2.38, td (2.3, 13.5)			
21	70.3, CH	3.96, d (2.7)		73.8, CH	3.81, d (3.3)
22	24.6, CH_3_	1.14, d (7.1)	15, 16, 17	23.9, CH_3_	1.09, d (7.1)
23	23.8, CH_3_	1.77, s	17, 18, 19	22.8, CH_3_	2.00, s
1′	137.0, C			137.0, C	
2′, 6′	129.4, CH	7.21, d (7.0)	10, 3′, 4′	129.4, CH	7.22, d (7.1)
3′, 5′	128.8, CH	7.36, t (7.2)	1′	128.8, CH	7.34, t (7.5)
4′	127.1, CH	7.29, d (7.2)		127.1, CH	7.28, t (7.5)
OH (20)					3.35, s
OH (21)		1.64, brs			2.58, s

**Table 2:** Antimicrobial activities of compound **1**, **3**, and **4**.

	Inhibition Zone Diameter (mm)			
Compound	*B. subtilis*	*B. pumilus*	*C. albicans*	*A. niger*
**1**	/	/	/	8
**3**	/	9	10	/
**4**	7	8	/	/
Gentamicin	16	16	/	/
Amphotericin B	/	/	15	15

Note: ‘/’ stands for inactive.

## Data Availability

The data presented in this study are available on request from the corresponding author.

## Supplementary Materials

The Supplementary Materials are available online. Figure S1. ^1^H-NMR (600 MHz, CDCl_3_) spectrum of phomopchalasin C_1_ (**1**), Table S1: NMR (600 MHz, CDCl_3_) data for phomopchalasin C_1_ (**1**).

## Sample Availability

Not available.
