# Supplementary material for: Pentacyclic Cytochalasins and Their Derivatives from the Endophytic Fungus Phomopsis sp. xz-18
Source: Molecules. 2021 Oct 28;26(21):6505. doi: 10.3390/molecules26216505 (PMC8587572; doi:10.3390/molecules26216505)
Supplement: Supplementary file 1 [file molecules-26-06505-s001.zip › molecules-1362209-supplementary.pdf]

Supporting Information

Pentacyclic Cytochalasins and Their Relatives from the Endophytic Fungus *Phomopsis* sp. xz-18

Guihong Huang, *Weiwen Lin*, *Hanpeng Li*, *Qian Tang*, *Zhiyu Hu*, *Huiying Huang*, *Xianming Deng**, and *Qingyan Xu**

State Key Laboratory of Cellular Stress Biology, School of Life Sciences, Xiamen University, Xiamen, Fujian 361102, China

State-Province Joint Engineering Laboratory of Targeted Drugs from Natural Products, Xiamen University, Xiamen, Fujian 361102, China

Table of contents

1. Taxonomy of *Phomopsis* sp. xz-18…………………………………………………….S7

2. Tabulated NMR data ……………………………………………………………………..S8

3. NMR spectra………………………………………………………………………….. S18

4. HR-ESI-MS data……………………………………………………………………….S87

5. ECD spectra…………………………………………………………………………..S95

6. Charts……………………………………………………………………………….S101

List of Tables

Table S1. NMR (600 MHz, $CDCl_3$) data for phomopchalasin C_1 (**1**)........................S8

Table S2. NMR (600 MHz, $CDCl_3$) data for phomopchalasin C_2 (**2**)........................S9

Table S3. NMR (600 MHz, $CDCl_3$) data for phomopchalasin C_3 (**3**)........................S11

Table S4. NMR (600 MHz, $CDCl_3$) data for phomopchalasin C_4 (**4**)........................S12

Table S5. NMR (600 MHz, $CDCl_3$) data for phomopchalasin C_5 (**5**)........................S13

Table S6. NMR (600 MHz, $CDCl_3$) data for phomopchalasin C_6 (**6**)........................S14

Table S7. NMR (600 MHz, $CDCl_3$) data for phomopchalasin C_7 (**7**)........................S15

Table S8. NMR (600 MHz, $CDCl_3$) data for phomopchalasin C_8 (**8**)........................S17

List of Figures

Figure S1. ^{1}H-NMR (600 MHz, $CDCl_3$) spectrum of phomopchalasin C_1 (**1**)........... S18

Figure S2. ^{13}C-NMR (150 MHz, $CDCl_3$) spectrum of phomopchalasin C_1 (**1**)...........S19

Figure S3. HSQC spectrum of phomopchalasin C_1 (**1**)......................................S20

Figure S4. HMBC spectrum of phomopchalasin C_1 (**1**)......................................S21

Figure S5. ^{1}H-^{1}H COSY spectrum of phomopchalasin C_1 (**1**)............................... S22

Figure S6. NOE spectrum of phomopchalasin C_1 (**1**)...S23

Figure S7. ^{1}H-NMR (600 MHz, $CDCl_3$) spectrum of phomopchalasin C_2 (**2**).............S24

Figure S8. ^{13}C-NMR (150 MHz, $CDCl_3$) spectrum of phomopchalasin C_2 (**2**)............S25

Figure S9. HSQC spectrum of phomopchalasin C_2 (**2**).......................................S26

Figure S10. HMBC spectrum of phomopchalasin C_2 (**2**)....................................S27

Figure S11. ^{1}H-^{1}H COSY spectrum of phomopchalasin C_2 (**2**)...............................S28

Figure S12. NOE spectrum of phomopchalasin C_2 (**2**).......................................S29

Figure S13. ^{1}H-NMR (600 MHz, $CDCl_3$) spectrum of phomopchalasin C_3 (**3**)............S30

Figure S14. ^{13}C-NMR (150 MHz, $CDCl_3$) spectrum of phomopchalasin C_3 (**3**)...........S31

Figure S15. HSQC spectrum of phomopchalasin C_3 (**3**)......................................S32

Figure S16. HMBC spectrum of phomopchalasin C_3 (**3**)....................................S33

Figure S17. ^{1}H-^{1}H COSY spectrum of phomopchalasin C_3 (**3**)..............................S34

Figure S18. NOE spectrum of phomopchalasin C_3 (**3**).......................................S35

Figure S19. ^{1}H-NMR (600 MHz, $CDCl_3$) spectrum of phomopchalasin C_4 (**4**)…………S36
Figure S20. ^{13}C-NMR (150 MHz, $CDCl_3$) spectrum of phomopchalasin C_4 (**4**)…………S37
Figure S21. HSQC spectrum of phomopchalasin C_4 (**4**)……………………………………S38
Figure S22. HMBC spectrum of phomopchalasin C_4 (**4**)…………………………………..S39
Figure S23. ^{1}H-^{1}H COSY spectrum of phomopchalasin C_4 (**4**)…………………………….S40
Figure S24. NOE spectrum of phomopchalasin C_4 (**4**)……………………………………..S41
Figure S25. ^{1}H-NMR (600 MHz, $CDCl_3$) spectrum of phomopchalasin C_5 (**5**)…………S42
Figure S26. ^{13}C-NMR (150 MHz, $CDCl_3$) spectrum of phomopchalasin C_5 (**5**)…………S43
Figure S27. HSQC spectrum of phomopchalasin C_5 (**5**)……………………………………S44
Figure S28. HMBC spectrum of phomopchalasin C_5 (**5**)…………………………………..S45
Figure S29. ^{1}H-^{1}H COSY spectrum of phomopchalasin C_5 (**5**)…………………………….S46
Figure S30. NOE spectrum of phomopchalasin C_5 (**5**)……………………………………..S47
Figure S31. ^{1}H-NMR (600 MHz, $CDCl_3$) spectrum of phomopchalasin C_6 (**6**)…………S48
Figure S32. ^{13}C-NMR (150 MHz, $CDCl_3$) spectrum of phomopchalasin C_6 (**6**)…………S49
Figure S33. HSQC spectrum of phomopchalasin C_6 (**6**)……………………………………S50
Figure S34. HMBC spectrum of phomopchalasin C_6 (**6**)…………………………………..S51
Figure S35. ^{1}H-^{1}H COSY spectrum of phomopchalasin C_6 (**6**)…………………………….S52
Figure S36. NOE spectrum of phomopchalasin C_6 (**6**)……………………………………..S53
Figure S37. ^{1}H-NMR (600 MHz, $CDCl_3$) spectrum of phomopchalasin C_7 (**7**)…………S54
Figure S38. ^{13}C-NMR (150 MHz, $CDCl_3$) spectrum of phomopchalasin C_7 (**7**)…………S55
Figure S39. HSQC spectrum of phomopchalasin C_7 (**7**)………………………………….S56
Figure S40. HMBC spectrum of phomopchalasin C_7 (**7**)…………………………………..S57
Figure S41. ^{1}H-^{1}H COSY spectrum of phomopchalasin C_7 (**7**)…………………………….S58
Figure S42. NOE spectrum of phomopchalasin C_7 (**7**)……………………………………..S59
Figure S43. ^{1}H-NMR (600 MHz, $CDCl_3$) spectrum of phomopchalasin C_8 (**8**)…………S60
Figure S44. ^{13}C-NMR (150 MHz, $CDCl_3$) spectrum of phomopchalasin C_8 (**8**)…………S61
Figure S45. HSQC spectrum of phomopchalasin C_8 (**8**)……………………………………S62
Figure S46. HMBC spectrum of phomopchalasin C_8 (**8**)…………………………………..S63
Figure S47. ^{1}H-^{1}H COSY spectrum of phomopchalasin C_8 (**8**)…………………………….S64

Figure S48. NOE spectrum of phomopchalasin C_8 (**8**)……………………………………..S65
Figure S49. ^{1}H-NMR (600 MHz, $CDCl_3$) spectrum of cytochalasin J_3 (**9**)………………S66
Figure S50. ^{13}C-NMR (150 MHz, $CDCl_3$) spectrum of cytochalasin J_3 (**9**)……………..S67
Figure S51. ^{1}H-NMR (600 MHz, $CDCl_3$) spectrum of phomopchalasin B (**10**)…………S68
Figure S52. ^{13}C-NMR (150 MHz, $CDCl_3$) spectrum of phomopchalasin B (**10**)………..S69
Figure S53. ^{1}H-NMR (600 MHz, $CDCl_3$) spectrum of cytochalasin H (**11**)…………….S70
Figure S54. ^{13}C-NMR (150 MHz, $CDCl_3$) spectrum of cytochalasin H (**11**)……………S71
Figure S55. ^{1}H-NMR (600 MHz, $CDCl_3$) spectrum of cytochalasin J (**12**)……………..S72
Figure S56. ^{13}C-NMR (150 MHz, $CDCl_3$) spectrum of cytochalasin J (**12**)…………….S73
Figure S57. ^{1}H-NMR (600 MHz, $CDCl_3$) spectrum of cytochalasin N (**13**)…………….S74
Figure S58. ^{13}C-NMR (150 MHz, $CDCl_3$) spectrum of cytochalasin N (**13**)……………S75
Figure S59. ^{1}H-NMR (600 MHz, $CDCl_3$) spectrum of cytochalasin O (**14**)…………….S76
Figure S60. ^{13}C-NMR (150 MHz, $CDCl_3$) spectrum of cytochalasin O (**14**)……………S77
Figure S61. ^{1}H-NMR (600 MHz, $CDCl_3$) spectrum of cytochalasin N (**15**)…………….S78
Figure S62. ^{13}C-NMR (150 MHz, $CDCl_3$) spectrum of cytochalasin N (**15**)……………S79
Figure S63. ^{1}H-NMR (600 MHz, $CDCl_3$) spectrum of cytochalasin Q (**16**)…………….S80
Figure S64. ^{13}C-NMR (150 MHz, $CDCl_3$) spectrum of cytochalasin Q (**16**)……………S81
Figure S65. ^{1}H-NMR (600 MHz, $CDCl_3$) spectrum of cytochalasin R (**17**)……………..S82
Figure S66. ^{13}C-NMR (150 MHz, $CDCl_3$) spectrum of cytochalasin R (**17**)……………S83
Figure S67. ^{1}H-NMR (600 MHz, $CDCl_3$) spectrum of cytochalasin R1 (**18**)……………S84
Figure S68. ^{13}C-NMR (150 MHz, $CDCl_3$) spectrum of cytochalasin R1 (**18**)…………S85
Figure S69. ^{1}H-NMR (600 MHz, $CDCl_3$) spectrum of acetylated phomopchalasin C_2 (**2A**)
…………………………………………………………………………………S86
Figure S70. HR-ESI-MS spectrum of phomopchalasin C_1 (**1**)…………………………..S87
Figure S71. HR-ESI-MS spectrum of phomopchalasin C_2 (**2**)…………………………..S88
Figure S72. HR-ESI-MS spectrum of phomopchalasin C_3 (**3**)…………………………..S89
Figure S73. HR-ESI-MS spectrum of phomopchalasin C_4 (**4**)…………………………..S90
Figure S74. HR-ESI-MS spectrum of phomopchalasin C_5 (**5**)…………………………..S91
Figure S75. HR-ESI-MS spectrum of phomopchalasin C_6 (**6**)…………………………..S92

Figure S76. HR-ESI-MS spectrum of phomopchalasin C_7 (**7**)……………………………..S93

Figure S77. HR-ESI-MS spectrum of phomopchalasin C_8 (**8**)……………………………..S94

Figure S78. ECD spectrum of phomopchalasin C_1 (**1**)……………………………………..S95

Figure S79. ECD spectrum of phomopchalasin C_2 (**2**)……………………………………..S95

Figure S80. ECD spectrum of phomopchalasin C_3 (**3**)……………………………………..S96

Figure S81. ECD spectrum of phomopchalasin C_4 (**4**)……………………………………..S96

Figure S82. ECD spectrum of phomopchalasin C_5 (**5**)……………………………………..S97

Figure S83. ECD spectrum of phomopchalasin C_6 (**6**)……………………………………..S97

Figure S84. ECD spectrum of phomopchalasin C_7 (**7**)……………………………………..S98

Figure S85. ECD spectrum of phomopchalasin C_8 (**8**)……………………………………..S98

Figure S86. Cotton effect-spiral chirality correlation for compound **3** and **4**……………S99

Figure S87. Illustration of the viewpoint of octant rule for compound **6**……………….S100

List of Charts

Chart S1. Structure of compound **9-18**…………………………………………………S101

1. Taxonomy of *Phomopsis* sp. xz-18

Genomic DNA of *phomopsis* sp. xz-18 was extracted from the mycelia using CTAB (hexadecyltrimethylammonium ammonium bromide) method. The rRNA genes were amplified by PCR using then universal primers (purchased from Sigma-Aldrich):

ITS1: 5’-TCCGTAGGTGAACCTGCGG-3’

ITS4: 5’-TCCTCCGCTTATTGATATGC-3’

PCR reaction system (20 μL):

10 × PCR buffer	2 μL
ITS1 (10 mmol/L)	1 μL
ITS4 (10 mmol/L)	1 μL
dNTP (2.5 mmol/L)	0.5 μL
Taq enzyme	0.2 μL
DNA template	0.5 μL
dd H_2O	14.8 μL

PCR amplification program:

Initial denaturation: 95 °C for 4 min

Denaturation: 95°C for 20 sec

Annealing: 55°C for 20 sec

Extension: 72°C for 30 sec (above three steps repeated 35 times)

Extension: 72°C for 7 min.

The BLAST in NCBI showed the ITS gene sequence of *Phomopsis* sp. xz-18:

TTTCCTCCCGGCCCTTTGATATGCTTAAGTTCAGCGGGTATTCCTACCTGATCCGAGGTCAAATTTT
CAGAAGTTGGGGGTTTAACGGCAGGGCACCGCCAGGGCCTTCCAGAACGAGATATAACTACTACG
CTCGGGGTCCTAGCGAGCTCGCCACTAGATTTCAGGGCCTGCTCTCGTCAGAGAGCAGTGCCCCA
TCACCAAGCCAGGCTTGAGGGTTGAAATGACGCTCGAACAGGCATGCCCTCCGGAATACCAGAG
GGCGCAATGTGCGTTCAAAGATTCGATGATTCACTGAATTCTGCAATTCACATTACTTATCGCATTT
CGCTGCGTTCTTCATCGATGCCAGAACCAAGAGATCCGTTGTTGAAAGTTTTGATTCATTTATGTT
TTTTACTCAGAGATTCACTGTAGAAACAAGAGTTTGGTTGGCCGCCGGCGGGCTGCTCCCTGTCT
CCAGGGGGCCTCAGTGAAGAGGCCGGCCTGCGCCGAGGCAACAATAGGTATAAGTTCACAAAGG
GTTTCTGGGTGCC

2. Tabulated NMR data

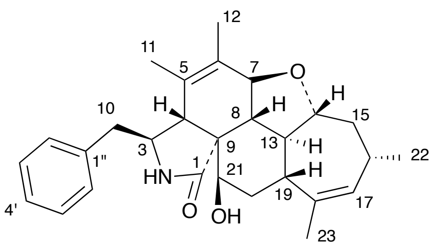

Table S1. NMR Data of phomopchalasin C_1（600 MHz, $CDCl_3$，δ in ppm ）

No.	^{1}H (mult.,J in Hz)	^{13}C, type	HMBC	COSY	NOE
1	/	176.0 s	/	/	/
2	5.70 (s)	/	C-4, C-9	H-3, H-4(w)	H-2'/6'
3	3.46 (dd, 6.7, 7.7)	58.5 d	C-1, C-4, C-5	H-4, H_2-10	H-2, H_3-11
4	3.03 (s)	49.1 d	C-1, C-5, C-9, C-10, C-21	H-2, H-3, H_3-11	H-8, H-21, H-2'/6'
5	/	126.9 s	/	/	/
6	/	135.0 s	/	/	/
7	4.07 (d, 10.7)	77.2 d	/	H-8, H_3-11, H_3-12	H-13
8	2.03 (m)	47.1 d	C-7	H-7, H-13, H-14	H-4,
9	/	47.3 s	/	/	/
10	2.81 (dd, 8.1, 13.3)<br>2.92 (dd, 6.1, 13.3)	45.4 t	C-3, C-1', C-2'/6'	H-3	H-21
11	1.50 (s)	16.9 q	C-4, C-5, C-6	/	H-3
12	1.78 (s)	13.5 q	C-5, C-6, C-7	/	/
13	2.14 (dd, 9.7, 9.8)	45.3 d	/	H-8, H-19	H-7
14	3.66 (ddd, 2.9, 9.2, 12.0)	87.9 d	/	H-13, H_2-15, H-16	H-19
15	1.46 (dd, 11.6)<br>2.01 (m)	40.4 t	/	H-16<br>H-14	/
16	2.15 (dd, 7.1, 9.7)	30.5 d	/	H-17, H_3-22	H-14
17	5.29 (s)	133.7 d	/	H-19, H_3-23	H_3-22
18	/	137.5 s	/	/	/
19	2.32 (dd, 3.3, 11.7)	34.6 d	/	H_2-20, H_3-23	H-14
20	2.07 (dt, 3.3, 13.7)<br>2.38 (td, 2.3, 13.5)	34.4 t	C-13<br>/	H-21<br>/	/<br>/
21	3.96 (dd, 2.7)	70.3 d	/	H_2-20	H-4
22	1.14 (d, 7.1)	24.6 q	C-15, C-16, C-17	H-16	H-17
23	1.77 (s)	23.8 q	C-17, C-18, C-19	H-17, H-19	/
1'	/	137.0 s	/	/	/
2'/6'	7.21 (dd, 7.0)	129.4 d	C-10, C-3', C-4'	H-3'/5'	/
3'/5'	7.36 (dd, 7.2)	128.8 d	C-1'	H-2'/6'	/
4'	7.29 (dd, 7.2)	127.1 d	/	H-3'/5'	/
21-OH	1.64 (brs)	/	/	/	H-21

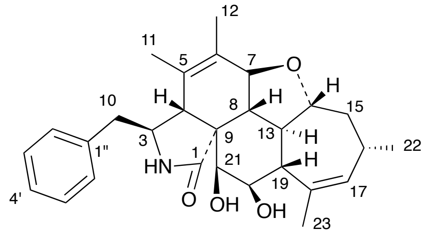

Table S2. NMR Data of phomopchalasin C$_2$（600 MHz, CDCl$_3$，δ in ppm ）

No.	^{1}H (mult.,J in Hz)	^{13}C type	HMBC	COSY	NOE
1	/	176.5 s	/	/	/
2	6.12 (s)	/	C-1, C-3, C-4, C-9	H-3, H-4	/
3	3.44 (dd, 6.5, 7.3)	59.3 d	C-1, C-4, C-5, C-9	H-4, H$_2$-10	H$_3$-11
4	3.09 (s)	48.7 d	C-1, C-3, C-5, C-6, C-9, C-10, C-11	H-2, H-3, H$_3$-11	H-8, H$_2$-10, H-21
5	/	126.8 s	/	/	/
6	/	134.4 s	/	/	/
7	3.96 (dd, 1.1, 11.0)	76.3 d	C-5, C-8	H$_3$-11, H$_3$-12, H-8, H-4	H-13
8	2.17 (dd, 9.8, 14.4)	45.5 d	C-1, C-6, C-7, C-9, C-13, C-14, C-21	H-13, H-7	H-4, H-14, H-19
9	/	47.6 s	/	/	/
10	2.81 (dd, 7.7, 13.3)<br>2.88 (dd, 6.5, 13.3)	45.4 t	C-3, C-4, C-1’, C-2’/6’	H$_2$-10, H-3	H-2’/6’
11	1.44 (s)	16.9 q	C-4, C-5, C-6	H$_3$-12, H-4	H-3
12	1.75 (s)	13.5 q	C-5, C-6, C-7,	H$_3$-11, H-4	/
13	2.01 (m)	42.9 d	C-8, C-14, C-15, C-19	H-8, H-19, H-14	H-7, H$_2$-15, H-20
14	3.63 (ddd, 3.3, 9.6, 11.8)	89.3 d	/	H$_2$-15, H-13	H-8, H-16, H-19
15	1.28 (dd, 3.0, 11.4)<br>1.87 (d, 11.5)	39.1 t	C-13, C-14, C-16, C-17, C-22	H-16, H-14	H-13 (w)
16	2.07 (m)	29.5 d	/	H$_3$-22, H$_2$-15, H-17	H-14
17	5.30 (d, 1.8)	135.1 d	C-16, C-19, C-23	H$_3$-23, H-19	H$_3$-22
18	/	138.4 s	/	/	/
19	2.30 (t, 10.8)	40.8 d	C-13, C-14, C-17, C-18, C-20	H$_3$-23, H-13, H-20, H-17	H-8, H-14
20	4.57 (dd, 3.2, 10.2)	71.8 d	C-18, C-19	H-19, H-21	H-13
21	3.81 (d, 3.3)	73.8 d	C-1, C-8, C-9 (w), C-19, C-20	H-20	H-4, OH-20
22	1.09 (d, 7.1)	23.9 q	C-15, C-16, C-17	H-16	H-17
23	2.00 (s)	22.8 q	C-17, C-18, C-19	H-19, H-14, H-17, H-16	H-20
1’	/	137.0 s	/	/	/
2’/6’	7.22 (d, 7.1)	129.4 d	C-3, C-10, C-1’, C-3’/5’, C-4’	H-3’, H-5’	H$_2$-10

3’/5’	7.34 (t, 7.5)	128.8 d	C-1’, C-2’/6’, C-4’	H-2’/6’, H-4’	/
4’	7.28 (t, 7.5)	127.1 d	C-1’ (w)	H-3’/5’	
20-OH	3.35 (s)	/	/	/	OH-21, H-21
21-OH	2.58 (s)	/	/	/	OH-20

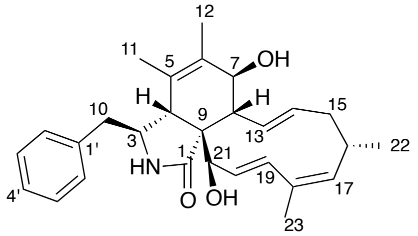

Table S3. NMR Data of phomopchalasin C_3（600 MHz, $CDCl_3$，δ in ppm ）

N$_O$.	^{1}H (mult.,*J* in Hz)	^{13}Ctype	HMBC	COSY	NOE
1	/	176.2 s	/	/	/
2	5.72 (m)	/	C-3, C-4, C-9	/	H-21
3	3.36 (dd, 7.0, 8.9)	60.4 d	C-1, C-5, C-9, C-1’	H-4, H_2-10	H-12, H-14
4	2.99 (s)	50.8 d	C-1, C-3, C-5, C-6, C-9, C-10, C-21	H-3, H_3-11, H_3-12	H-8, H-21
5	/	126.5 s	/	/	/
6	/	131.7 s	/	/	/
7	3.70 (d, 9.9)	67.7 d	C-5, C-6, C-13	H-8, OH-7	H-13
8	2.63 (t, 10.1)	49.1 d	C-1, C-7, C-9, C-13, C-14, C-21	H-7, H-13	OH-7, H-14
9	/	48.7 s	/	/	/
10	2.90 (dd, 9.5, 13.3)<br>3.03 (dd, 5.2, 13.3)	45.6 t	C-3, C-4, C-2’/6’	H-3	H-21
11	1.71 (s)	13.9 q	C-4, C-5, C-6	H-4, H_3-12	H-8
12	1.56 (s)	17.1 q	C-4, C-5, C-6, C-7	H-7, H_3-11	H-3
13	6.18 (dd, 10.3, 15.7)	131.1 d	C-7, C-8, C-15	H-8, H-14	H-7
14	5.65 (ddd, 4.8,10.2, 16.2)	137.2 d	C-8, C-15	H-13, H_2-15	H-3, H-8, H-16 (w)
15	1.97 (dd, 4.7, 12.7)<br>2.27 (m)	43.0 t	C-14, C-16, C-17, C-22	H-13, H-14	H_3-22
16	2.78 (m)	31.6 d	C-14, C-15, C-17, C-18, C-22	H_2-15, H-17, H_3-22	H-14, H-19
17	5.29 (d, 7.3)	136.0 d	C-19, C-22, C-23	H-16, H_3-23	H_3-22
18	/	132.4 s	/	/	/
19	6.90 (d, 16.5)	135.7 d	C-17, C-18, C-20, C-21, C-23	H-20, H-21	H-16, OH-21
20	5.73 (dd, 3.1, 16.5)	124.8 d	C-18, C-21	H-19, H-21	H_3-23
21	4.63 (s)	75.2 d	C-4, C-8, C-19, C-20	H-19, H-20, OH-21	H-2, H-4
22	1.05 (d, 6.8)	24.0 q	C-15, C-16, C-17	H-16	H-17
23	1.87 (s)	20.9 q	C-17, C-18, C-19	H-16, H-17	H-13, H-20
1’	/	137.7 s	/	/	/
2’/6’	7.21 (d, 7.1)	129.1 d	C-10, C-1’, C-3’/5’	H-3’/5’	H-3
3’/ 5’	7.35 (t, 7.4)	128.9 d	C-1’, C-2’/6’	H-2’/6’, H-4’	/
4’	7.28 (d, 7.2)	127.1 d	C-2’/6’, C-3’/5’	H-3’/5’	/
7-OH	2.00 (s)	/	C-5, C-6, C-7, C-9	H-7	H-8, H-19
21-OH	2.19 (s)	/	C-9, C-20, C-21	H-21	H-8, H-19

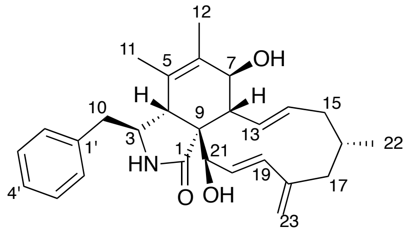

Table S4. NMR Data of phomopchalasin C$_4$（600 MHz, CDCl$_3$，δ in ppm ）

No.	^{1}H (mult.,*J* in Hz)	^{13}C type	HMBC	COSY	NOE
1	/	176.0 s	/	/	/
2	5.54 (s)	/	C-4, C-9	/	H-3
3	3.35 (dd, 4.9, 9.7)	60.7 d	/	H-4, H$_2$-10	H-7
4	3.09 (d, 4.6)	50.7 d	C-3, C-5, C-6, C-10, C-21	H-3, H$_3$-12	H$_3$-11, H-21
5	/	127.0 s	/	/	/
6	/	132.0 s	/	/	/
7	3.84 (H, d, 9.7)	68.1 d	/	H-8, H$_3$-12	H-3, H-13
8	2.15 (t, 10.2)	50.3 d	C-1, C-7, C-9, C-13, C-14, C-21	H-7, H-13	H-14, H$_3$-11
9	/	53.3 s	/	/	/
10	2.71 (dd, 9.7, 13.5)<br>3.13 (dd, 4.3, 13.4)	45.0 t	C-3, C-1’, C-2’/6’	H-3	/
11	1.77 (s)	18.6 q	C-4, C-5, C-6	/	H-4
12	1.80 (s)	13.3 q	C-5, C-6, C-7	/	/
13	5.93 (dd, 11.3, 15.0)	128.7 d	C-15	H-8, H-14	H-7
14	5.21 (ddd, 3.2, 11.2, 15.5)	141.3 d	/	H-13, H$_2$-15	H-8
15	1.91 (dd, 11.2, 13.3)<br>2.26 (dd, 3.2, 13.9)	43.1 t	C-13, C-16	H-14, H-16	H-13
16	1.62 (m)	40.3 d	/	H$_2$-15, H$_2$-17, H$_3$-22	H-19
17	2.07 (d, 13.4)<br>2.38 (dd, 7.2, 13.6)	38.2 t	C-15, C-16, C-18, C-19, C-22, C-23	H-16, H$_2$-23	/
18	/	149.0 s	/	/	/
19	6.13 (dd, 1.9, 16.4)	127.1 d	C-21, C-23 (w)	H-20, H-21	H-16
20	6.69 (dd, 1.9, 16.4)	134.6 d	C-18, C-21	H-19, H-21	H-13
21	4.40 (d, 7.6)	75.6 d	/	OH-21	H-4, H-20
22	1.13 (d, 7.0)	25.1 q	C-15, C-16, C-17	H-16	H$_2$-17
23	4.98 (s)<br>5.05 (s)	116.0 t	C-17, C-19	/	H-19
1’	/	138.0 s	/	/	/
2’/6’	7.21 (d, 7.4)	129.1 d	C-10, C-3’/5’	H-4’, H-3’/5’	H$_2$-10
3’/5’	7.35 (d, 7.5)	129.0 d	C-1’	H-2’/6’, H-4’	/
4’	7.28 (t, 7.5)	127.1 d	C-3’/C-5’	H-3’/5’	/
7-OH	2.00 (s)	/	C-6	H-7	/
21-OH	1.75 (m)	/	/	H-21	/

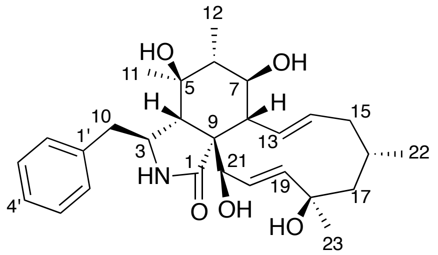

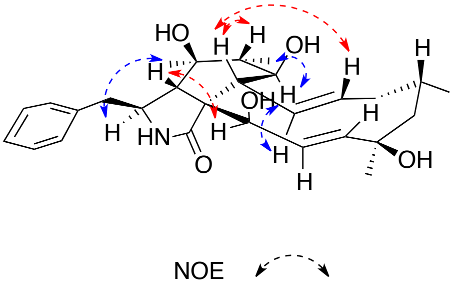

Table S5. NMR Data of phomopchalasin C_5（600 MHz, $CDCl_3$，δ in ppm ）

No.	^{1}H (mult.,J in Hz)	^{13}C type	HMBC	COSY	NOE
1	/	174.3 s	/	/	/
2	5.66 (s)	/	/	/	H-3
3	3.58 (m)	54.3 d	C-5	H-4, H_2-10	H_3-11, H_3-12
4	2.39 (d, 5.5)	58.8 d	C-6, C-10, C-21	H-3, H-6	H-21
5	/	73.1 s	/	/	/
6	1.88 (m)	49.2 d	C-4, C-5, C-7, C-12	H-7, H_3-12	H-8
7	3.08 (dd, 8.0, 11.7)	72.4 d	C-12	H-6	H_3-12, H-13
8	3.38 (dd, 10.3, 11.3)	45.2 d	C-1, C-7, C-19, C-13, C-14	H-7, H-13	H-6, H-14, H-19
9	/	54.9 s	/	/	/
10	3.17 (dd, 2.5, 13.8)<br>2.49 (dd, 9.8, 13.8)	45.7 t	C-3, C-4, C-2’/6’	H-3	H-11
11	1.47 (s)	26.7 q	C-4, C-5, C-6	/	H-3, H-2’
12	1.24 (d, 7.4)	18.3 q	C-5, C-6, C-7	H-6	H-3, H-7
13	5.58 (dd, 9.9, 16.3)	126.0 d	C-8, C-14, C-15	H-8, H-14	H-7
14	5.42 (ddd, 4.4, 10.7, 15.2)	140.6 d	/	H-13, H_2-15	H-8
15	2.06 (m)<br>1.81 (m)	42.9 t	C-13, C-14, C-16, C-17	H-13, H-14	/<br>/
16	1.84 (m)	28.6 d	/	H_2-15, H_3-22	/
17	1.86 (m)<br>1.60 (m)	53.6 t	C-16, C-18, C-19, C-22, C-23	/	/
18	/	74.7 s	/	/	/
19	5.88 (dd, 1.9, 16.5)	137.6 d	C-20, C-21	H-20, H-21	H-16
20	6.02 (dd, 2.9, 16.5)	129.6 d	C-18, C-19, C-21	H-19, H-21	H_3-23
21	4.10 (s)	76.2 d	C-4, C-8, C-9, C-19, C-20	H-19, H-20	H-4
22	1.06 (d, 6.6)	26.5 q	C-15, C-17	H_2-15	/
23	1.37 (s)	31.2 q	C-17, C-18, C-19	/	H-20
1’	/	137.1 s	/	/	/
2’/6’	7.18 (d, 7.4)	129.0 d	C-10, C-4’	H-3’/5’	H-3
3’/5’	7.37 (t, 7.4)	129.1 d	C-1’, C-2’/6’	H-2’/6’	/
4’	7.30 (d, 7.4)	127.3 d	C-2’/6’	H-3’/5’	/

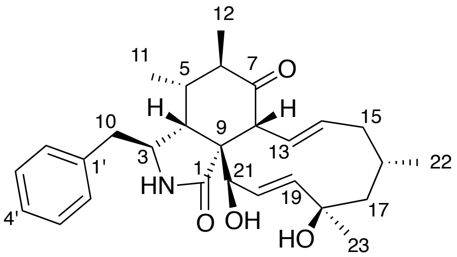

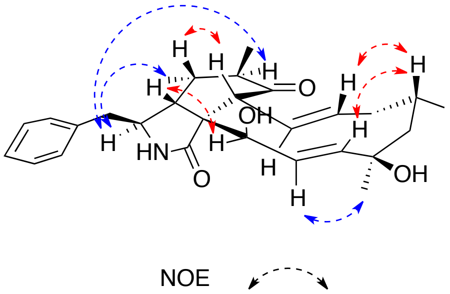

Table S6. NMR Data of phomopchalasin C_6（600 MHz, $CDCl_3$, δ in ppm ）

No.	^{1}H (mult.,*J* in Hz)	^{13}C type	HMBC	COSY	NOE
1	/	174.6 s	/	/	/
2	5.92 (s)	/	C-4, C-9	/	H-3
3	3.59 (m)	53.4 d	/	H_2-10	H-2, H-6, H_3-11
4	2.66 (t, 4.2)	51.2 d	C-5, C-6, C-10, C-21	/	H_3-12
5	2.37 (m)	35.6 d	C-3, C-4, C-6, C-11	H-6, H_3-11	H-8
6	2.04 (m, 7.5, 14.5)	45.9 d	C-5, C-7, C-11	H_3-12	H_3-11
7	/	215.4 s	/	/	/
8	3.91 (d, 9.7)	50.5 d	C-1, C-7, C-13, C-14, C-21	/	H-5, H-14
9	/	54.4 s	/	/	/
10	3.03 (dd, 3.2, 13.6)<br>2.59 (dd, 9.4, 13.6)	46.0 t	C-3, C-1’, C-2’/6’	/	/
11	1.12 (d, 6.9)	16.0 q	C-4, C-5, C-6	/	H-3, H-6
12	1.15 (d, 7.2)	15.9 q	C-5, C-6, C-7	/	H-4
13	5.73 (dd, 10.3, 14.9)	123.8 d	C-8, C-14, C-15	H-8, H-14	/
14	5.15 (m)	137.0 d	/	H_2-15	H-8
15	2.03 (m)<br>1.84 (m)	42.9 t	C-13, C-14, C-16, C-17, C-22	/	/
16	1.76 (m)	28.4 d	C-14, C-17	H_3-22	H-14, H-19
17	1.85 (dd, 3.7, 15.0)<br>1.55 (d, 14.2)	53.6 t	C-15, C-18, C-19, C-22, C-23	/	/
18	/	74.5 s	/	/	/
19	5.72 (d, 15.1)	136.6 d	C-20, C-21, C-23	H-21	H-16
20	6.02 (dd, 2.3, 14.6)	130.6 d	C-18, C-21	H-19, H-21	H_3-23
21	4.18 (s)	76.8 d	C-4, C-8, C-9, C-19, C-20	/	H-4
22	1.04 (d, 6.9)	26.5 q	C-15, C-16, C-17	H-16	/
23	1.34 (s)	31.1 q	C-17, C-18, C-19	/	H-20
1’	/	137.2 s	/	/	/
2’/6’	7.18 (d, 7.4)	129.2 d	C-10, C-4’	H-3’/5’	H-3
3’/5’	7.34 (t, 7.4)	128.9 d	C-1’, C-2’/6’, C3’/5’	H-2’/6’	/
4’	7.27 (d, 7.4)	127.2 d	C-2’/6’, C-3’/5’	H-3’/5’	/

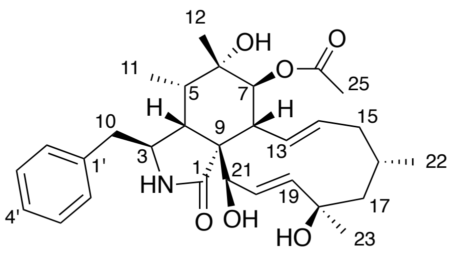

Table S7. NMR Data of phomopchalasin C_7（600 MHz, $CDCl_3$，δ in ppm ）

No.	^{1}H (mult.,*J* in Hz)	^{13}C type	HMBC	COSY	NOE
1	/	175.2 s	/	/	/
2	5.85 (s)	/	C-1	H-3, H-4, H_2-10	H-7
3	3.61 (m)	54.3 d	C-1 (w), C-4, C-5	H-4, H_2-10	H-7, H_3-11
4	2.46 (t, 5.4)	50.5 d	C-1, C-6, C-10, C-21	H-3, H_2-10	H_3-12, H-21
5	2.54 (m)	39.1 d	C-3, C-4, C-6, C-9, C-11, C-12	H-4, H_3-11, H_3-12	H-8
6	/	73.0 s	/	/	/
7	4.59 (d, 12.1)	74.5 d	C-6, C-8, C-9, C-12, C-24	H-8	H-3, H_3-11, H-13
8	3.25 (dd, 10.3, 11.8)	40.8 d	C-1, C-6, C-14, C-21	H-7, H-13	H-5, H-14
9	/	54.5 s	/	/	/
10	2.50 (dd, 9.6, 13.6)<br>3.07 (dd, 2.8, 13.6)	46.4 t	C-3, C-4, C-1', C-3', C-5'	H-3, H-4	/
11	1.23 (d, 7.7)	14.1 q	C-4, C-5, C-6	H-5	H-3, H-7
12	1.22 (s)	25.0 q	C-5, C-6, C-7	H-5	H-4
13	5.40 (dd, 10.7, 15.2)	125.4 d	C-8, C-15	H-8, H-14	H-7
14	5.18 (ddd, 4.3, 10.4, 15.1)	137.7 d	C-15 (w)	H-13, H_2-15, H-16	H-8
15	1.74 (m)<br>1.95 (dd, 4.5, 11.4)	42.5 t	C-13, C-16, C-17 (w)	H-13, H-14, H_3-22	/
16	1.75 (m)	28.8 d	C-14	H-14, H_2-15, H_3-22	H-19
17	1.54 (dd, 1.6, 14.0)<br>1.90 (dd, 3.0, 14.3)	53.3 t	C-15, C-16, C-18, C-19, C-22, C-23	H-16	/
18	/	74.6 s	/	/	/
19	5.72 (dd, 1.9, 16.9)	136.2 d	C-21, C-20, C-23 (w)	H-20, H-21	H-16
20	6.16 (dd, 2.8, 16.8)	131.7 d	C-18, C-19, C-21	H-19, H-21	H_3-23
21	4.14 (t, 2.0)	77.3 d	C-4, C-8, C-9, C-19, C-20	H-19, H-20	H-4
22	1.03 (d, 6.3)	26.5 q	C-15, C-16, C-17	H-16	/
23	1.34 (s)	32.0 q	C-17, C-18, C-19	/	H-20
24	/	170.0 s	/	/	/
25	2.00 (s)	20.9 q	C-7 (w)	/	/

1’	/	137.3 s	/	/	/
2’/6’	7.18 (d, 7.3)	129.1 d	C-10, C-4’, C-2’/6’	H-3’/5’	H-2, H-3
3’/5’	7.35 (t, 7.4)	129.0 d	C-1’, C-3’/5’	H-2’/6’	/
4’	7.28 (d, 7.3)	127.2 d	C-2’/6’, C-3’/5’	H-3’/5’	/

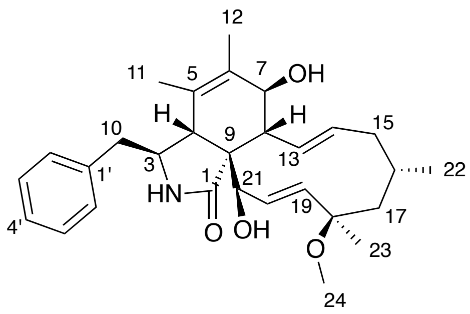

Table S8. NMR Data of phomopchalasin C$_8$（600 MHz, CDCl$_3$，δ in ppm ）

No.	^{1}H (mult.,*J* in Hz)	^{13}C type	HMBC	COSY	NOE
1	/	176.2 s	/	/	/
2	5.58 (s)	/	/	H-3	/
3	3.38 (m)	60.3 d	/	H-4, H$_2$-10	H$_3$-11
4	3.07 (s)	50.5 d	C-1, C-6, C-10, C-21	H$_3$-11, H$_3$-12	H-8, H-21
5	/	126.7 s	/	/	/
6	/	131.5 s	/	/	/
7	3.85 (d, 9.4)	68.3 d	/	H-8, H$_3$-12, OH-7	H-13
8	2.49 (t, 10.1)	48.8 d	C-1, C-4, C-7, C-13, C-14	H-7, H-13	OH-7, H-4, H-14
9	/	54.2 s	/	/	/
10	3.04 (dd, 5.7, 13.4)<br>2.87 (dd, 9.7, 13.4)	45.6 t	C-3, C-1', C-2'/6'	H-3	/
11	1.58 (s)	17.2 q	C-4, C-5, C-6	H-4, H-7	H-3
12	1.73 (s)	13.9 q	C-3, C-5, C-6, C-7	H-4, H-7	/
13	5.99 (dd, 10.3, 15.8)	128.3 d	C-8, C-15	H-8, H-14	H-7
14	5.36 (m)	137.5 d	/	H$_2$-15, H-16	H-8, OH-21
15	1.99 (dd, 6.1, 12.4)	42.8 t	C-13, C-14, C-17	H-16	/
16	1.80 (m)	27.8 d	/	H-14, H$_2$-15	/
17	1.75 (brs)	52.0 t	C-15, C-18, C-19, C-22	/	/
18	/	79.0 s	/	/	/
19	5.82 (dd, 2.3, 16.8)	137.4 d	C-20, C-21	H-20, H-21	/
20	5.94 (dd, 2.5, 16.7)	131.2 d	C-18, C-21	H-19, H-21	H$_3$-23
21	4.51 (d, 7.1)	75.3 d	C-8, C-19, C-20	OH-21	H-4
22	1.04 (d, 6.2)	26.1 q	C-15, C-16, C-17	H-16，H$_2$-17	/
23	1.34 (s)	24.1 q	C-17, C-18, C-19	/	/
24	3.25 (s)	50.6 q	C-18	/	H-19
1'	/	137.6 s	/	/	/
2'/6'	7.21 (d, 7.5)	129.1 d	C-4', C-3'/5', C-10	H-3'/5'	/
3'/ 5'	7.36 (t, 7.5)	128.9 d	C-1'	H-2'/6', H-4'	/
4'	7.28 (d, 7.5)	127.1 d	/	/	/
7-OH	1.97 (s)	/	C-6, C-7, C-8	H-7	H-14
21-OH	1.91 (d, 7.4)	/	C-21	H-21	H-8, H-19

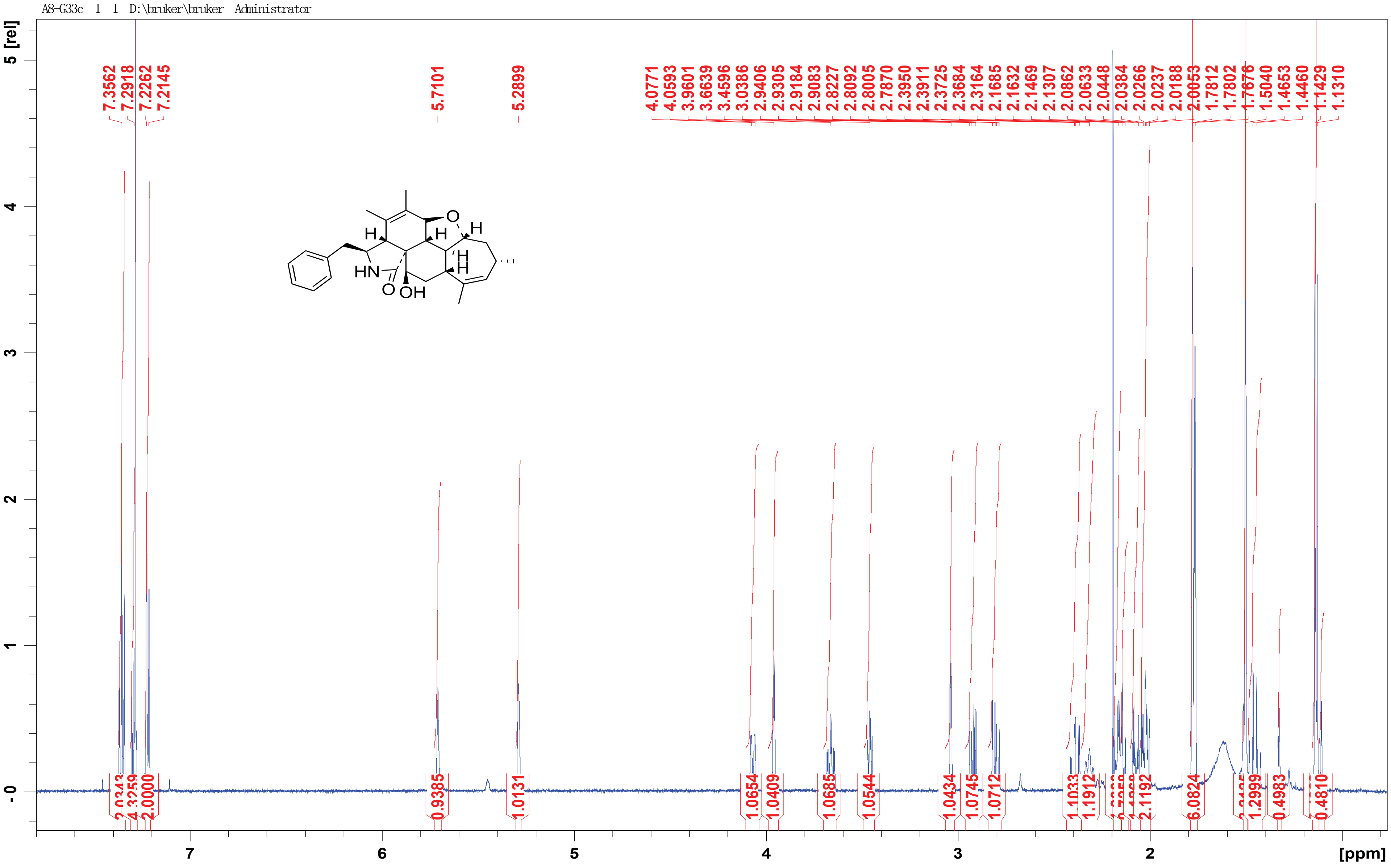

Figure S1. ^{1}H-NMR (600 MHz, $CDCl_3$) spectrum of phomopchalasin C_1 (**1**)

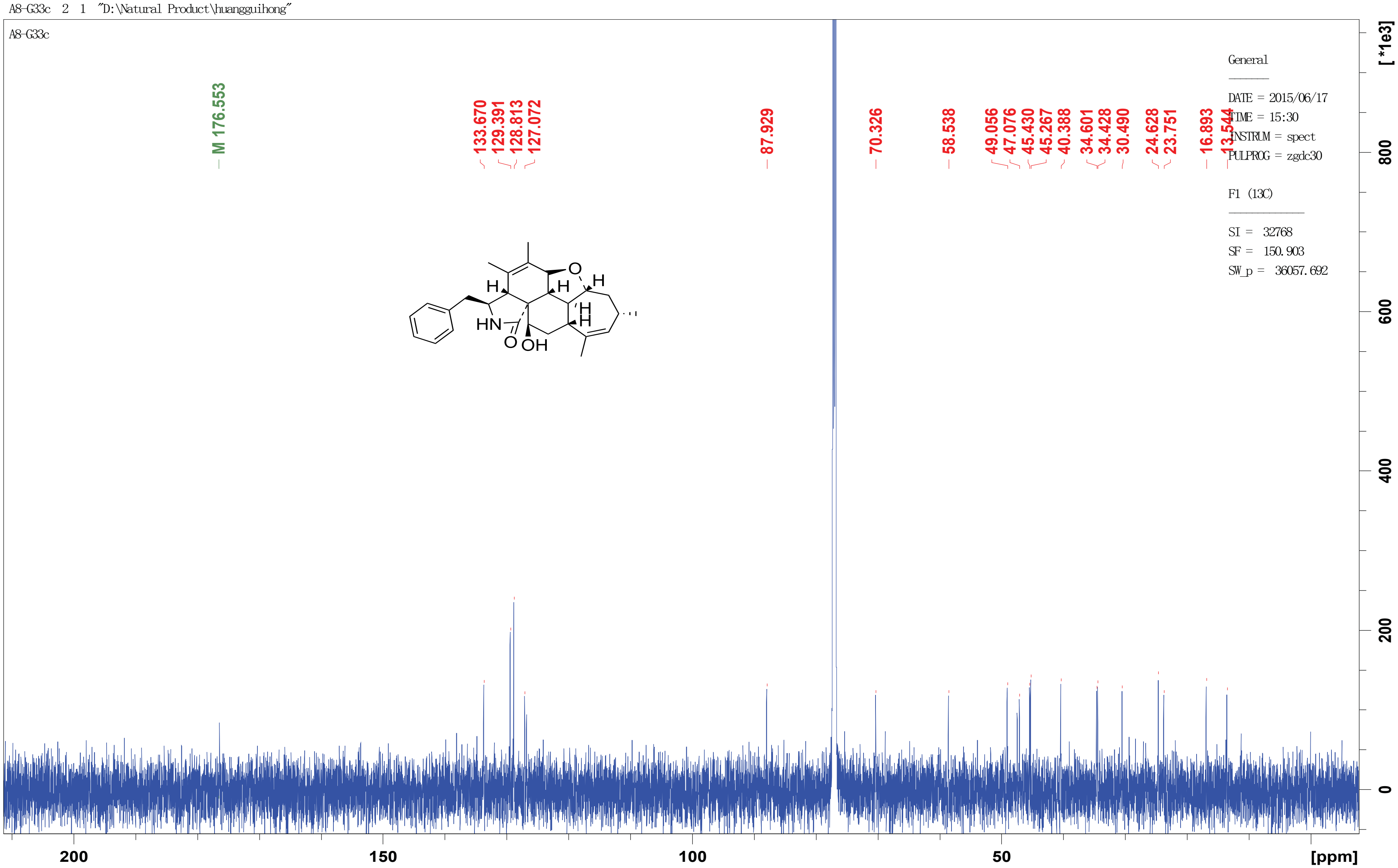

Figure S2. ^{13}C-NMR (150 MHz, $CDCl_3$) spectrum of phomopchalasin C_1 (**1**)

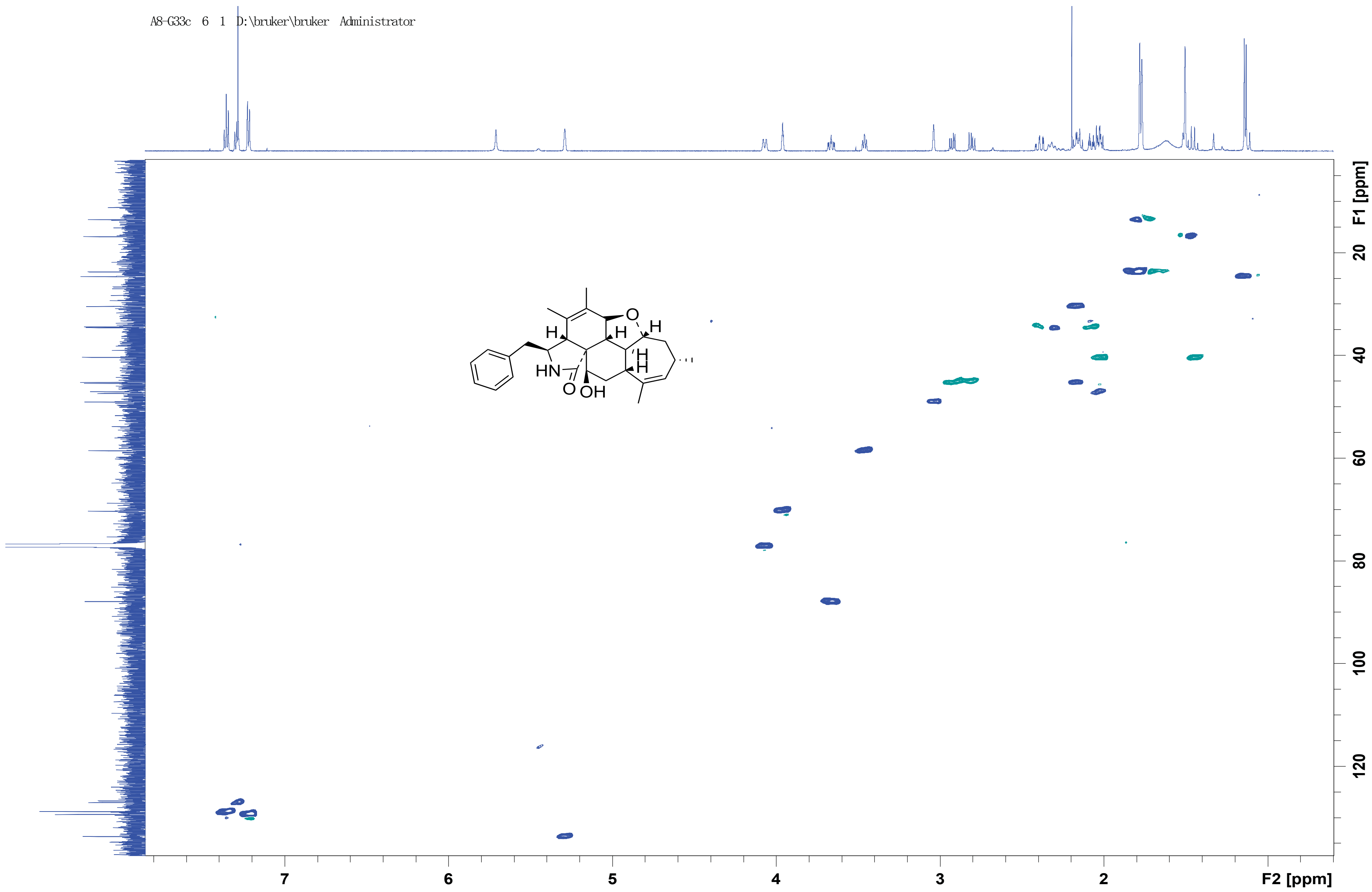

Figure S3. HSQC spectrum of phomopchalasin C_1 (**1**)

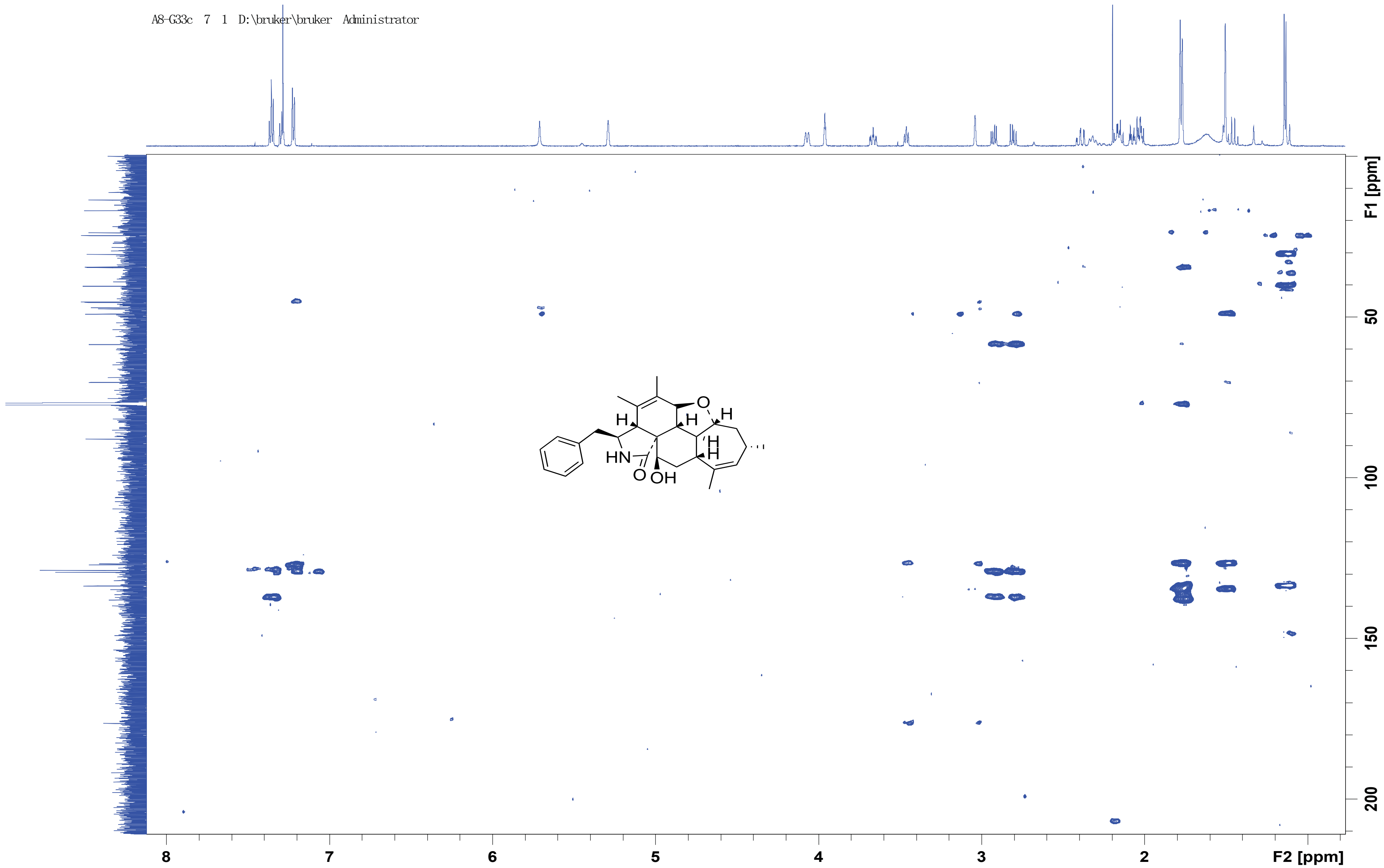

Figure S4. HMBC spectrum of phomopchalasin C_1 (**1**)

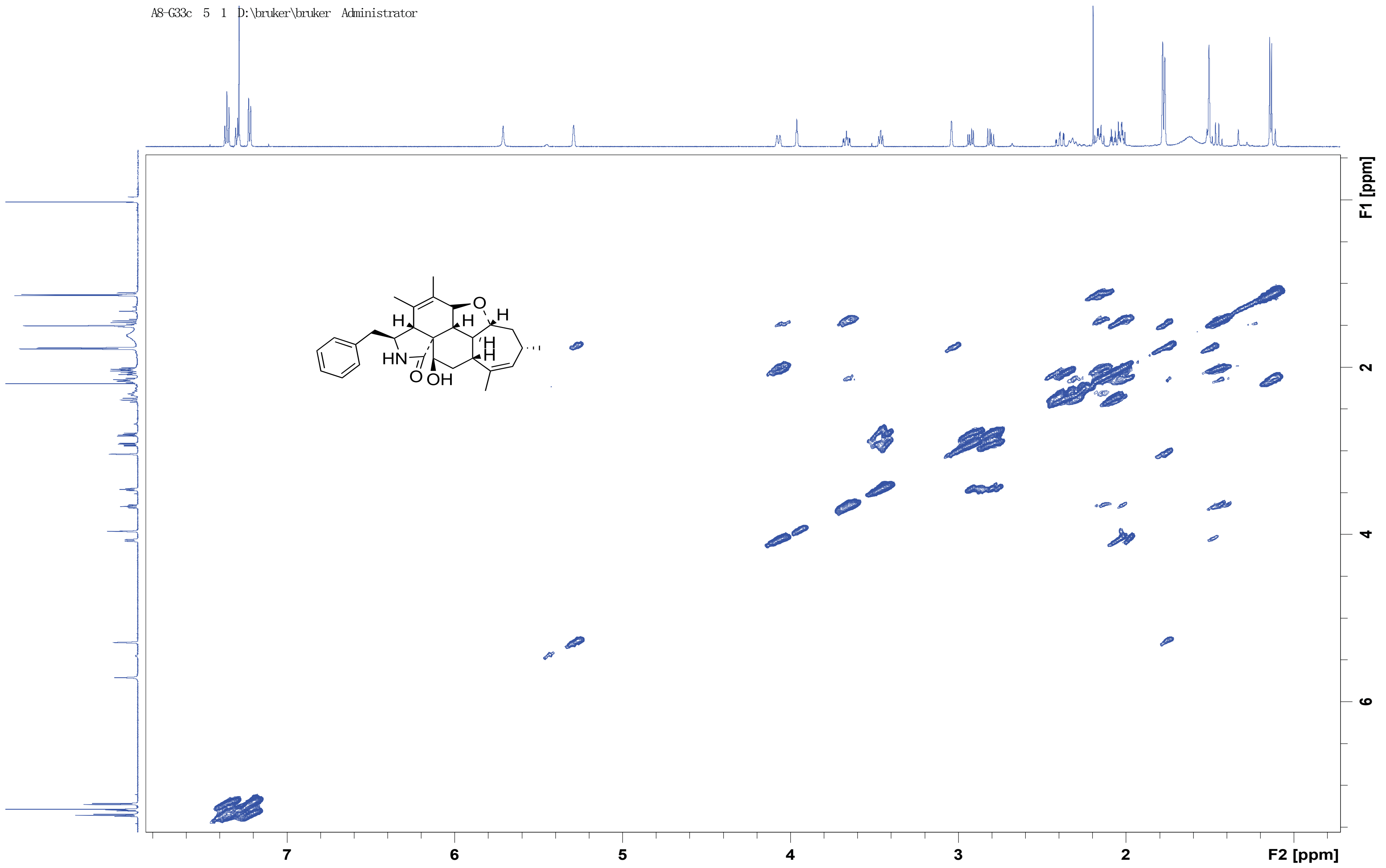

Figure S5. ^{1}H-^{1}H COSY spectrum of phomopchalasin C_1 (**1**)

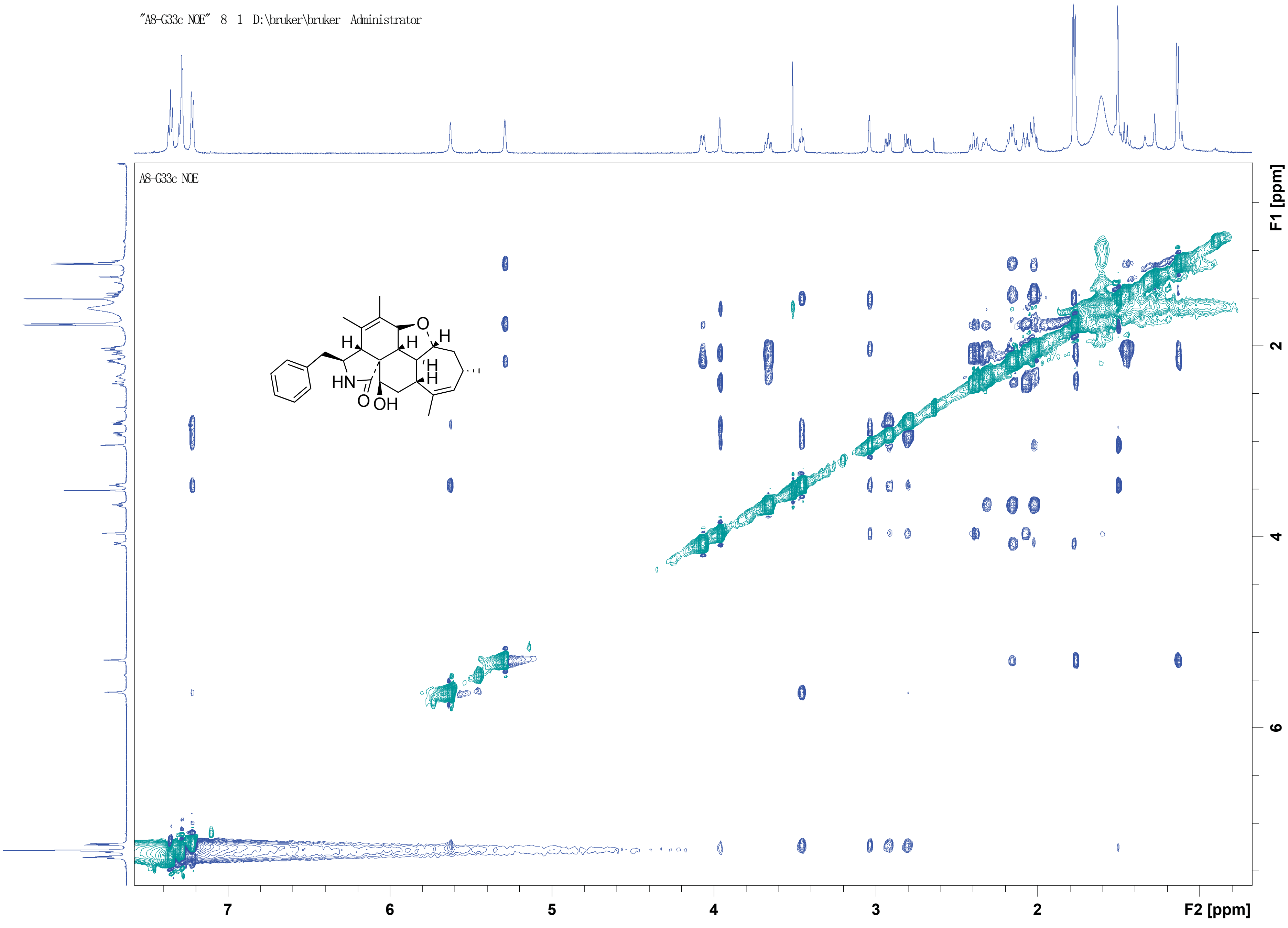

Figure S6. NOE spectrum of phomopchalasin C_1 (**1**)

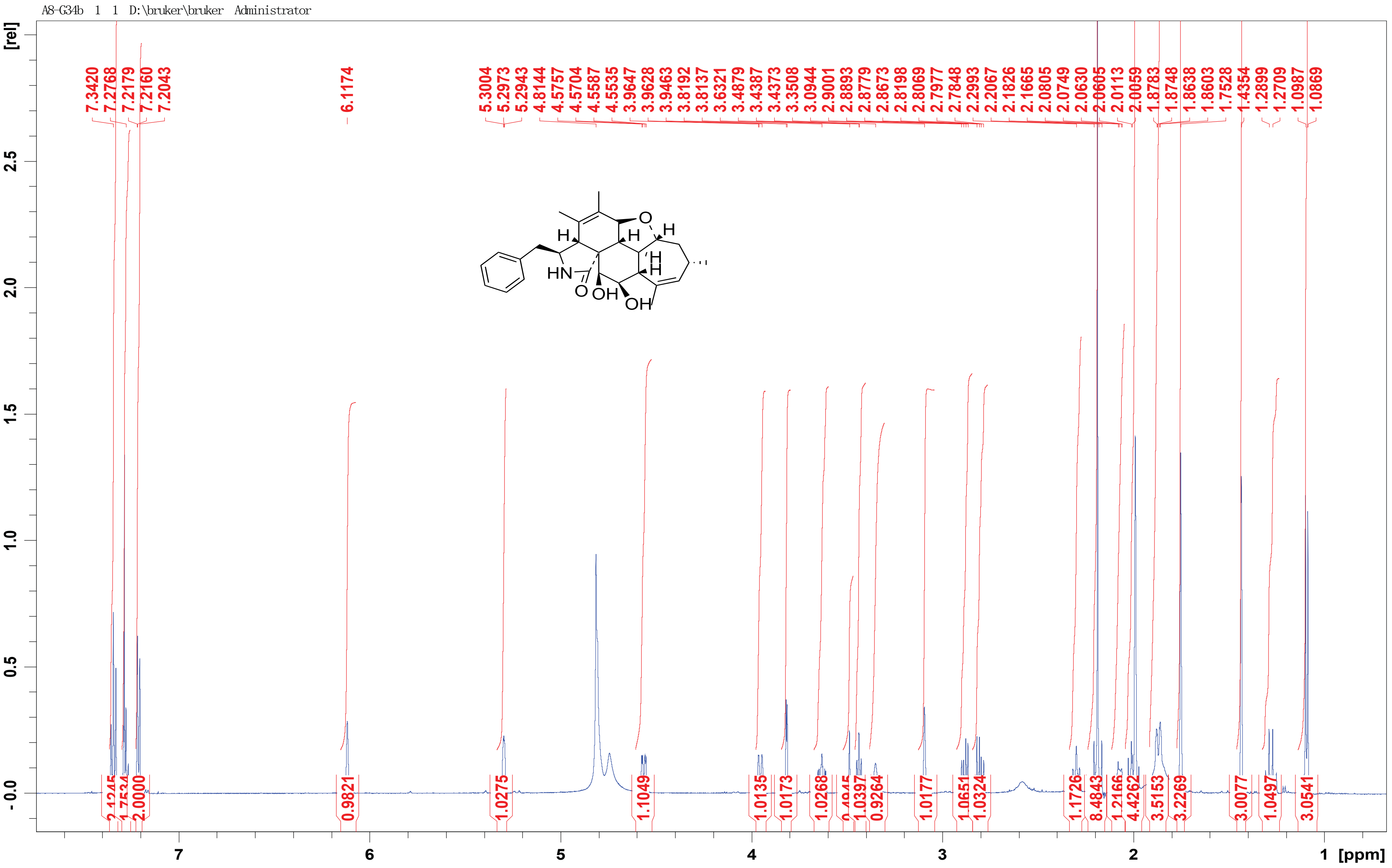

Figure S7. ^{1}H-NMR (600 MHz, $CDCl_3$) spectrum of phomopchalasin C_2 (**2**)

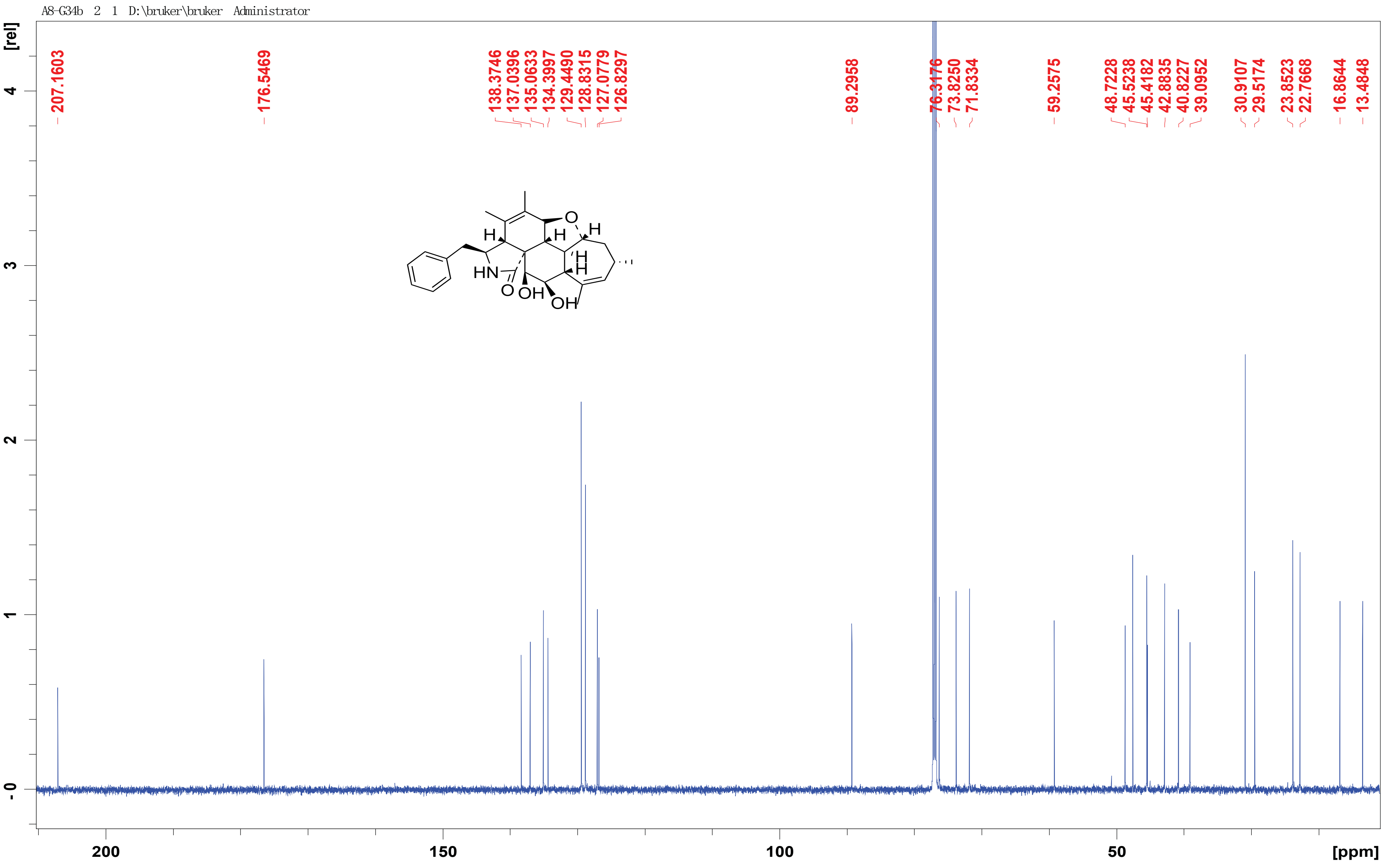

Figure S8. ^{13}C-NMR (150 MHz, $CDCl_3$) spectrum of phomopchalasin C_2 (**2**)

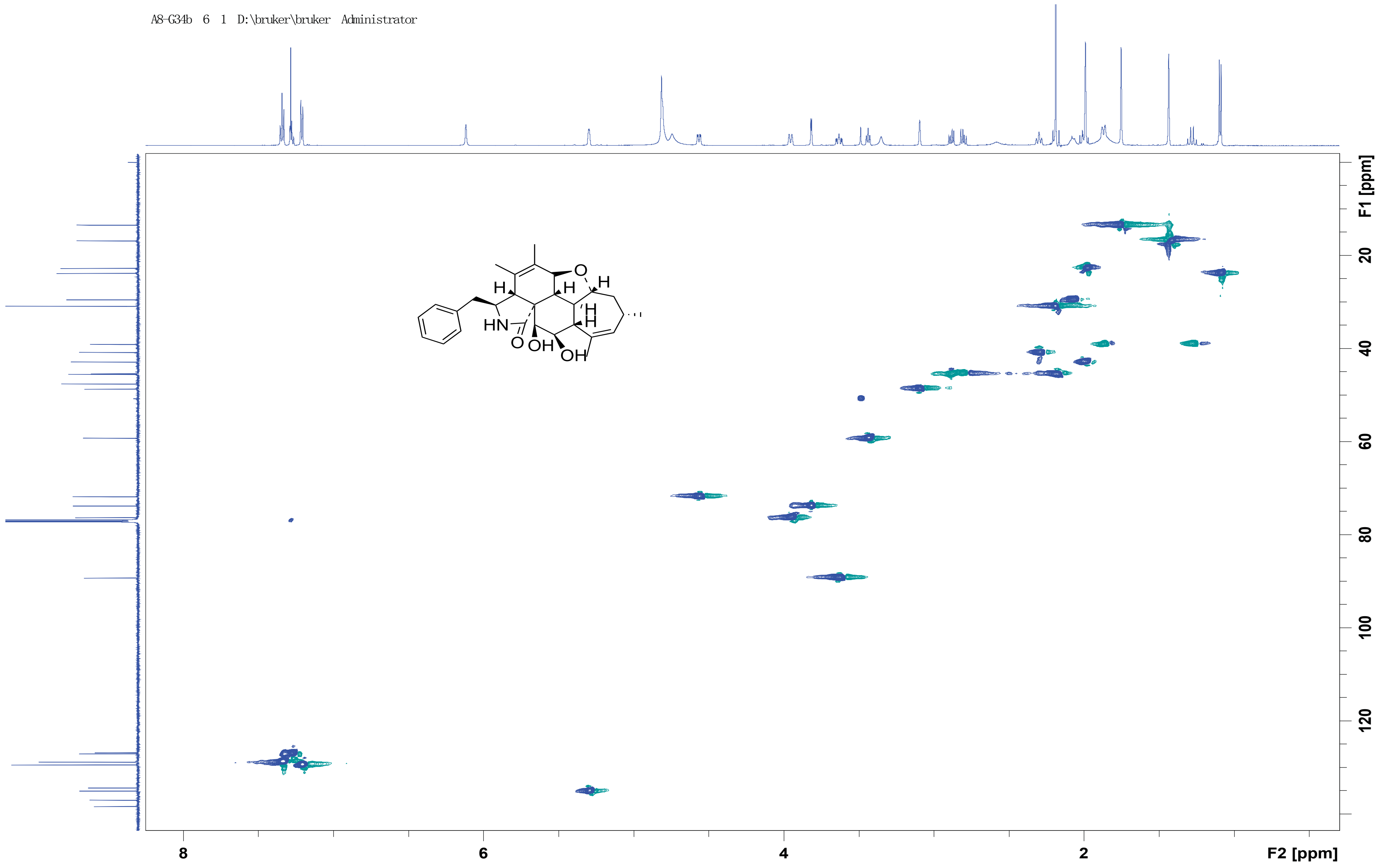

Figure S9. HSQC spectrum of phomopchalasin C_2 (**2**)

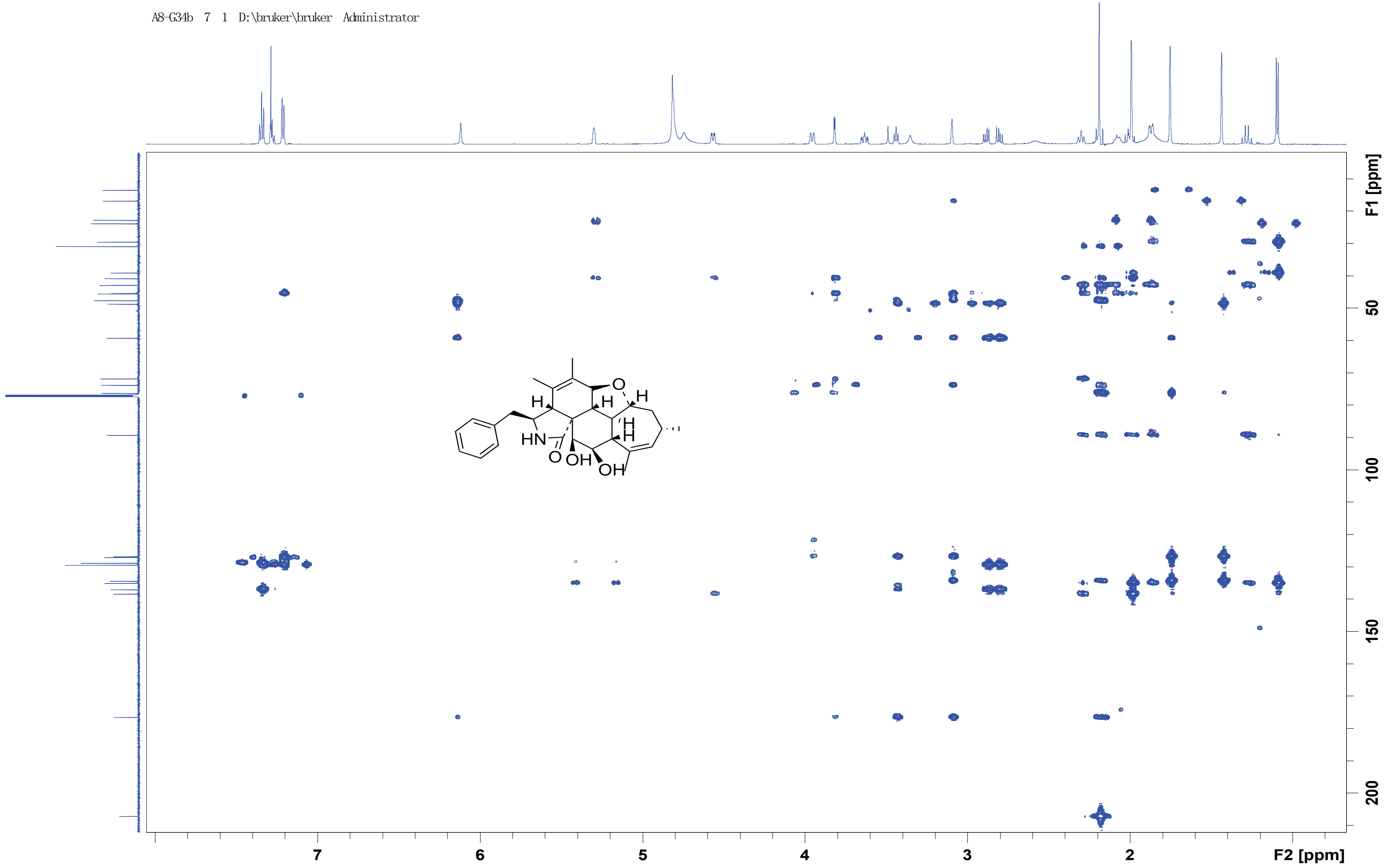

Figure S10. HMBC spectrum of phomopchalasin C_2 (**2**)

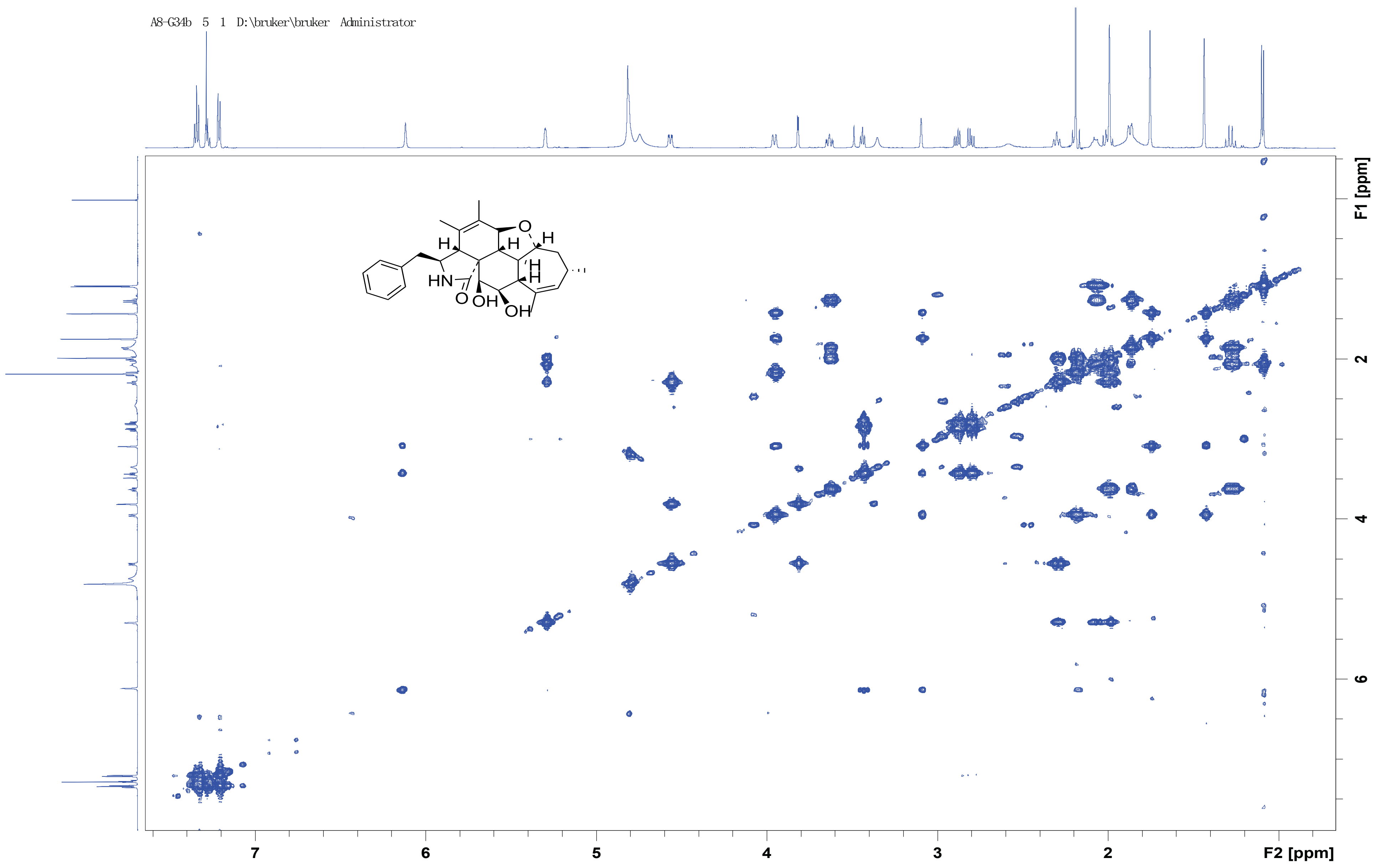

Figure S11. ^{1}H-^{1}H COSY spectrum of phomopchalasin C_2 (**2**)

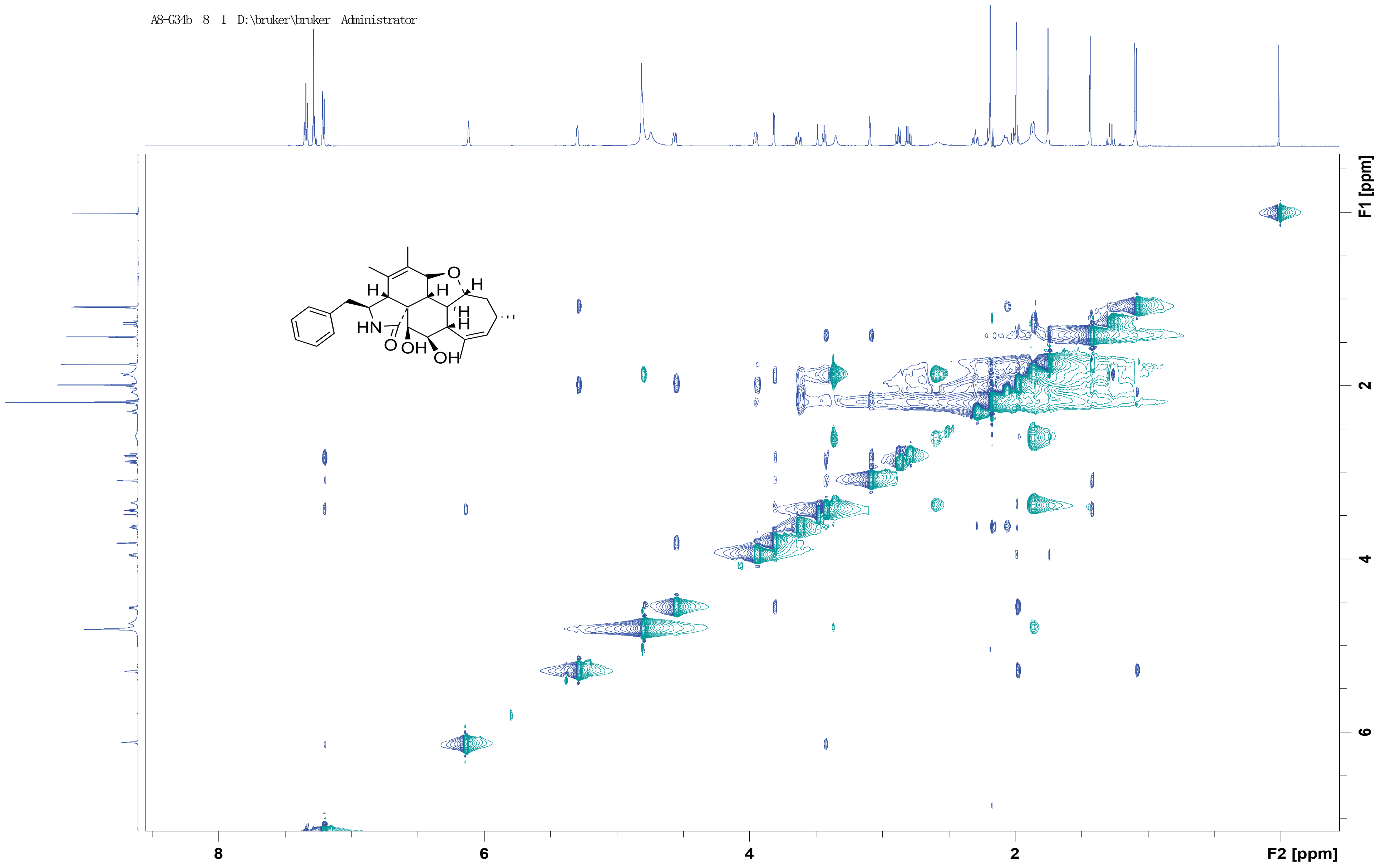

Figure S12. NOE spectrum of phomopchalasin C_2 (**2**)

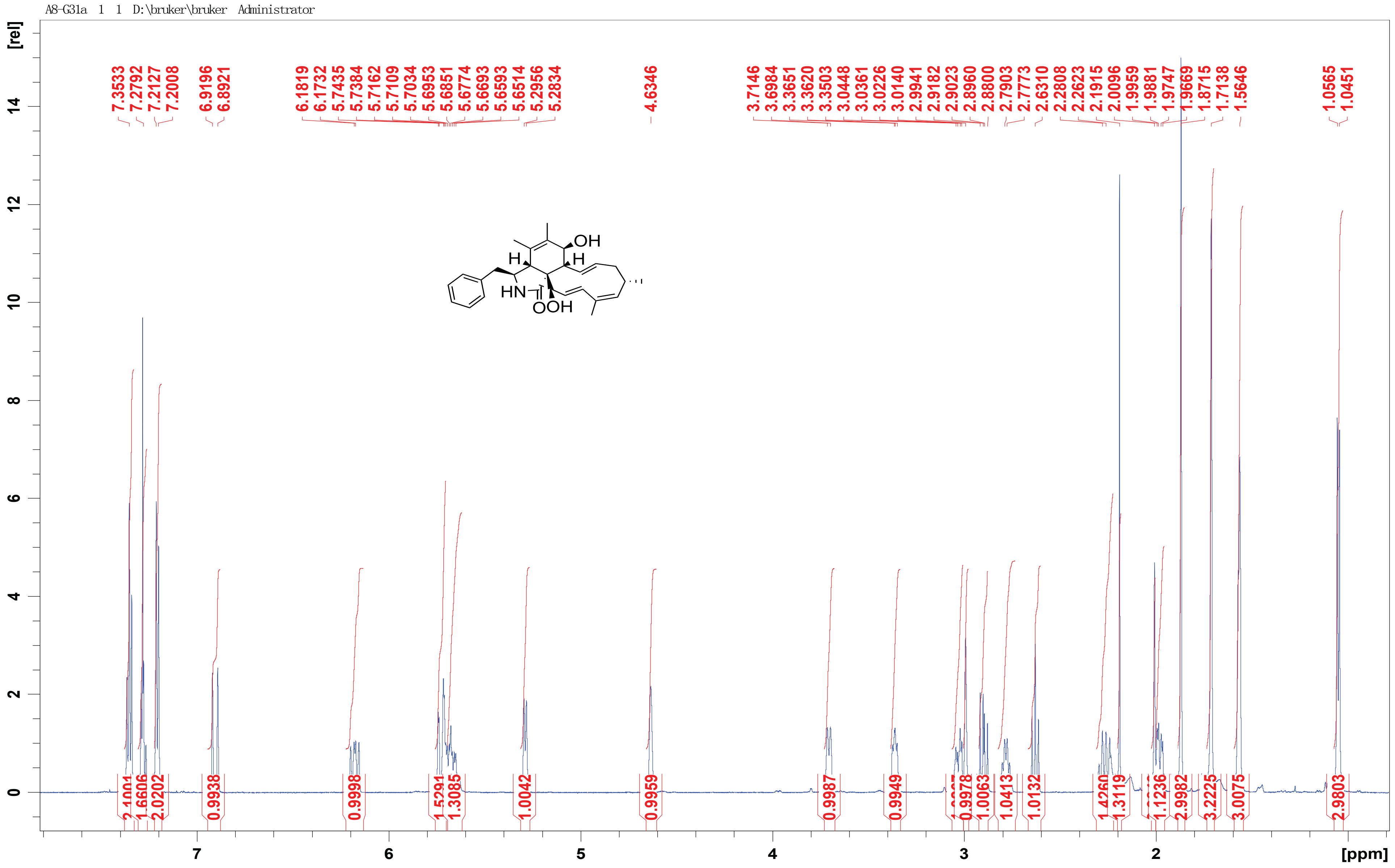

Figure S13. ^{1}H-NMR (600 MHz, $CDCl_3$) spectrum of phomopchalasin C_3 (**3**)

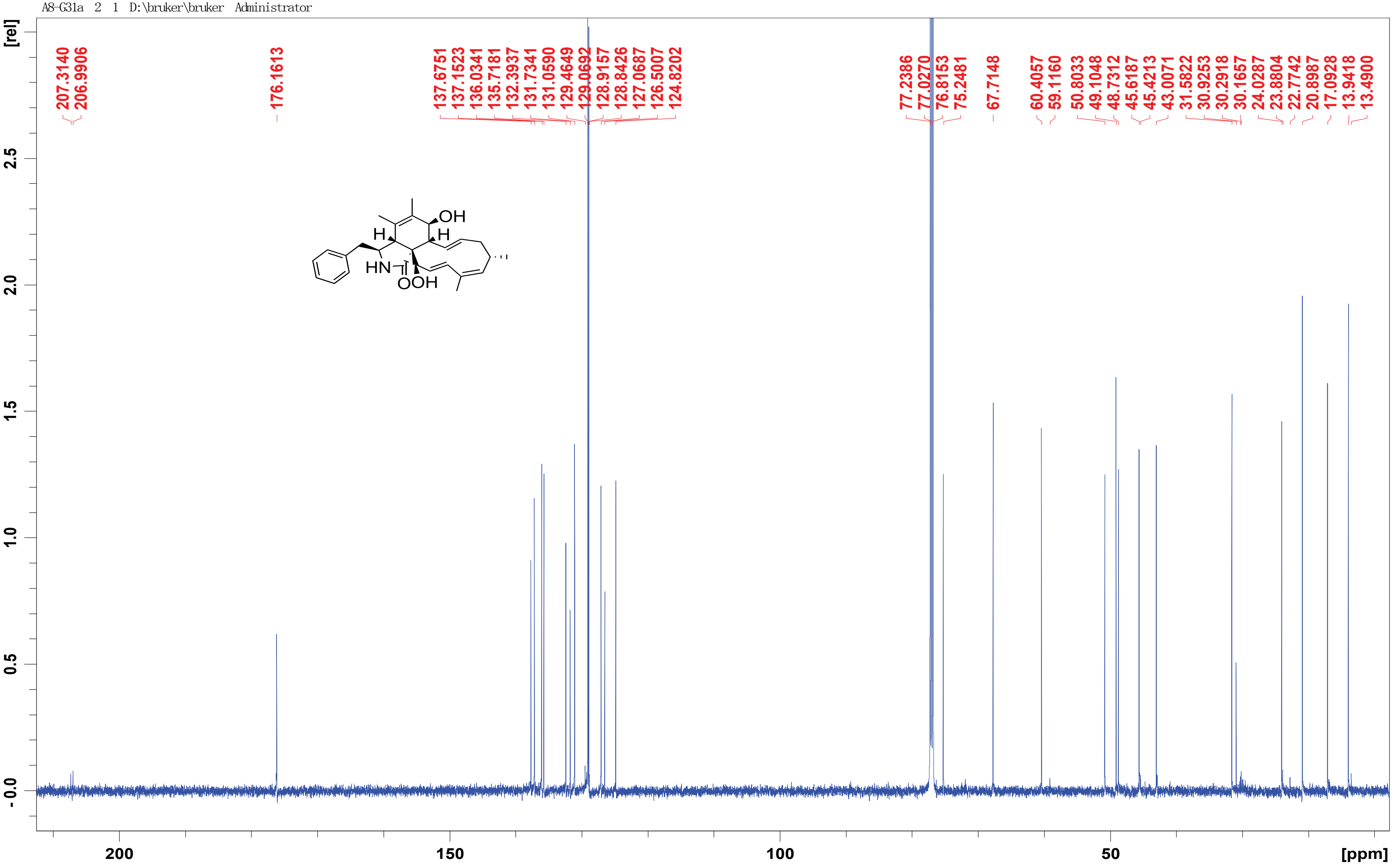

Figure S14. ^{13}C-NMR (150 MHz, $CDCl_3$) spectrum of phomopchalasin C_3 (**3**)

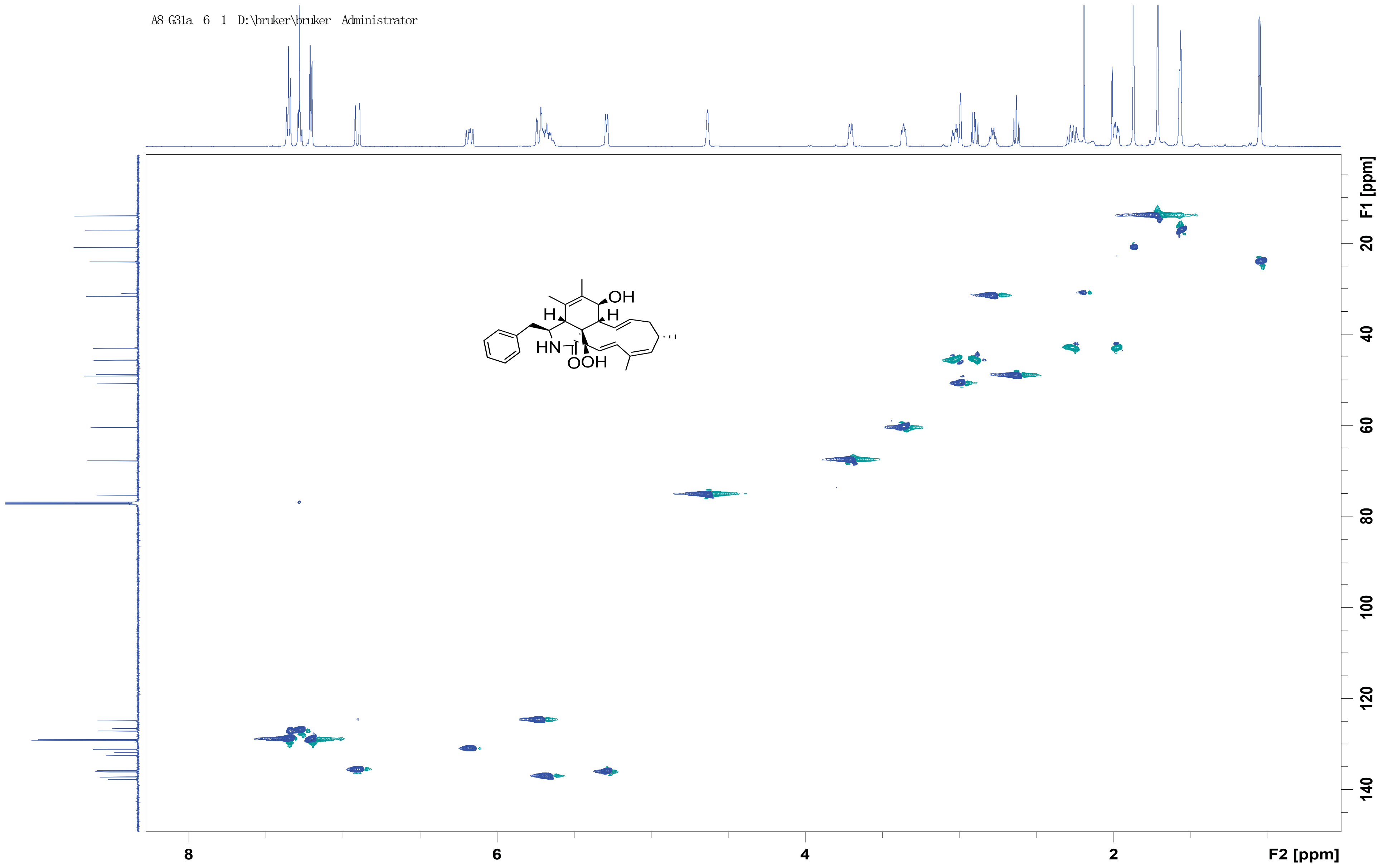

Figure S15. HSQC spectrum of phomopchalasin C_3 (**3**)

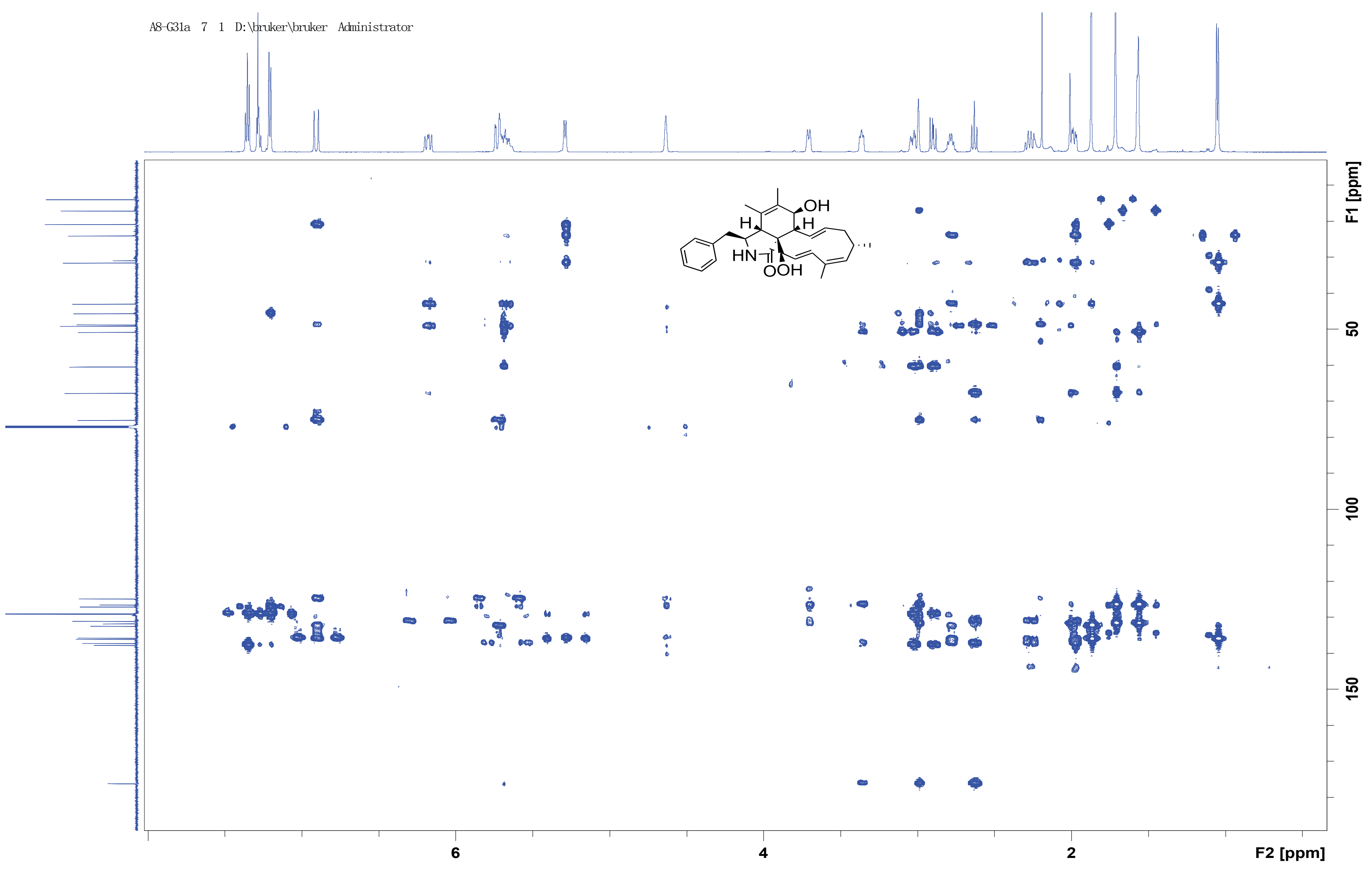

Figure S16. HMBC spectrum of phomopchalasin C_3 (**3**)

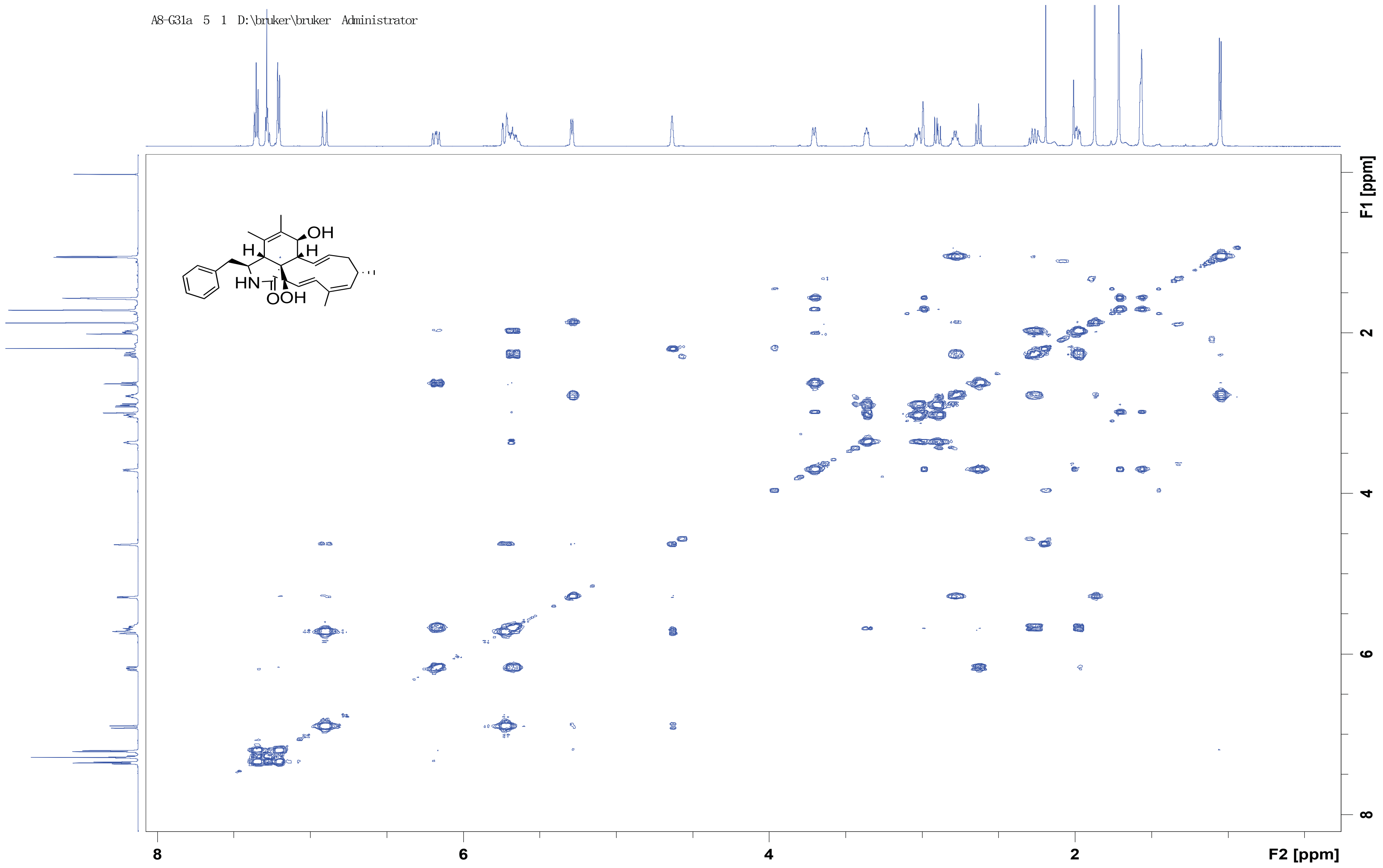

Figure S17. ^{1}H-^{1}H COSY spectrum of phomopchalasin C_3 (**3**)

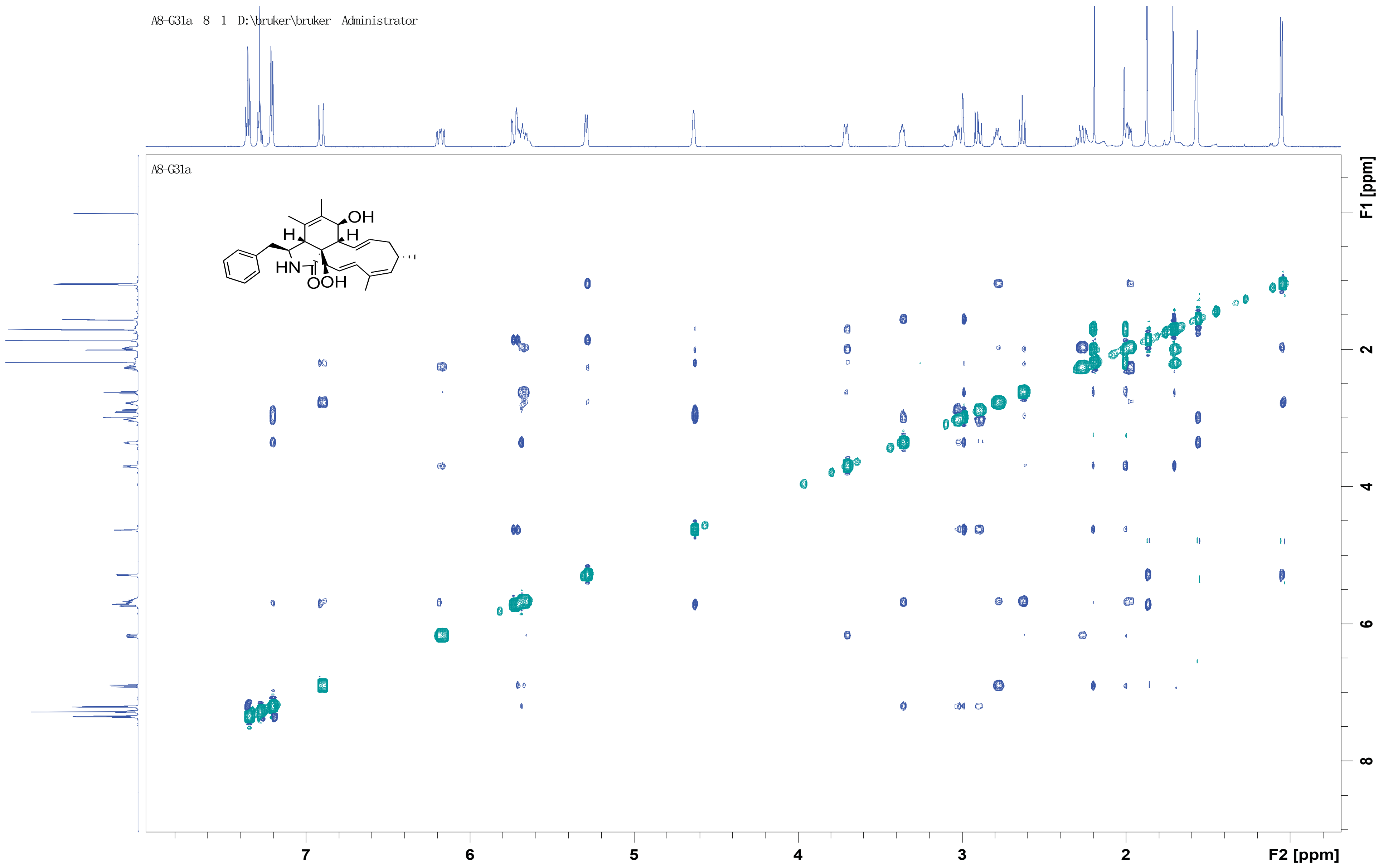

Figure S18. NOE spectrum of phomopchalasin C_3 (**3**)

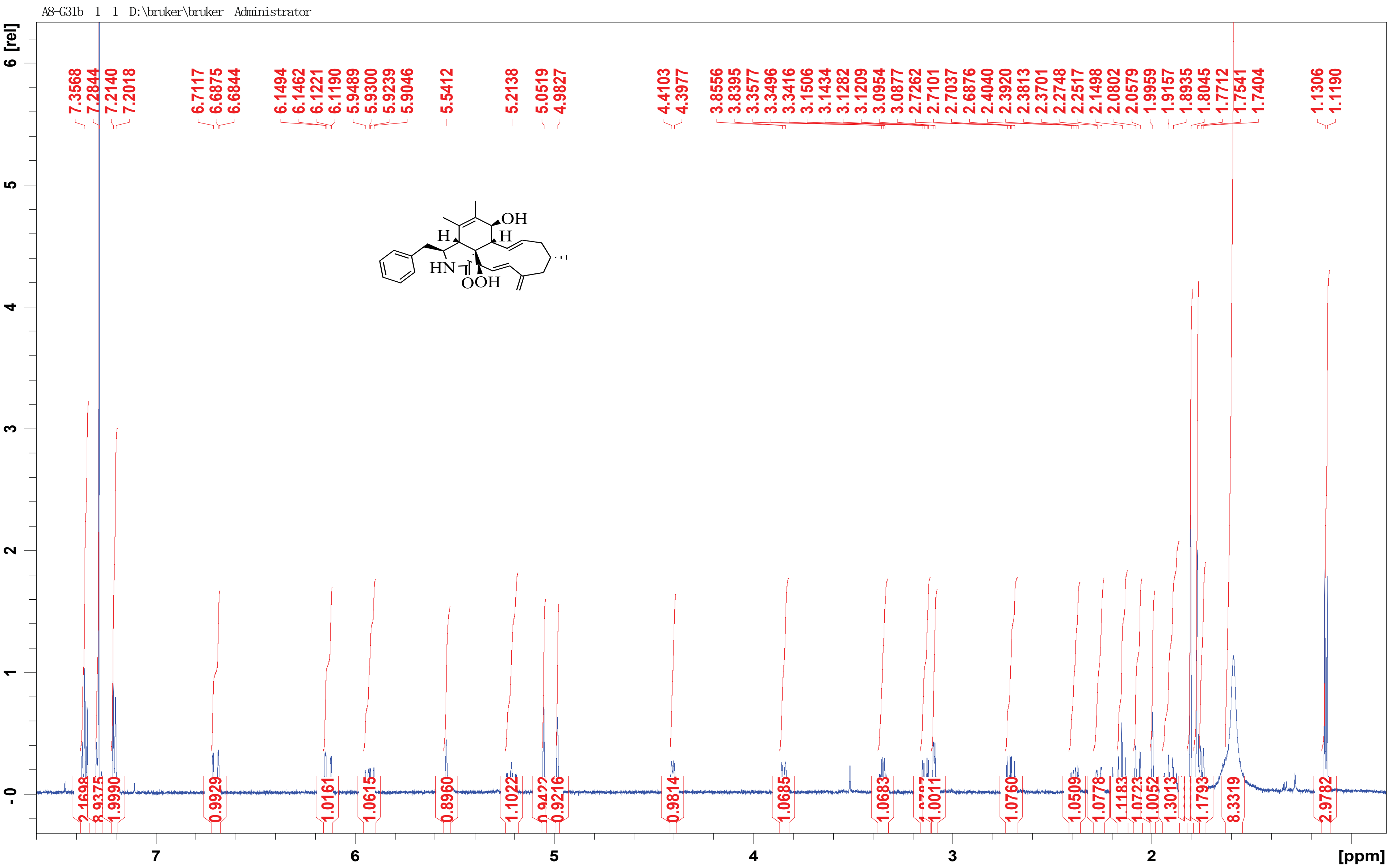

Figure S19. ^{1}H-NMR (600 MHz, $CDCl_3$) spectrum of phomopchalasin C_4 (**4**)

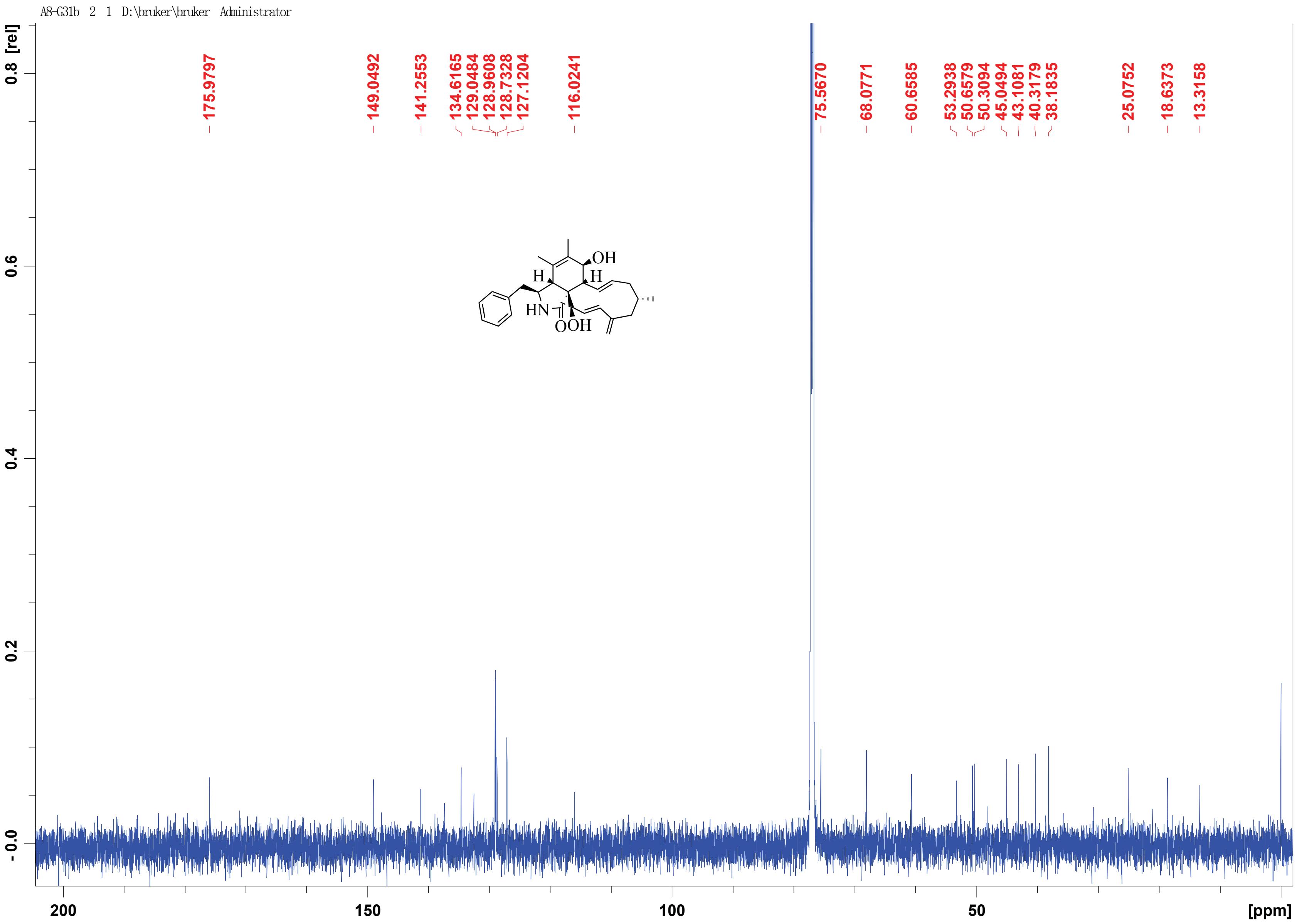

Figure S20. ^{13}C-NMR (150 MHz, $CDCl_3$) spectrum of phomopchalasin C_4 (**4**)

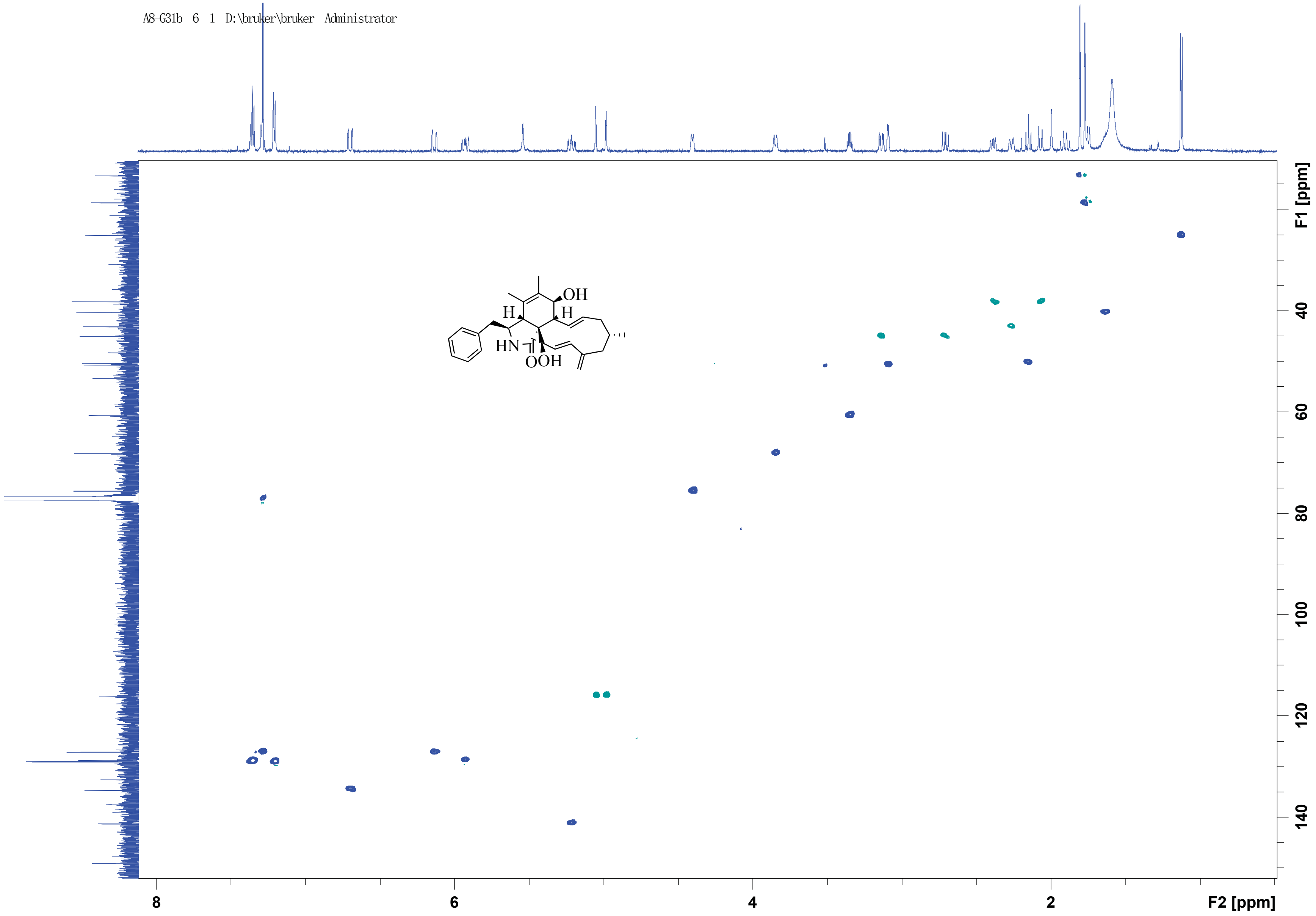

Figure S21. HSQC spectrum of phomopchalasin C_4 (**4**)

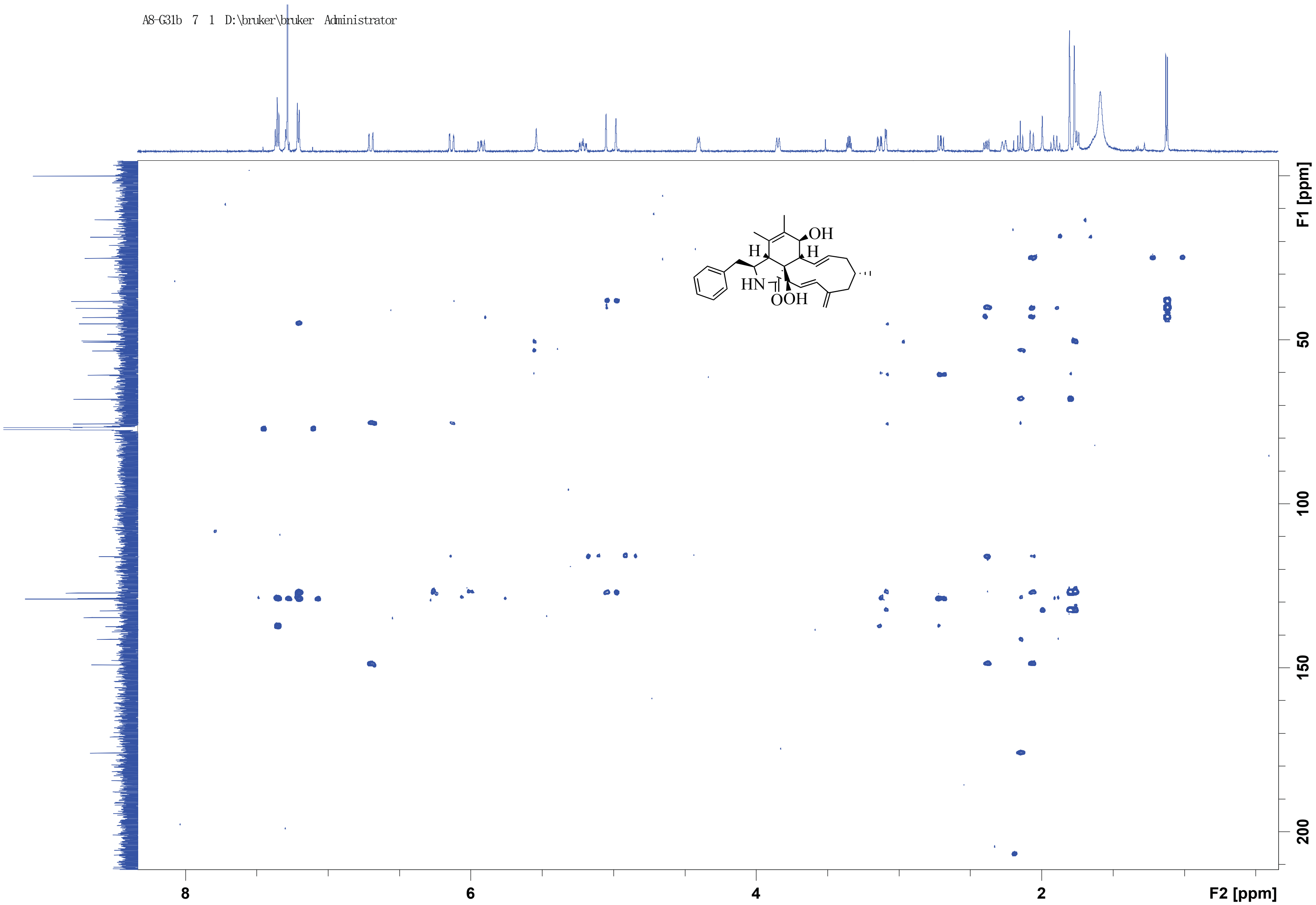

Figure S22. HMBC spectrum of phomopchalasin C_4 (**4**)

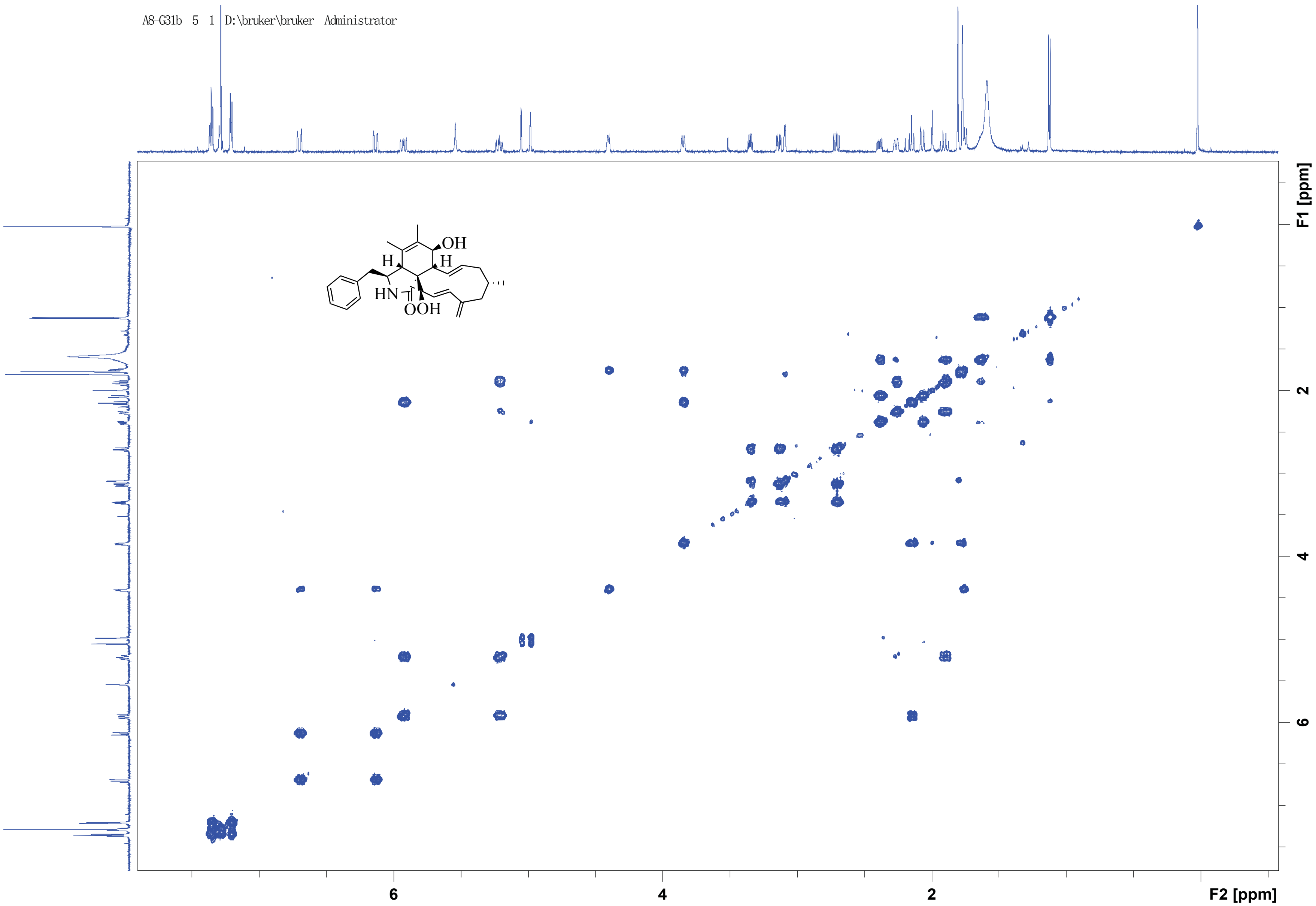

Figure S23. ^{1}H-^{1}H COSY spectrum of phomopchalasin C_4 (**4**)

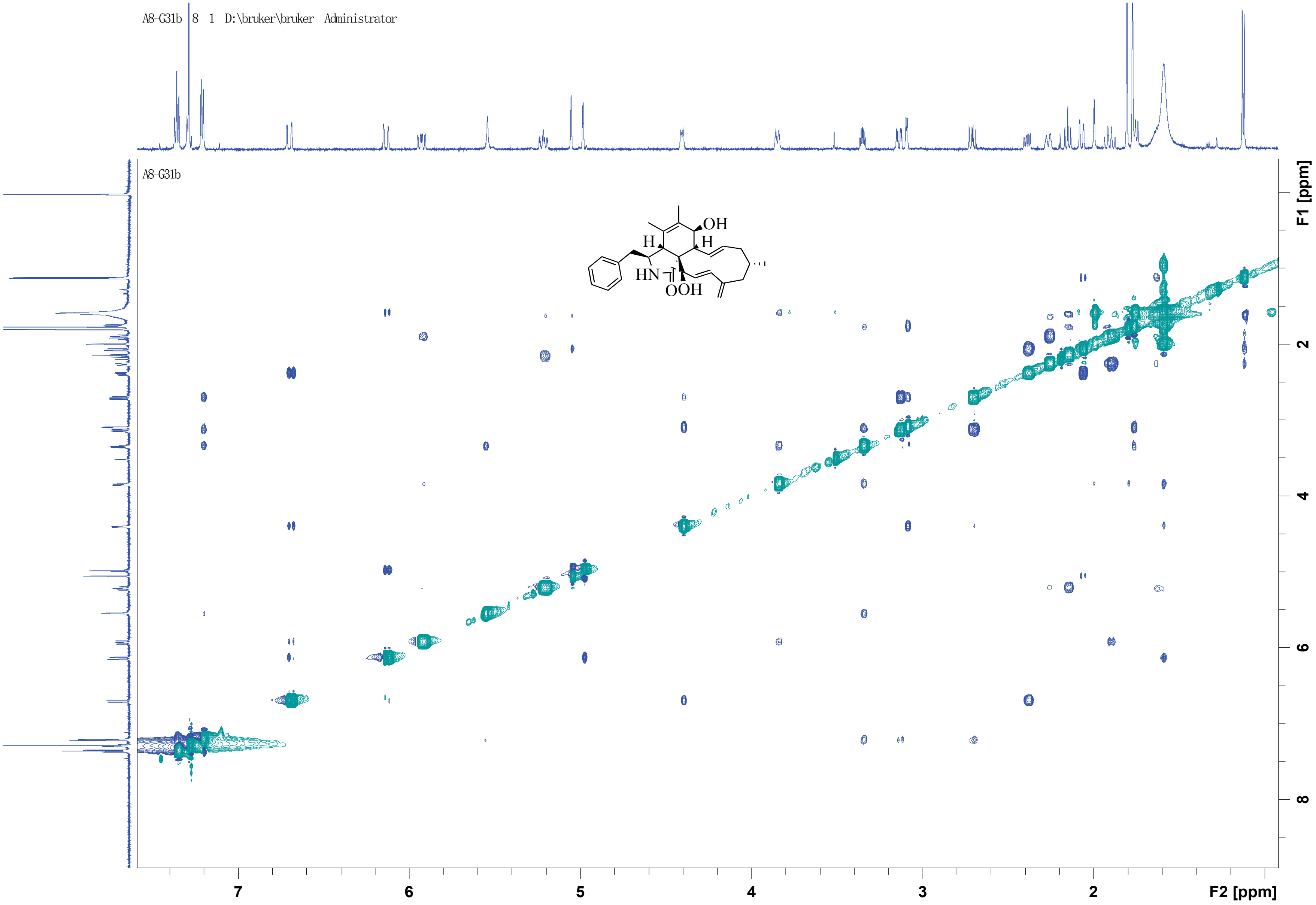

Figure S24. NOE spectrum of phomopchalasin C_4 (**4**)

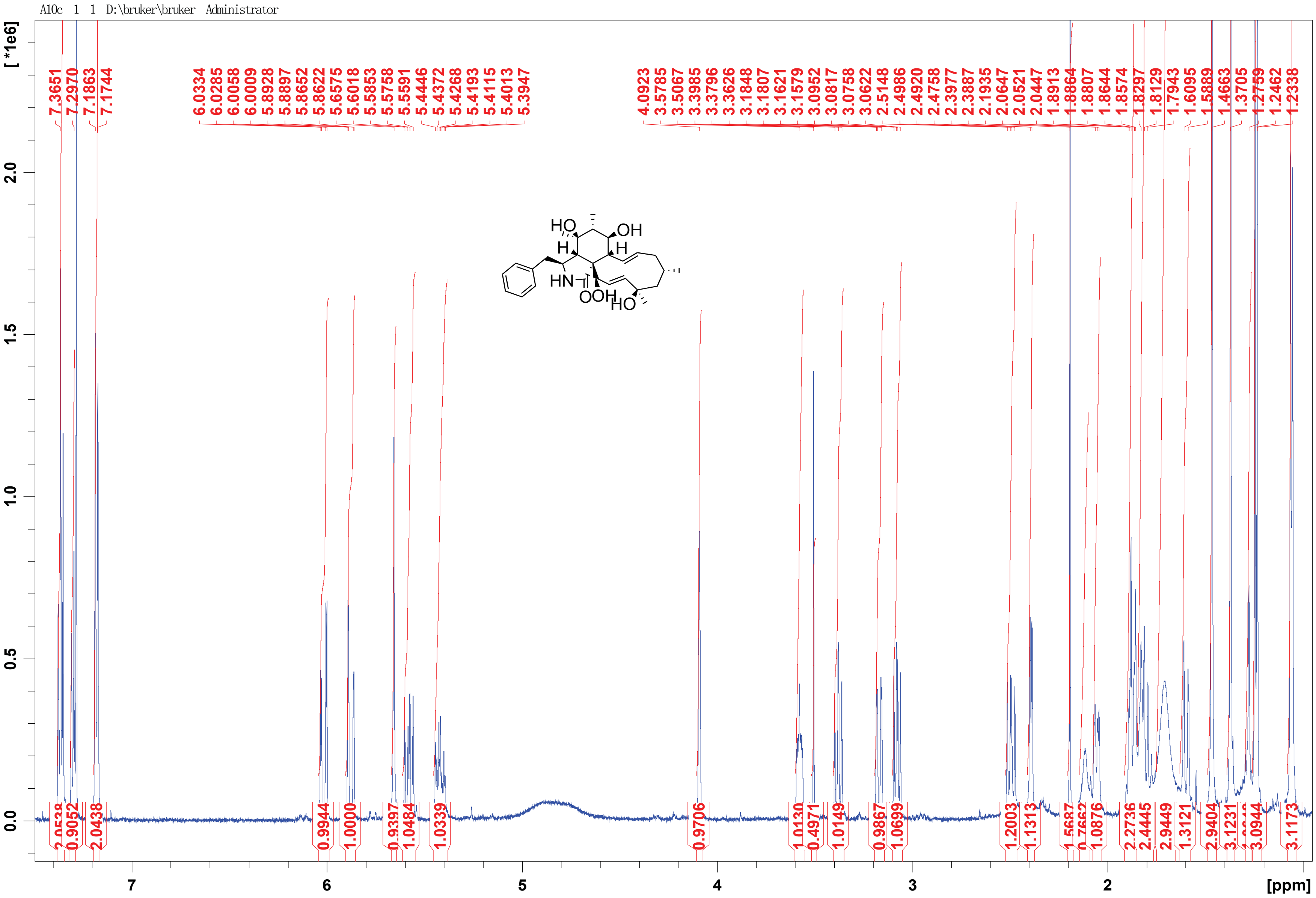

Figure S25. ^{1}H-NMR (600 MHz, $CDCl_3$) spectrum of phomopchalasin C_5 (**5**)

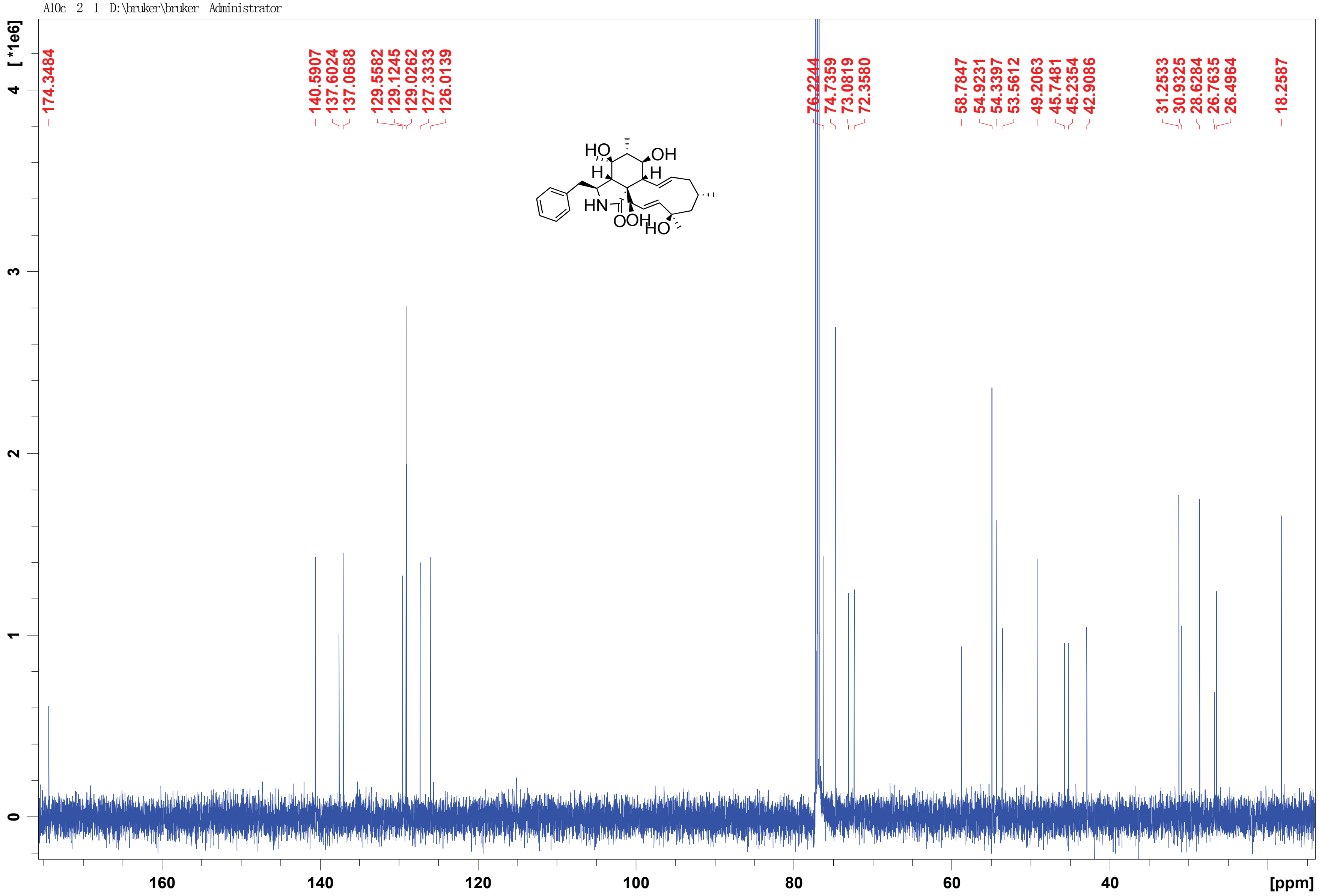

Figure S26. ^{13}C-NMR (150 MHz, $CDCl_3$) spectrum of phomopchalasin C_5 (**5**)

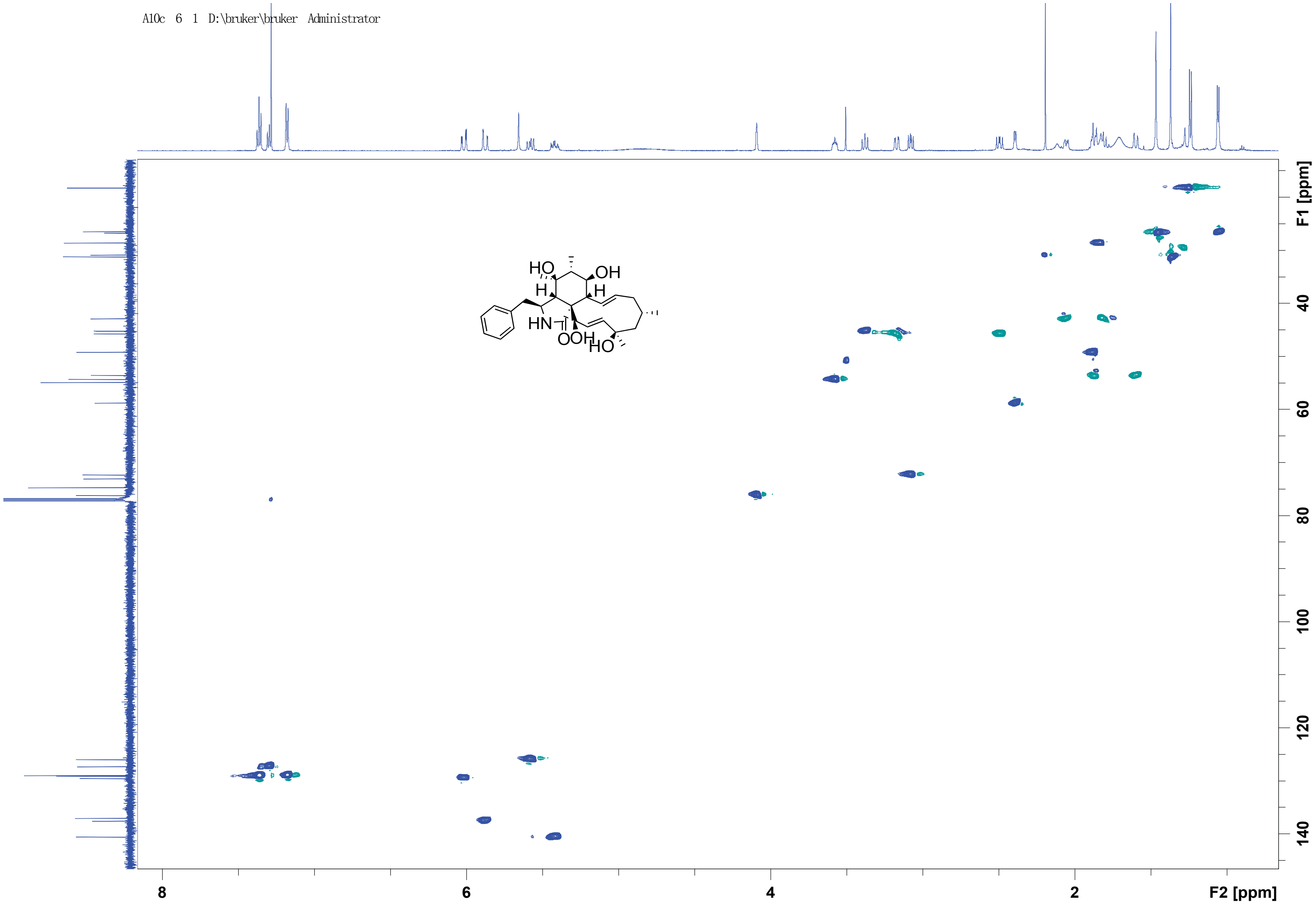

Figure S27. HSQC spectrum of phomopchalasin C_5 (**5**)

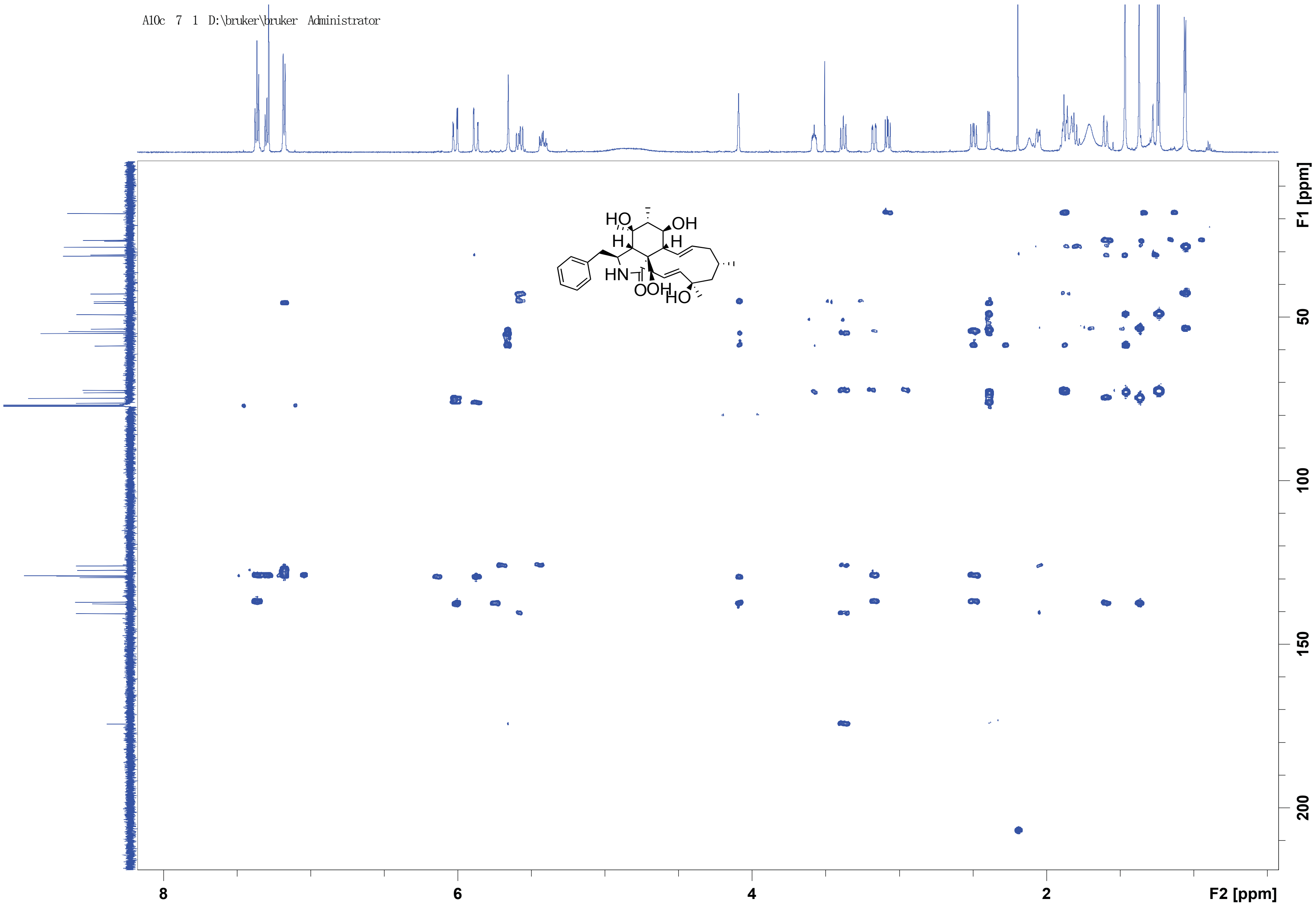

Figure S28. HMBC spectrum of phomopchalasin C_5 (**5**)

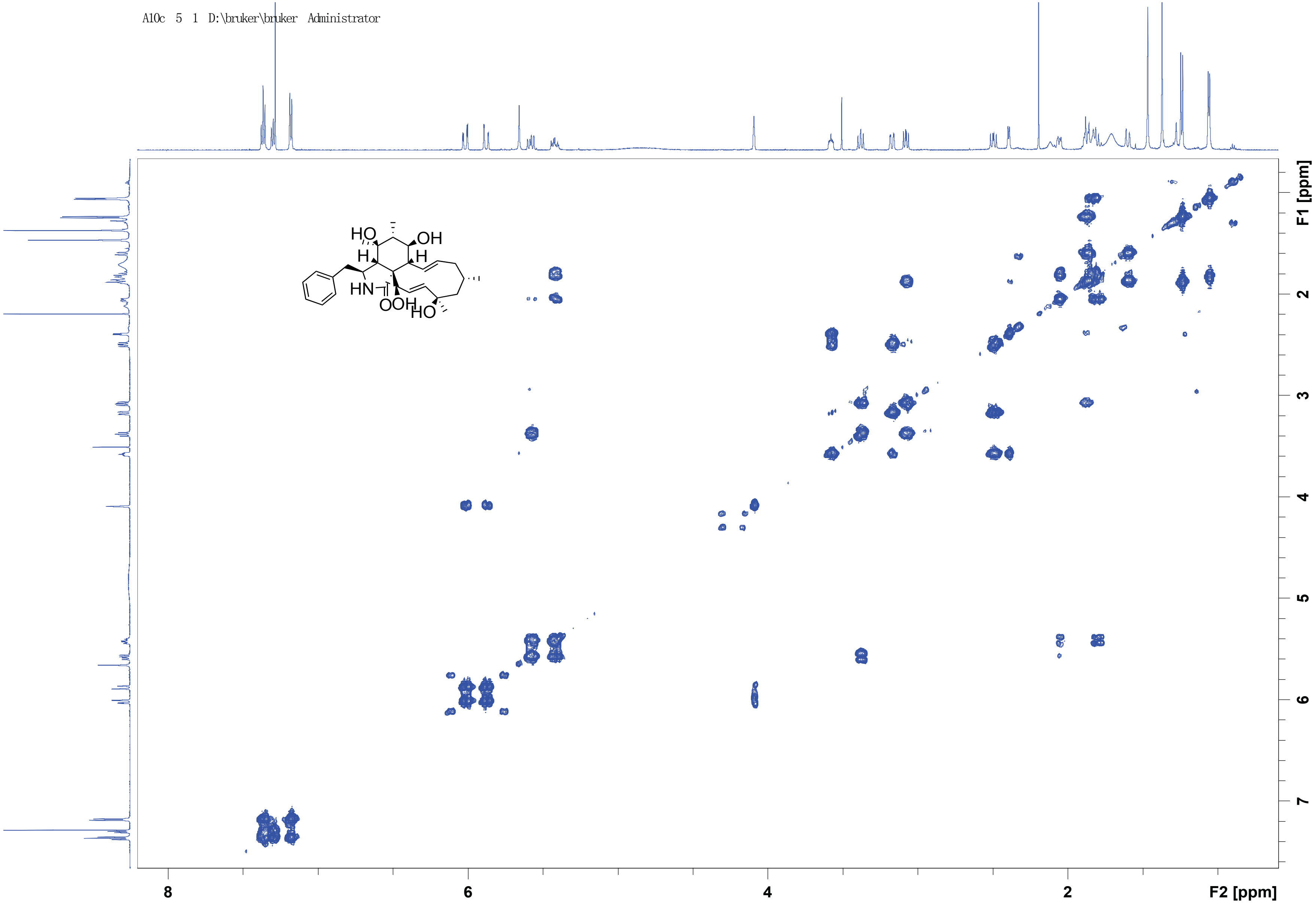

Figure S29. ^{1}H-^{1}H COSY spectrum of phomopchalasin C_5 (**5**)

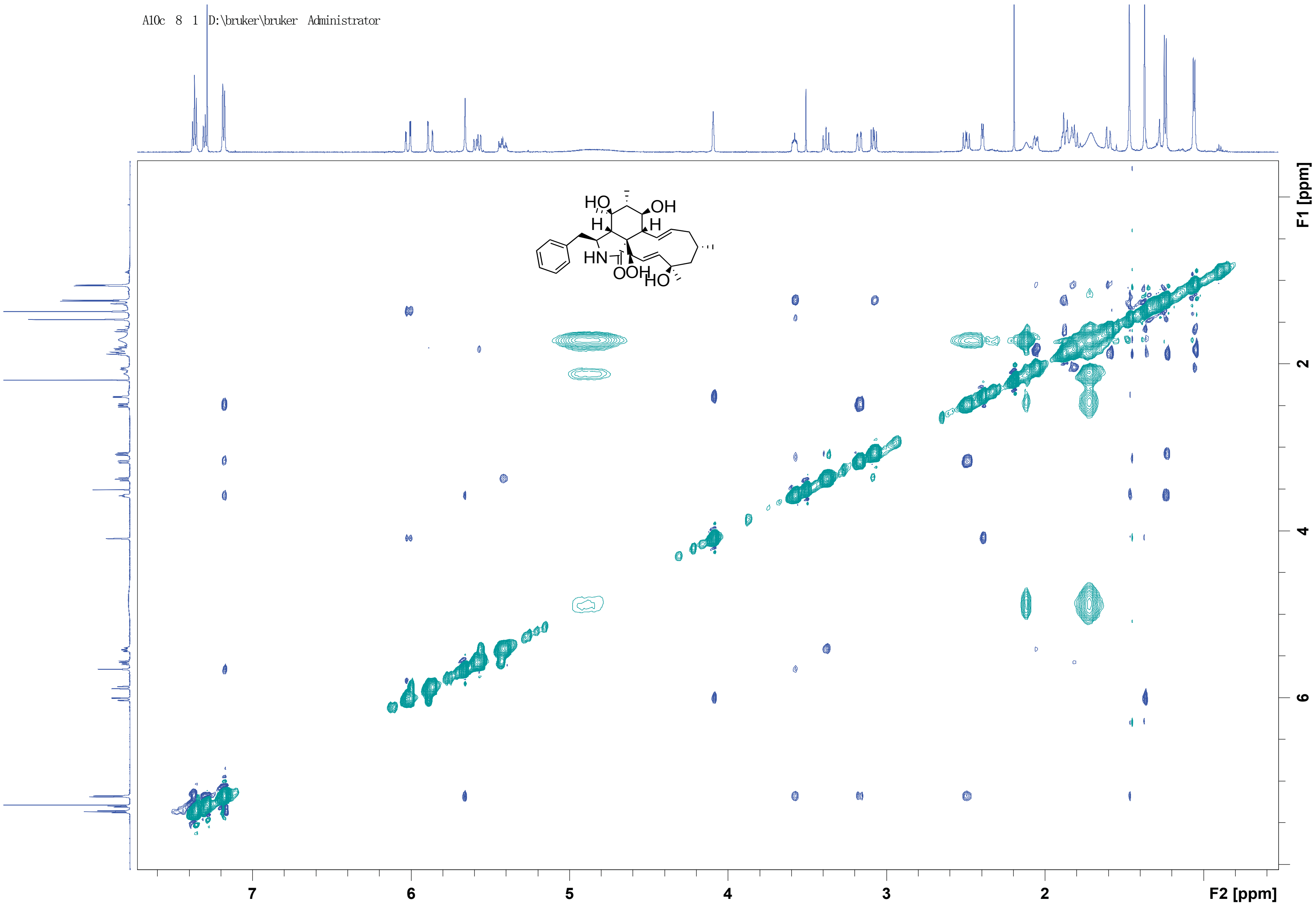

Figure S30. NOE spectrum of phomopchalasin C_5 (**5**)

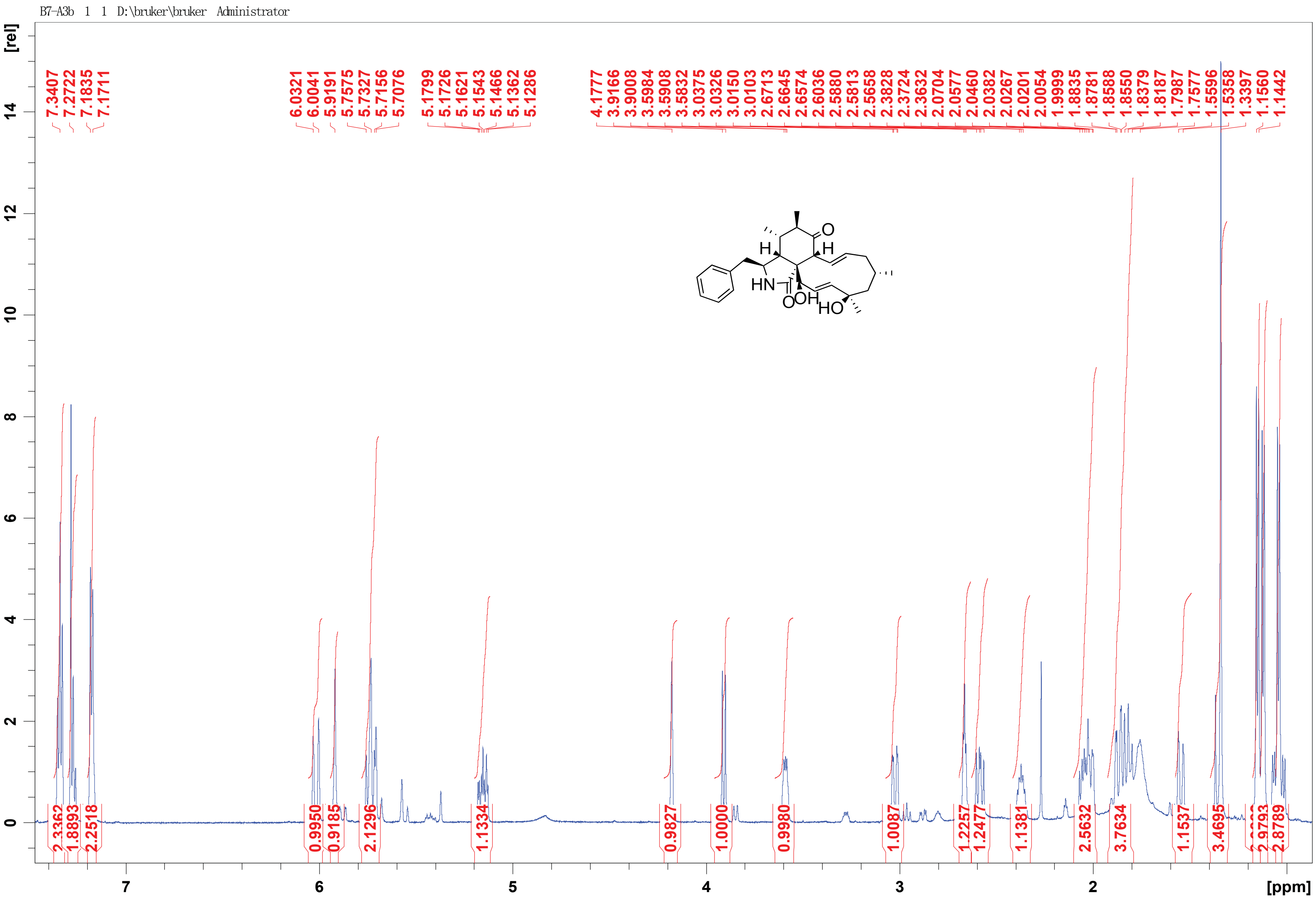

Figure S31. ^{1}H-NMR (600 MHz, $CDCl_3$) spectrum of phomopchalasin C_6 (**6**)

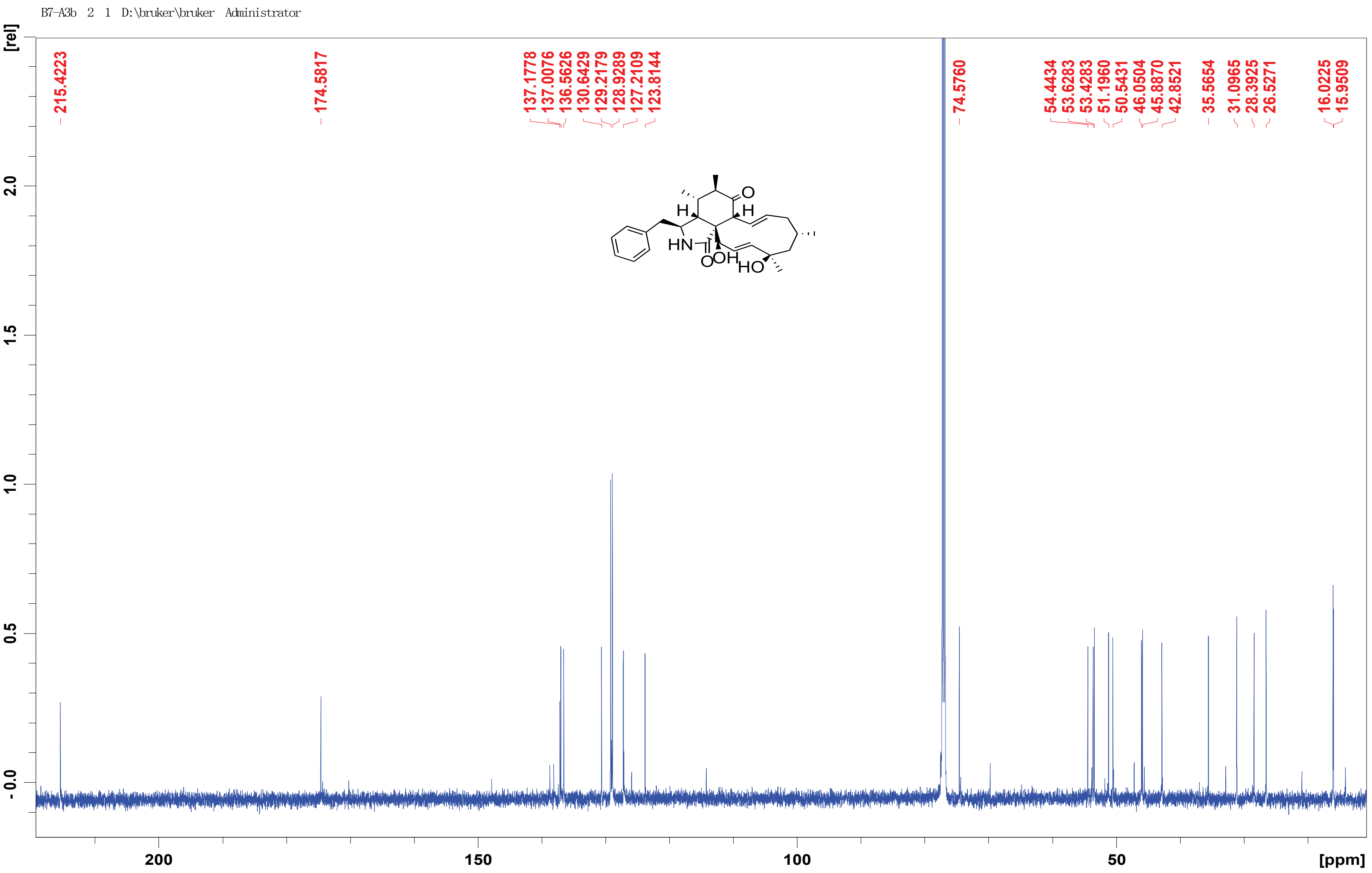

Figure S32. ^{13}C-NMR (150 MHz, $CDCl_3$) spectrum of phomopchalasin C_6 (**6**)

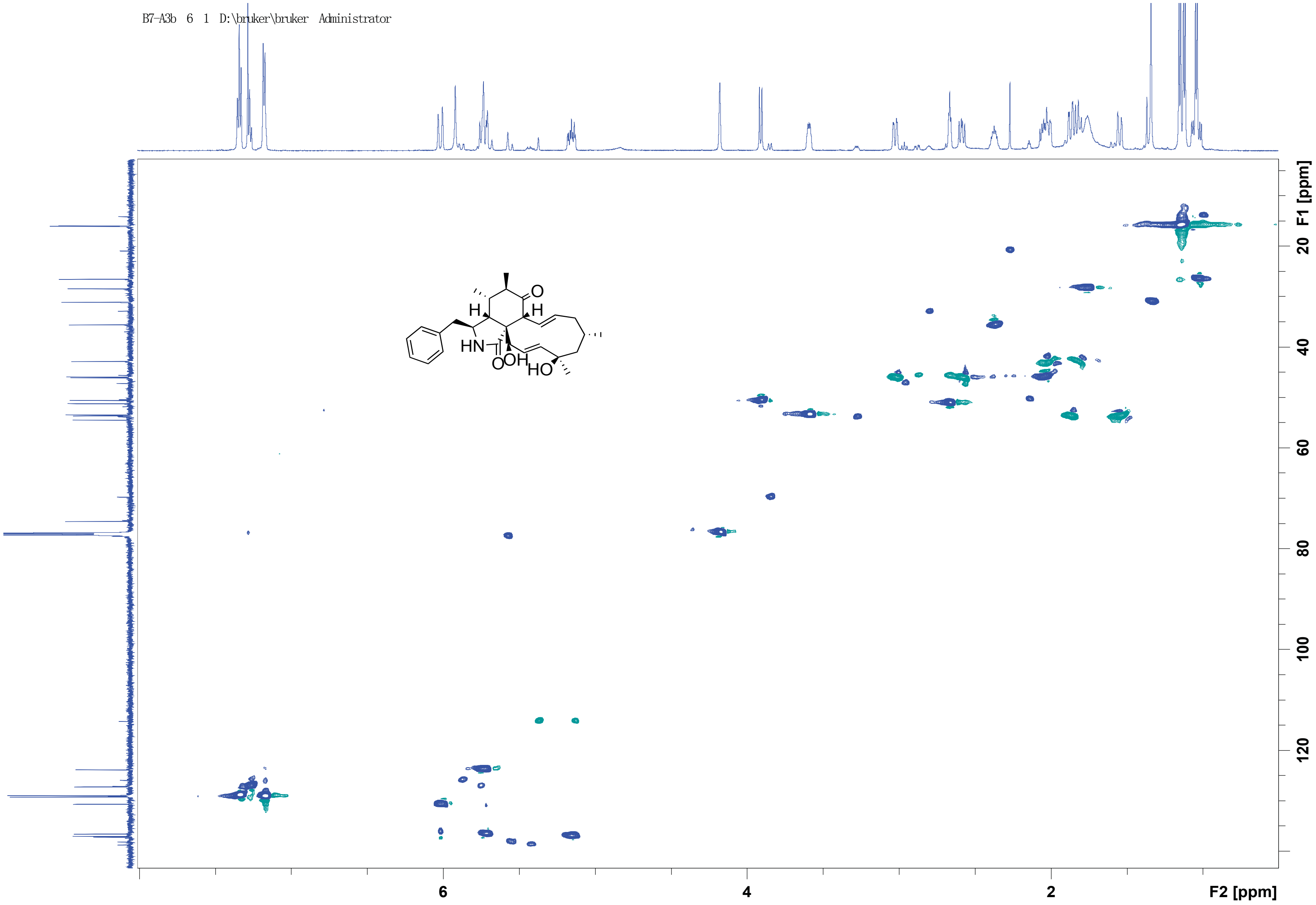

Figure S33. HSQC spectrum of phomopchalasin C_6 (**6**)

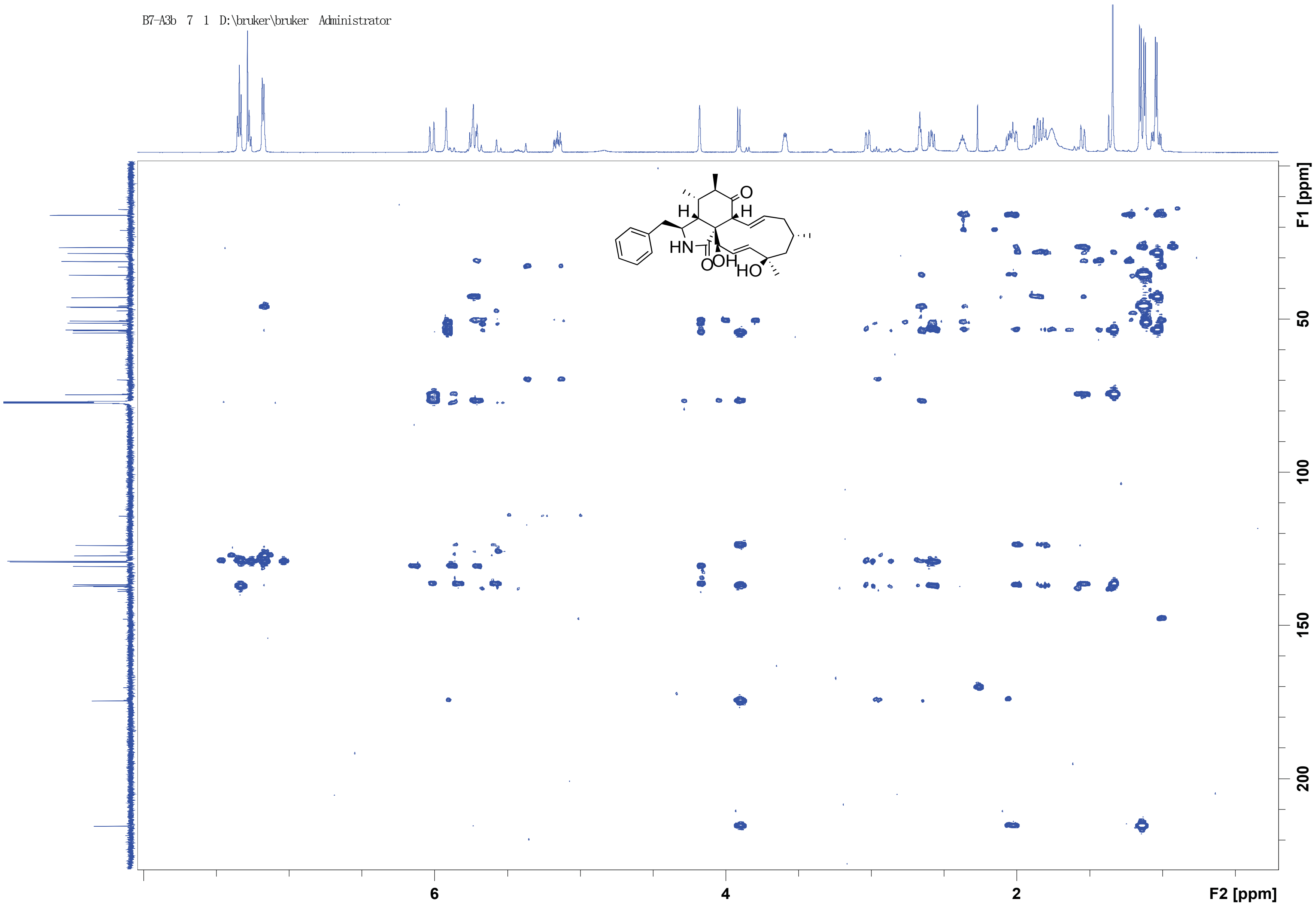

Figure S34. HMBC spectrum of phomopchalasin C_6 (**6**)

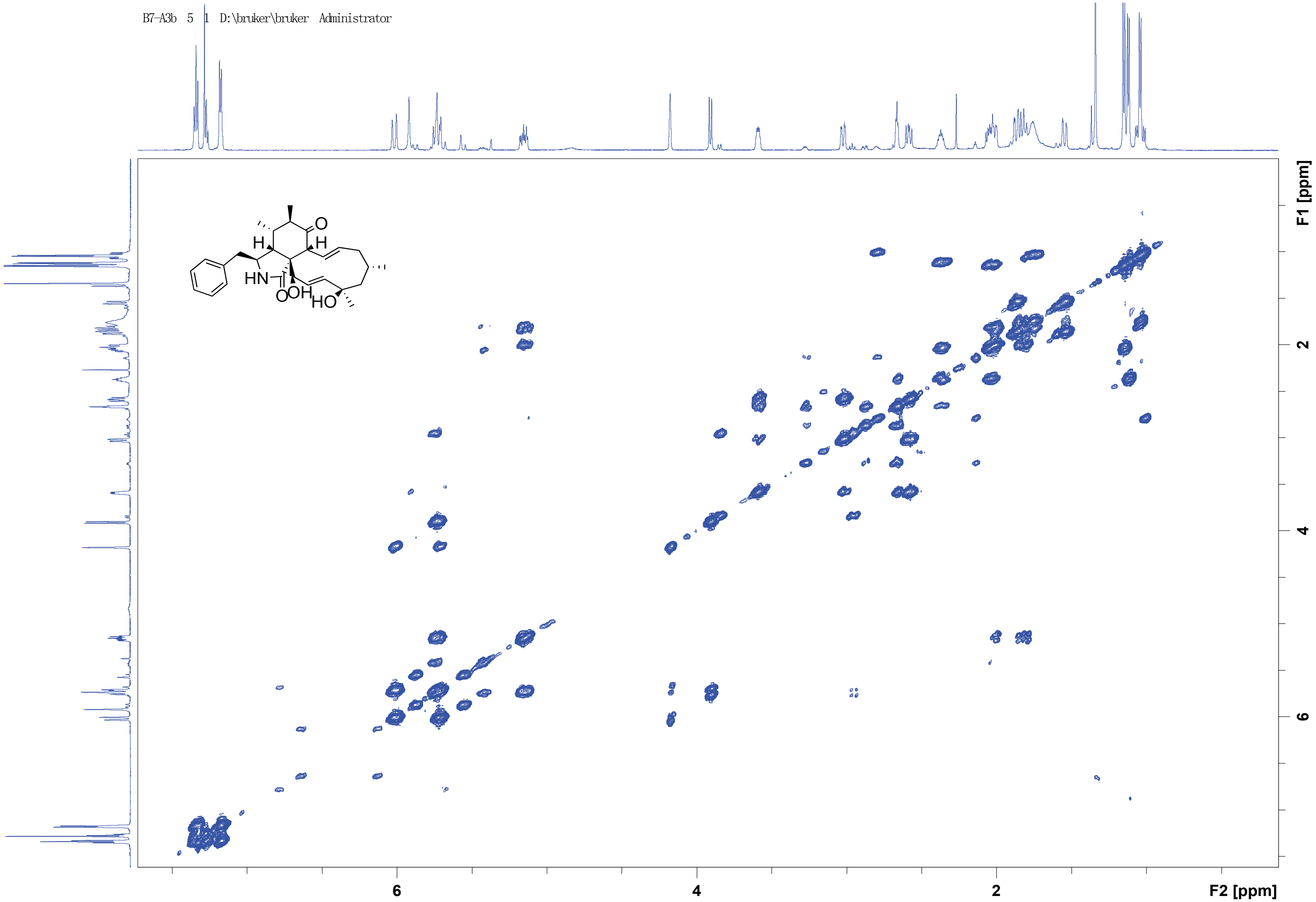

Figure S35. ^{1}H-^{1}H COSY spectrum of phomopchalasin C_6 (**6**)

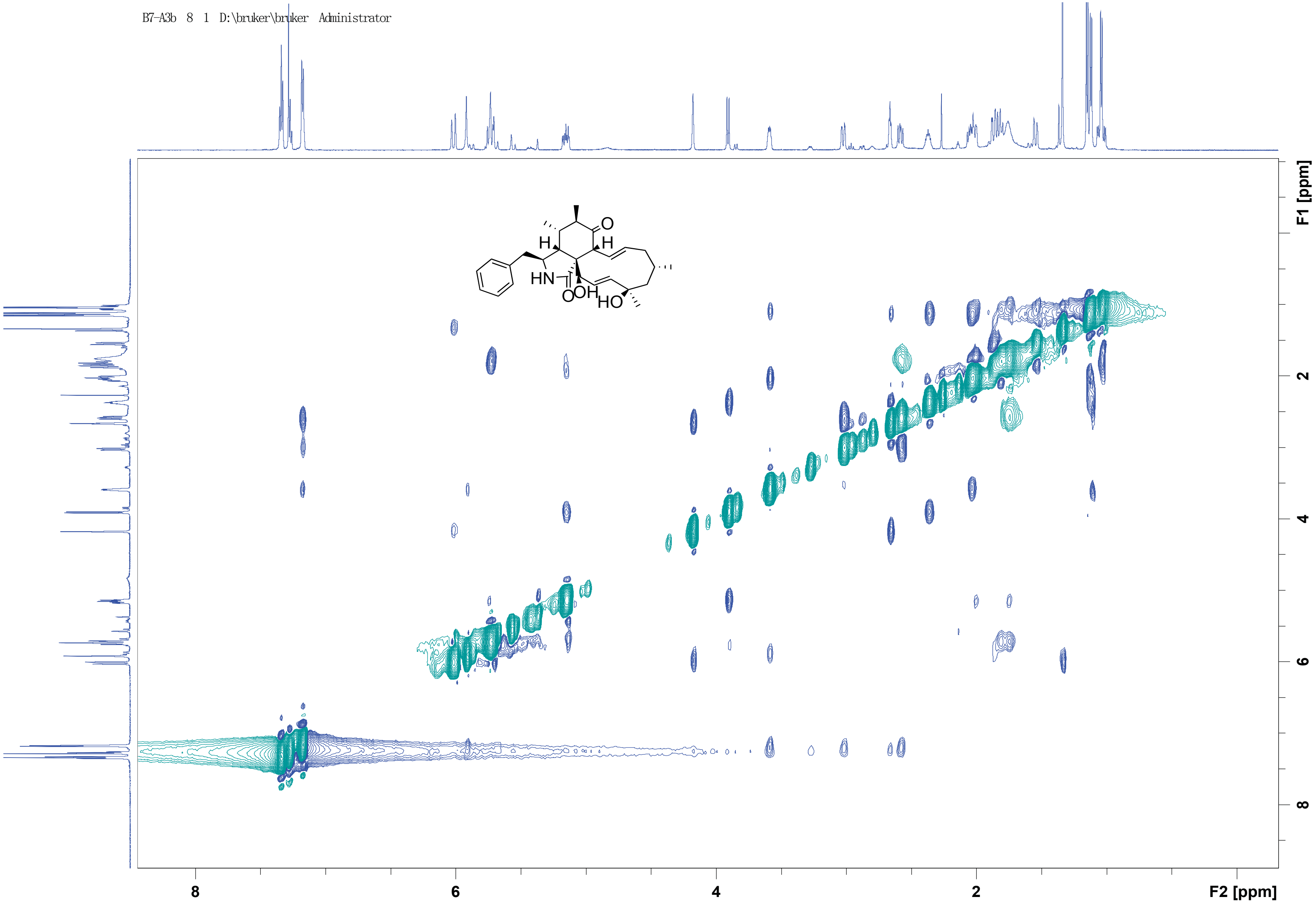

Figure S36. NOE spectrum of phomopchalasin C_6 (**6**)

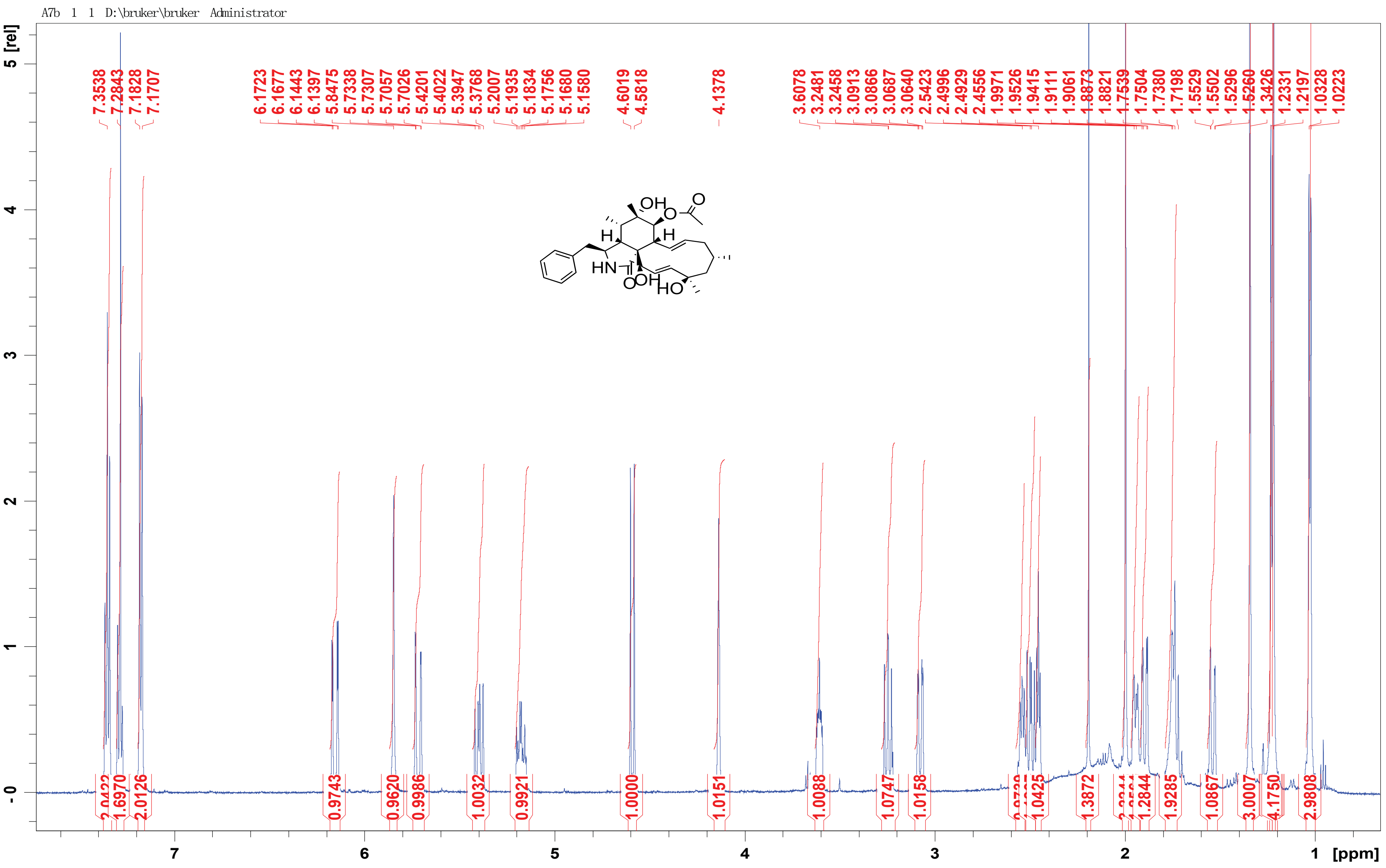

Figure S37. ^{1}H-NMR (600 MHz, $CDCl_3$) spectrum of phomopchalasin C_7 (**7**)

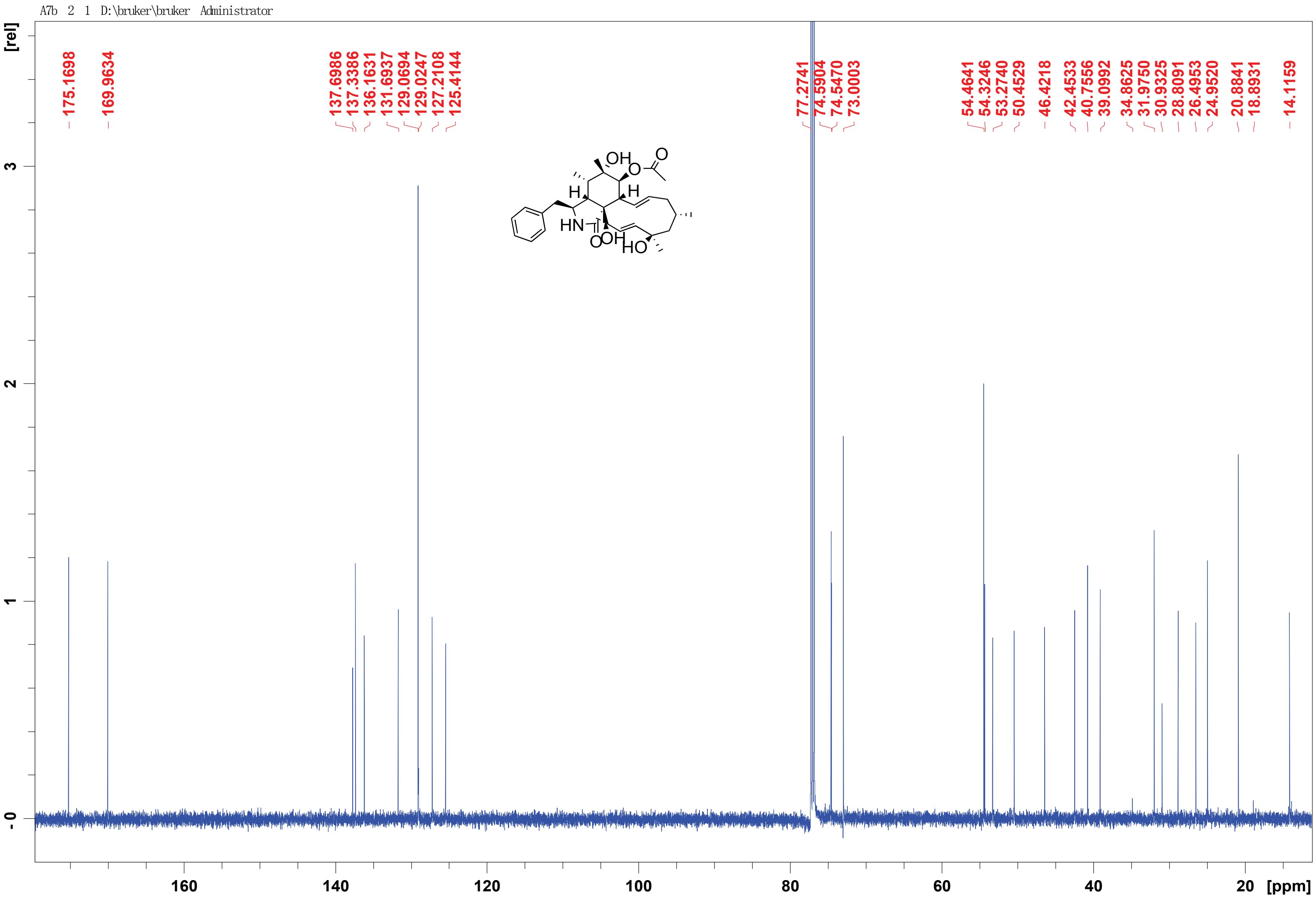

Figure S38. ^{13}C-NMR (150 MHz, $CDCl_3$) spectrum of phomopchalasin C_7 (**7**)

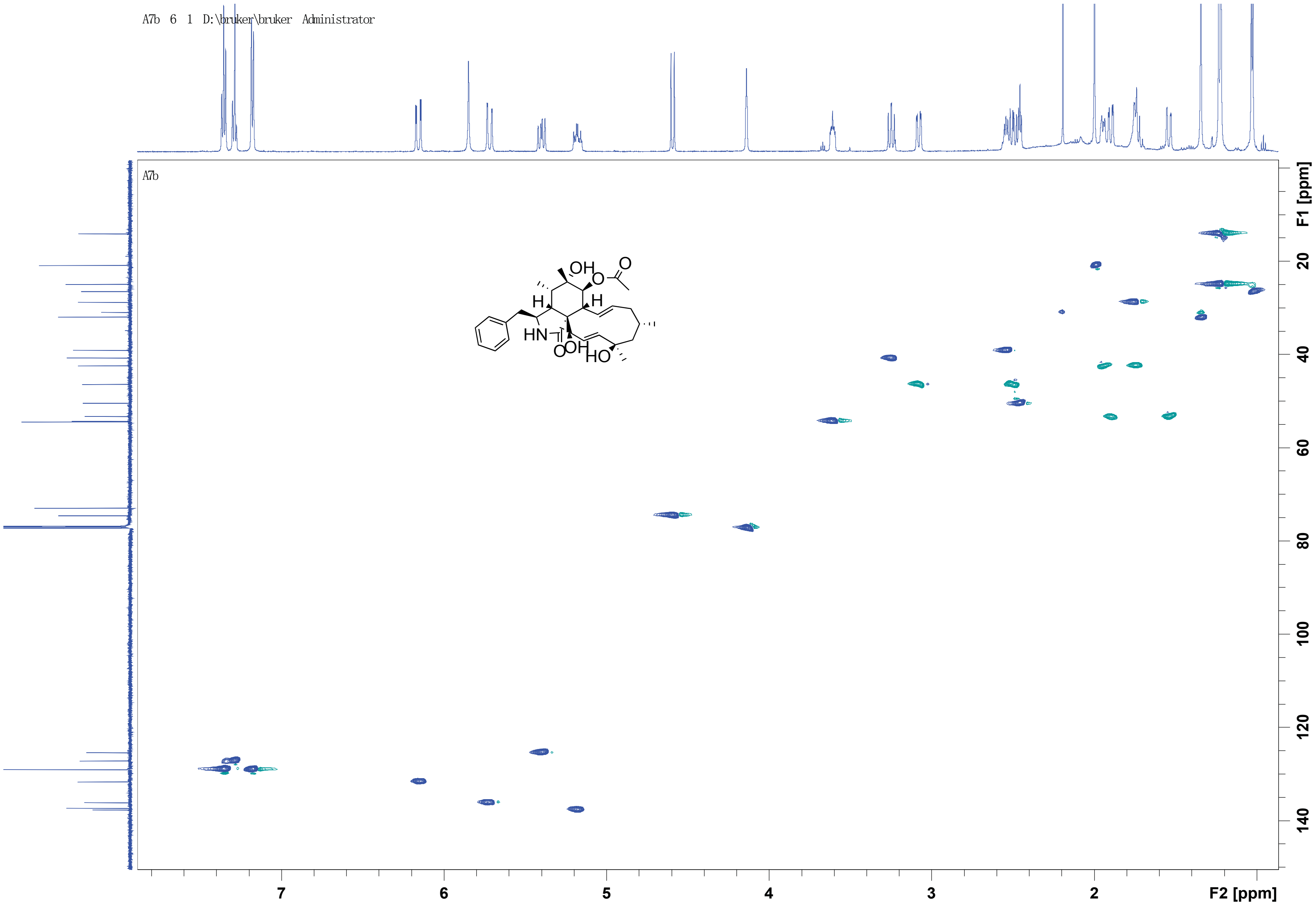

Figure S39. HSQC spectrum of phomopchalasin C_7 (**7**)

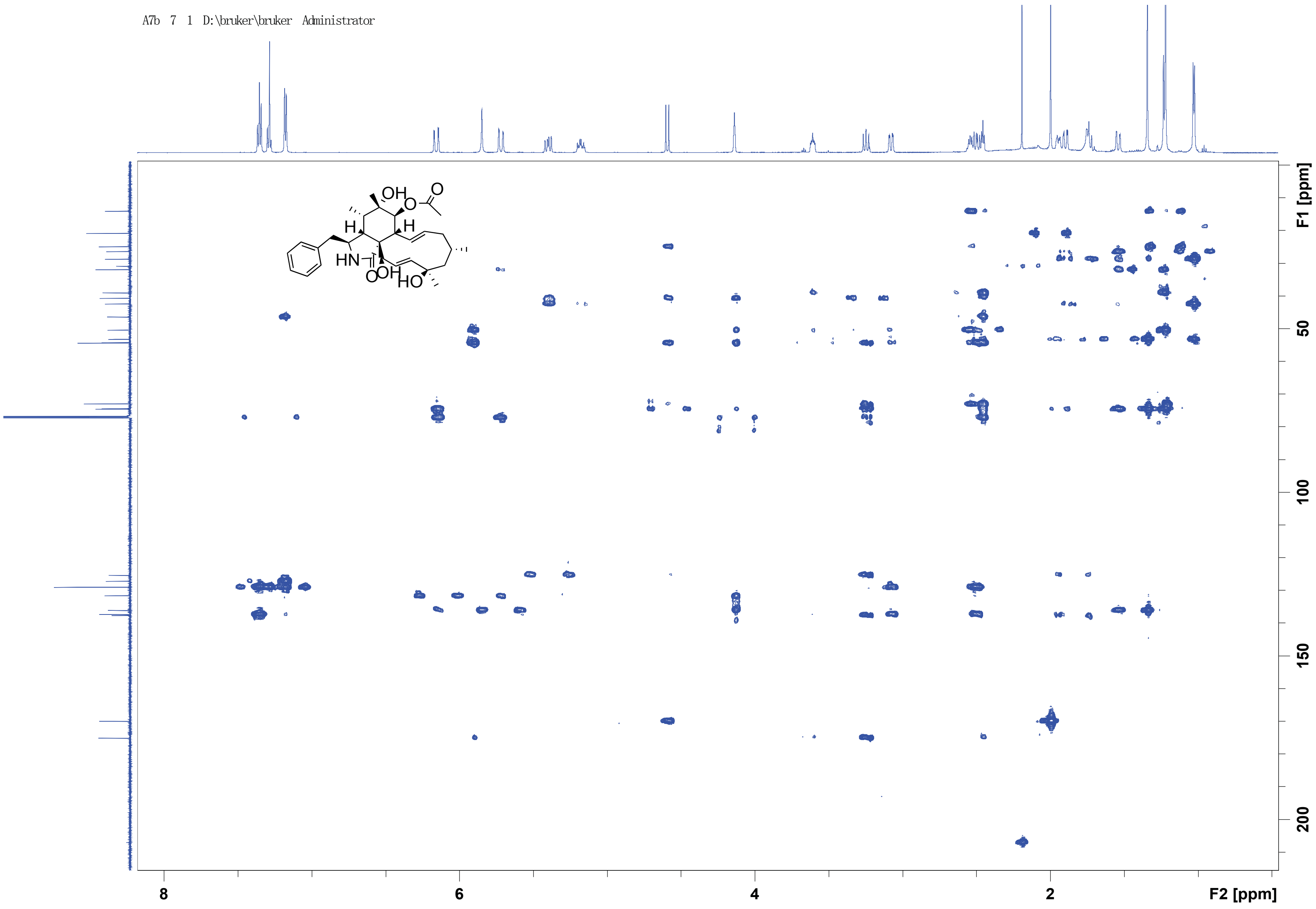

Figure S40. HMBC spectrum of phomopchalasin C_7 (**7**)

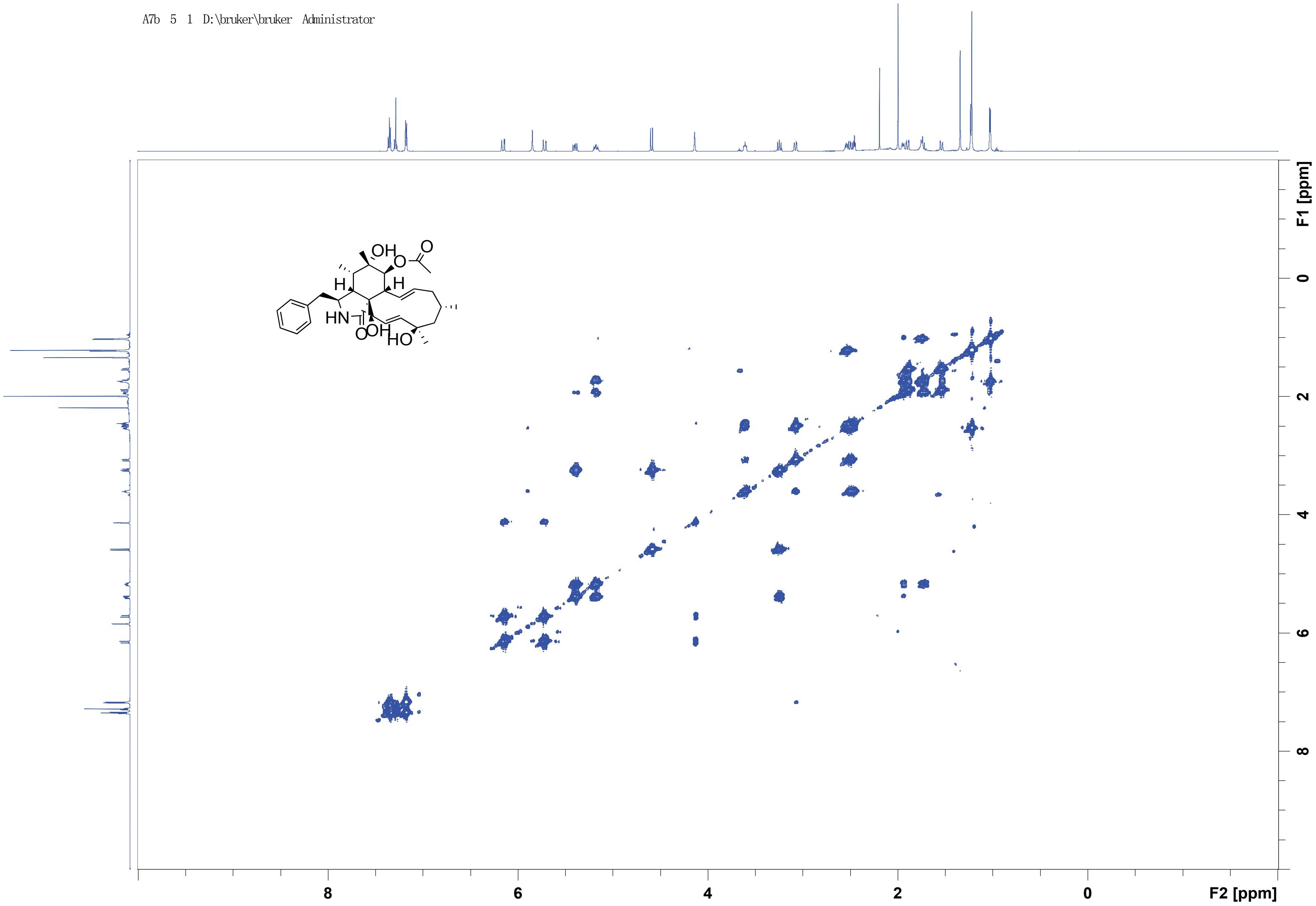

Figure S41. ^{1}H-^{1}H COSY spectrum of phomopchalasin C_7 (**7**)

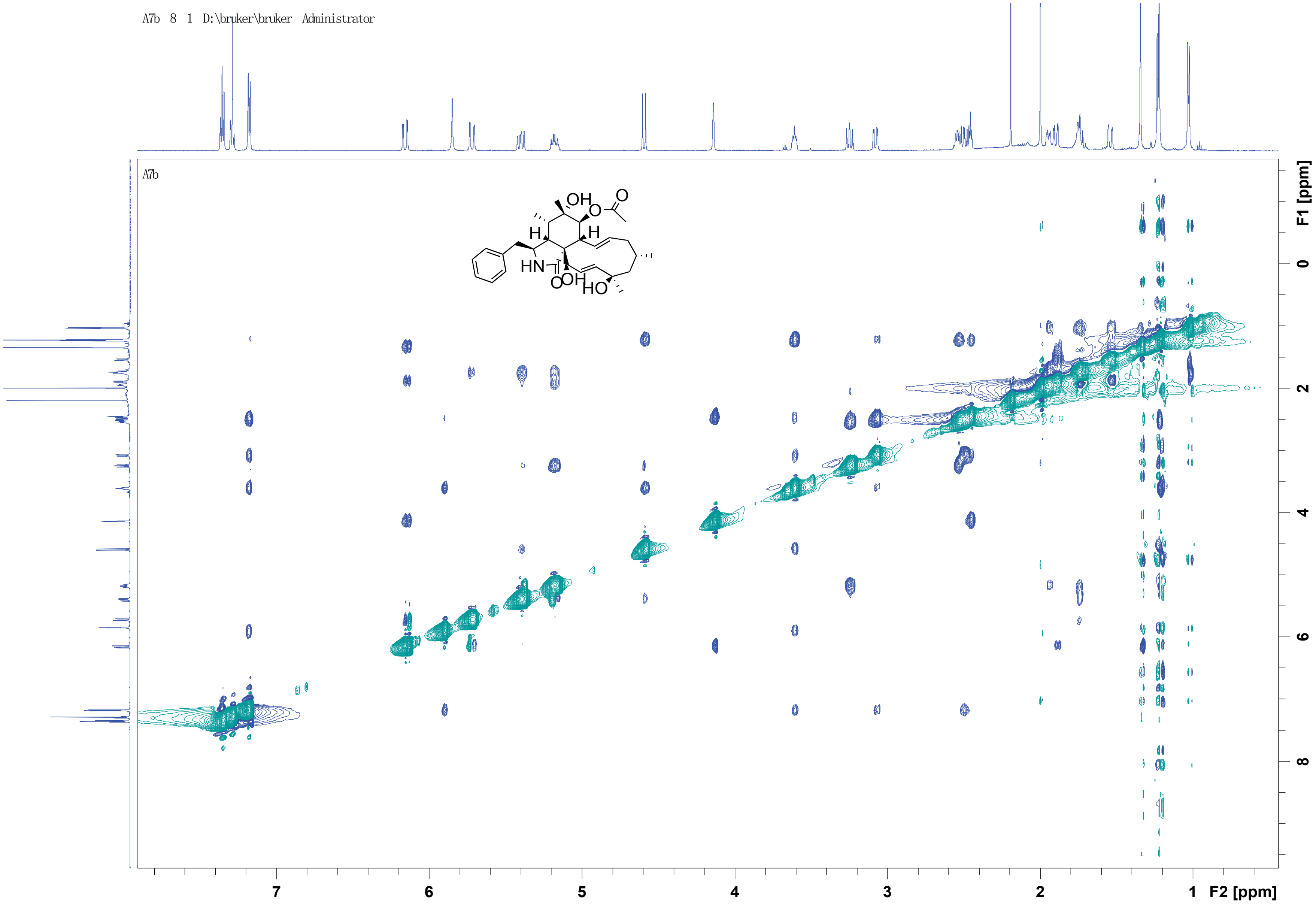

Figure S42. NOE spectrum of phomopchalasin C_7 (**7**)

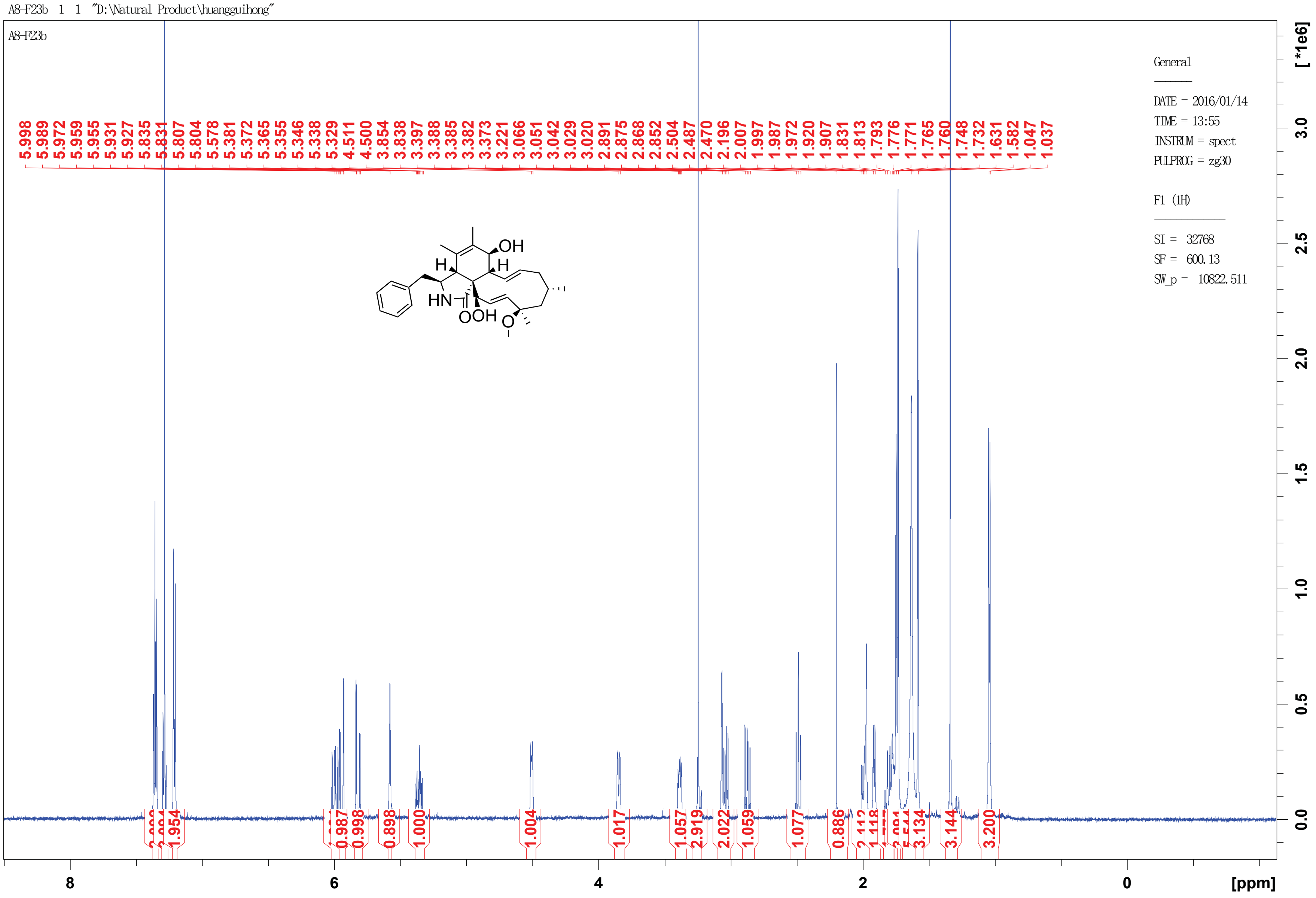

Figure S43. ^{1}H-NMR (600 MHz, $CDCl_3$) spectrum of phomopchalasin C_8 (**8**)

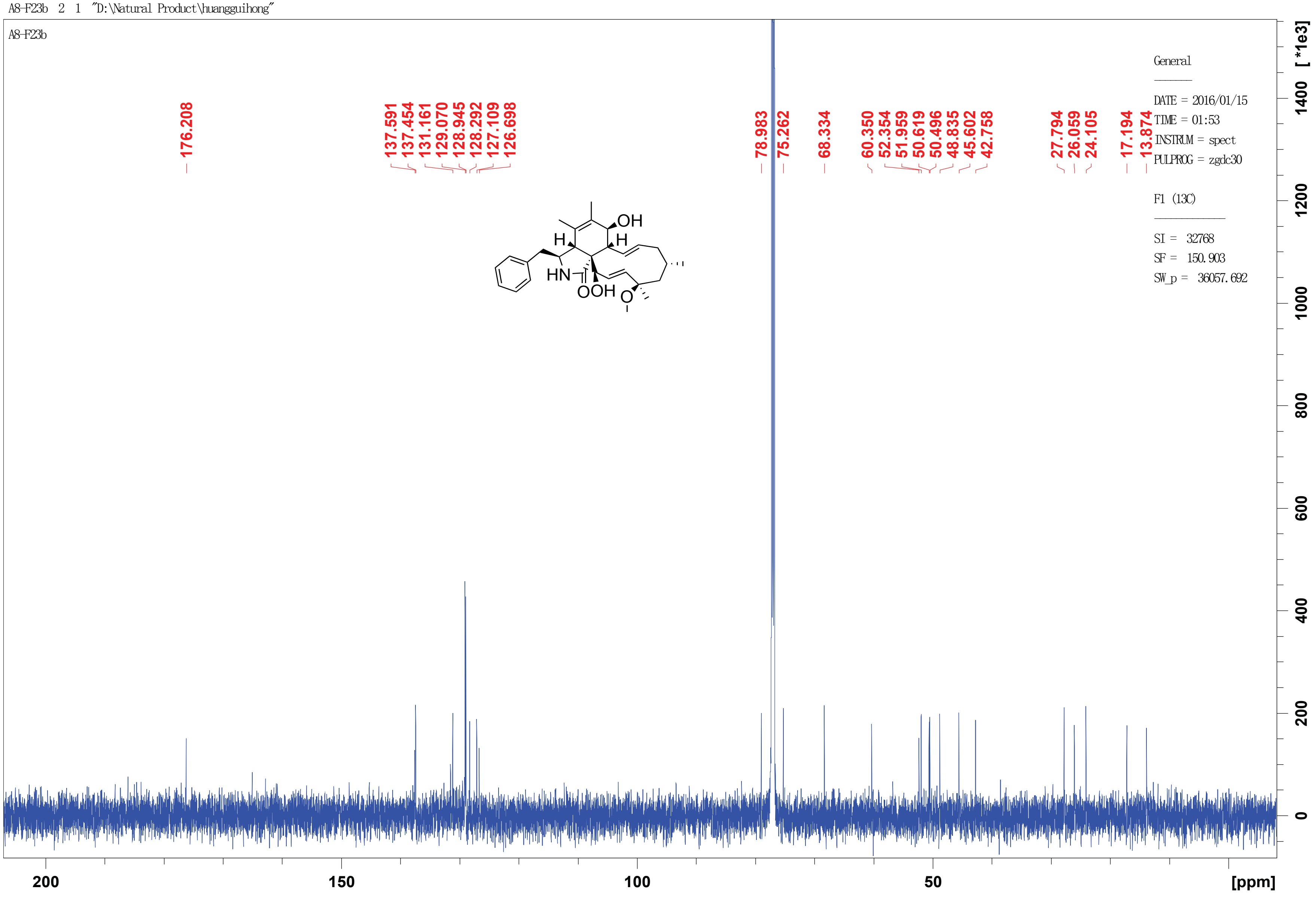

Figure S44. ^{13}C-NMR (150 MHz, $CDCl_3$) spectrum of phomopchalasin C_8 (**8**)

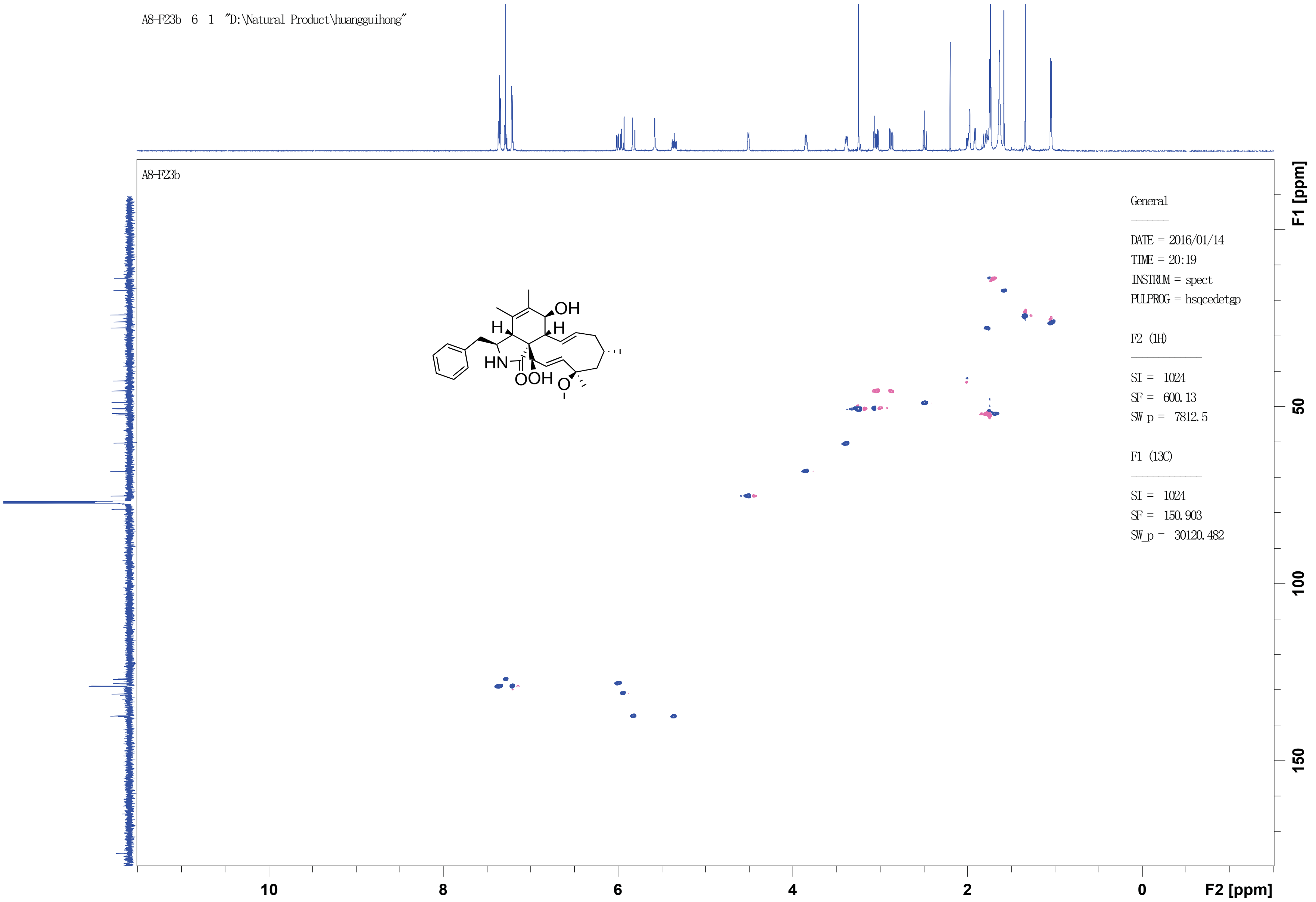

Figure S45. HSQC spectrum of phomopchalasin C_8 (**8**)

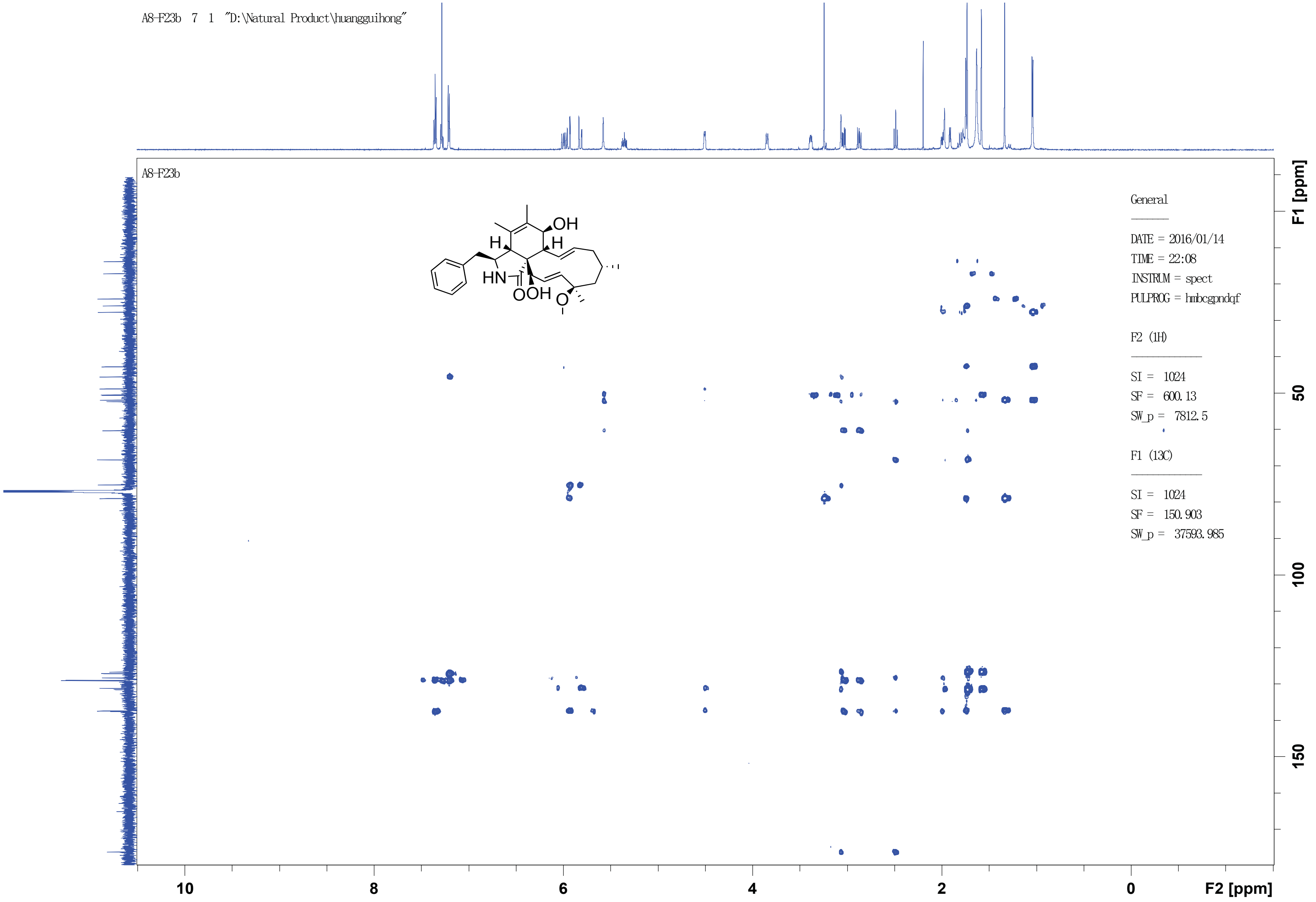

Figure S46. HMBC spectrum of phomopchalasin C_8 (**8**)

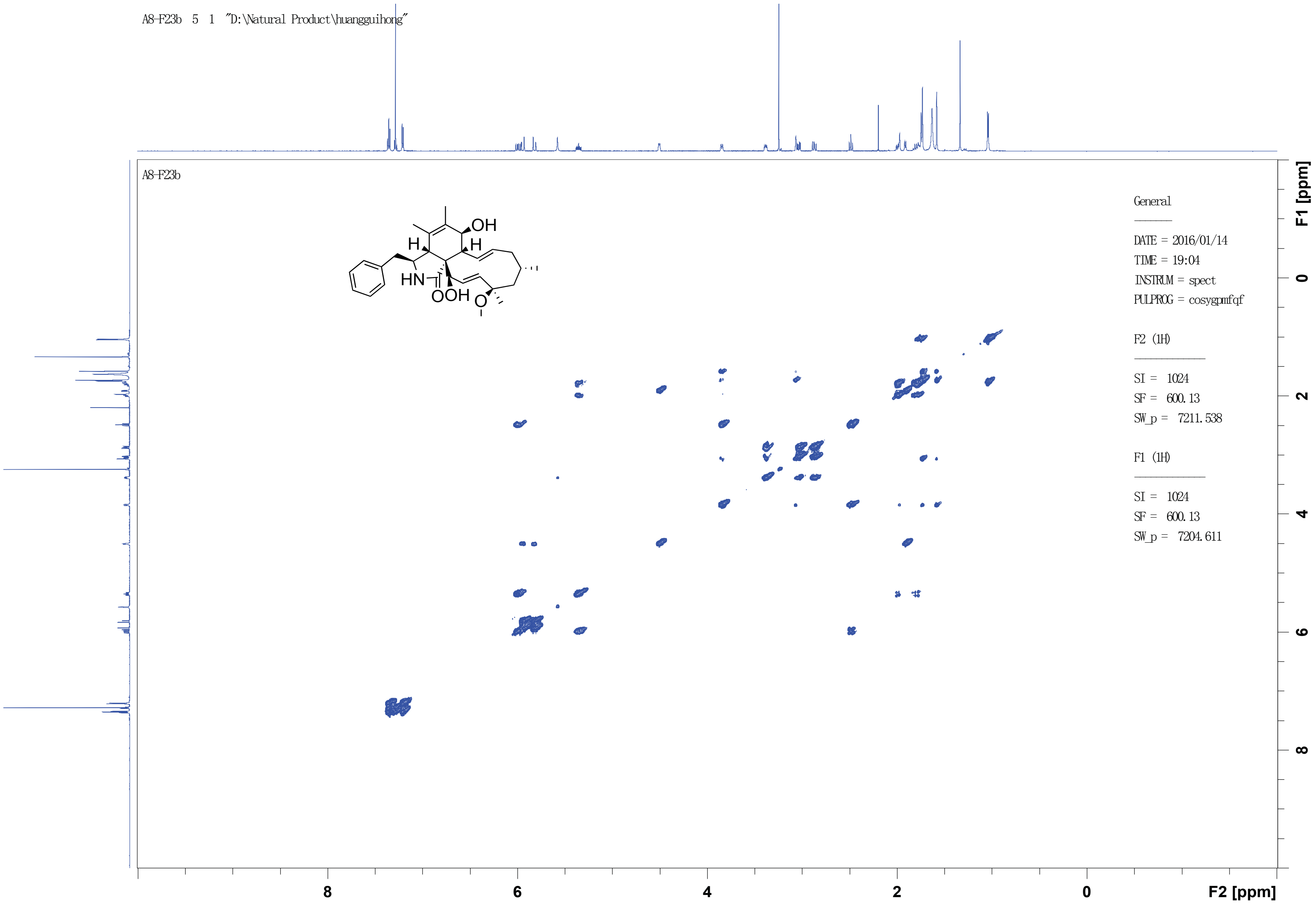

Figure S47. ^{1}H-^{1}H COSY spectrum of phomopchalasin C$_8$ (**8**)

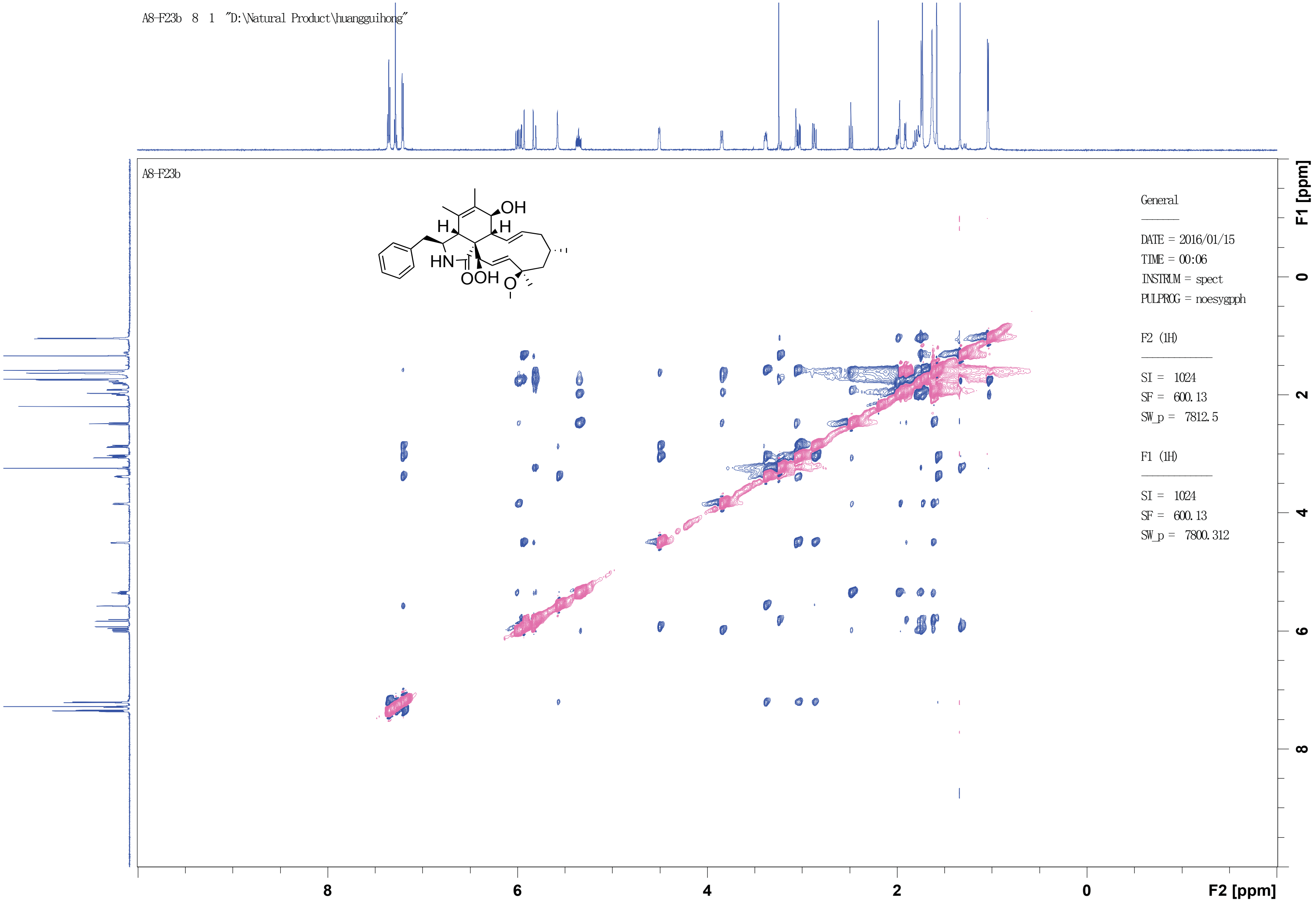

Figure S48. NOE spectrum of phomopchalasin C_8 (**8**)

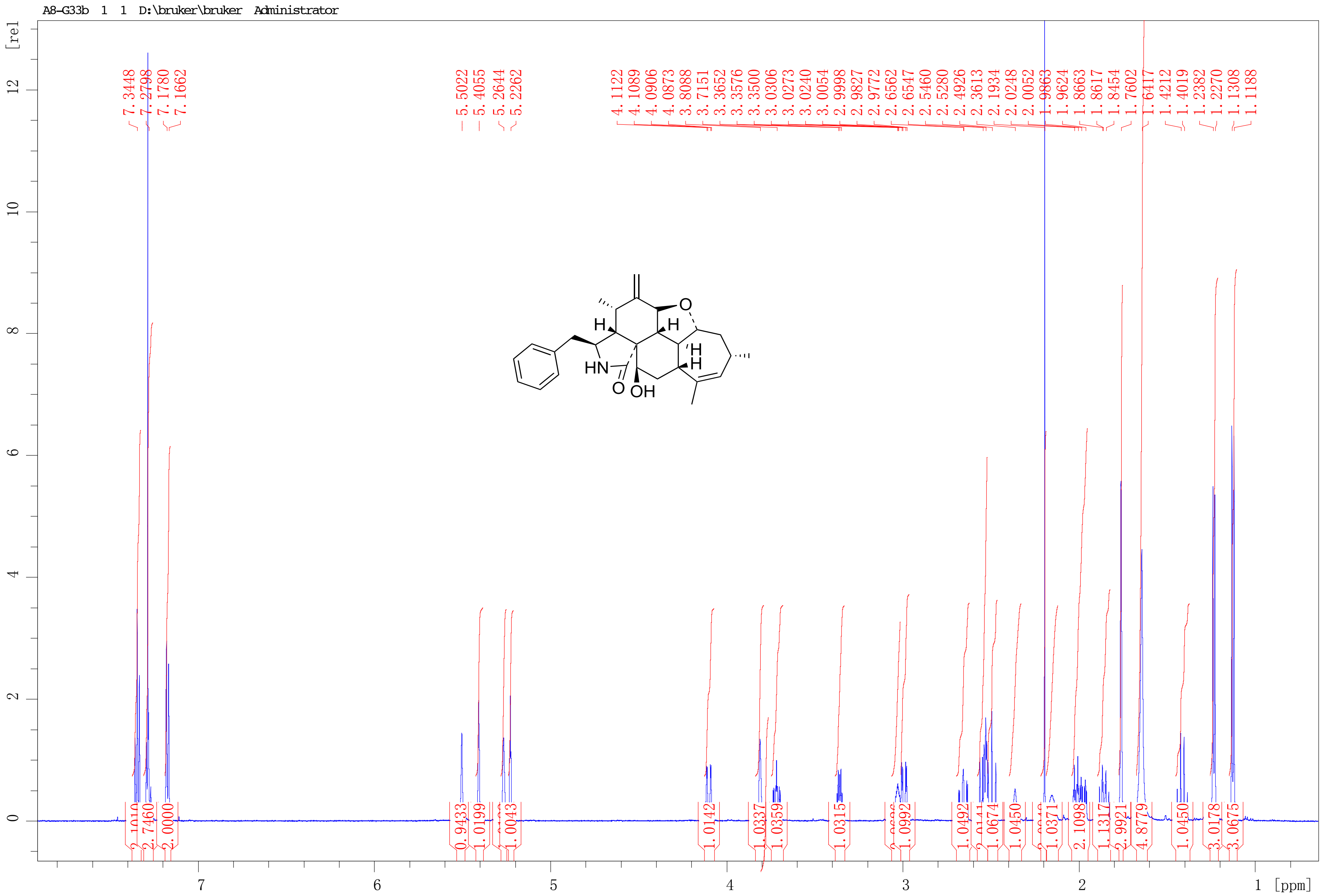

Figure S49. ^{1}H-NMR (600 MHz, $CDCl_3$) spectrum of cytochalasin J_3 (**9**)

A8-G33b 2 1 D:\bruker\bruker Administrator

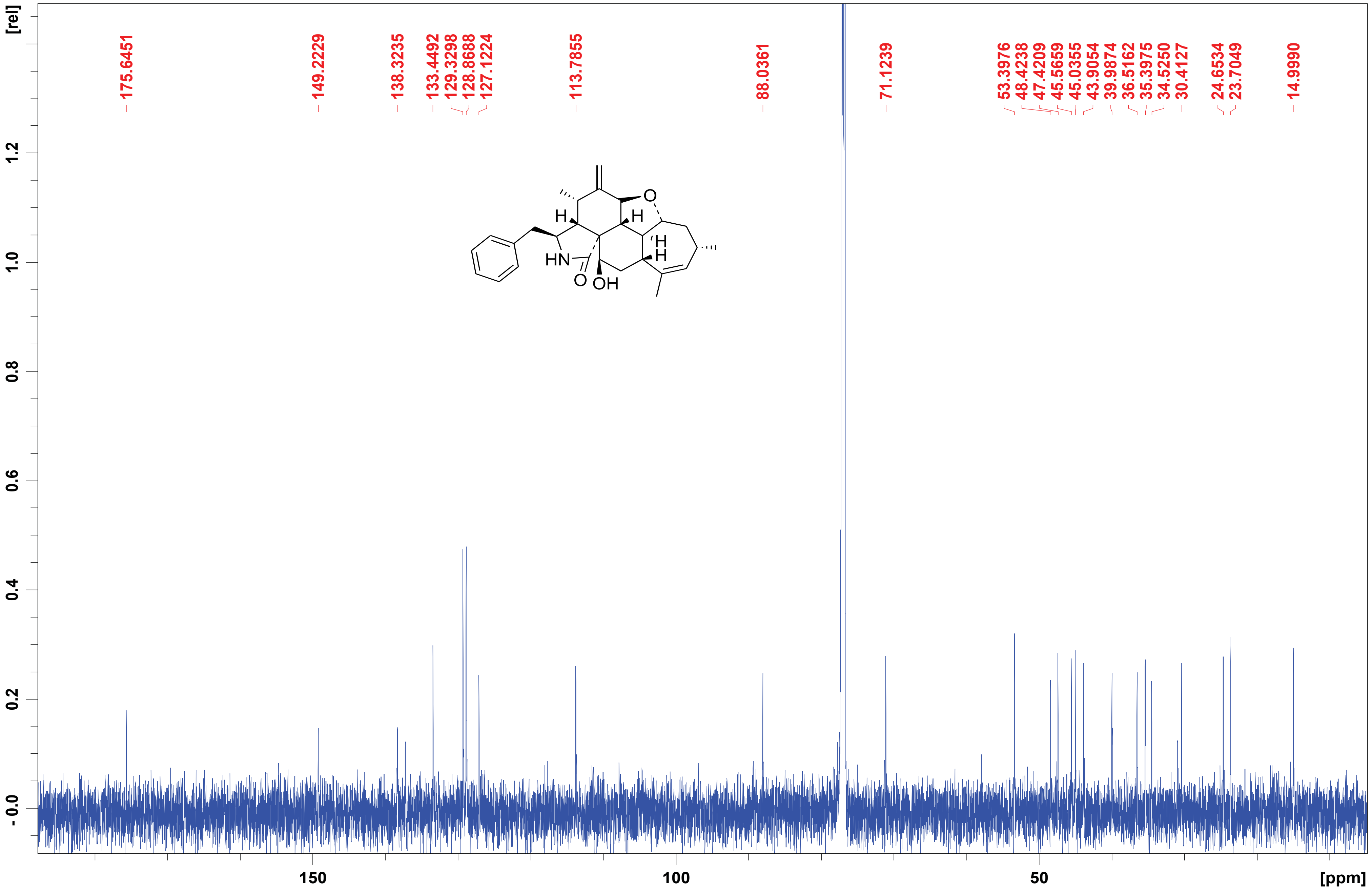

Figure S50. ^{13}C-NMR (150 MHz, $CDCl_3$) spectrum of cytochalasin J_3 (**9**)

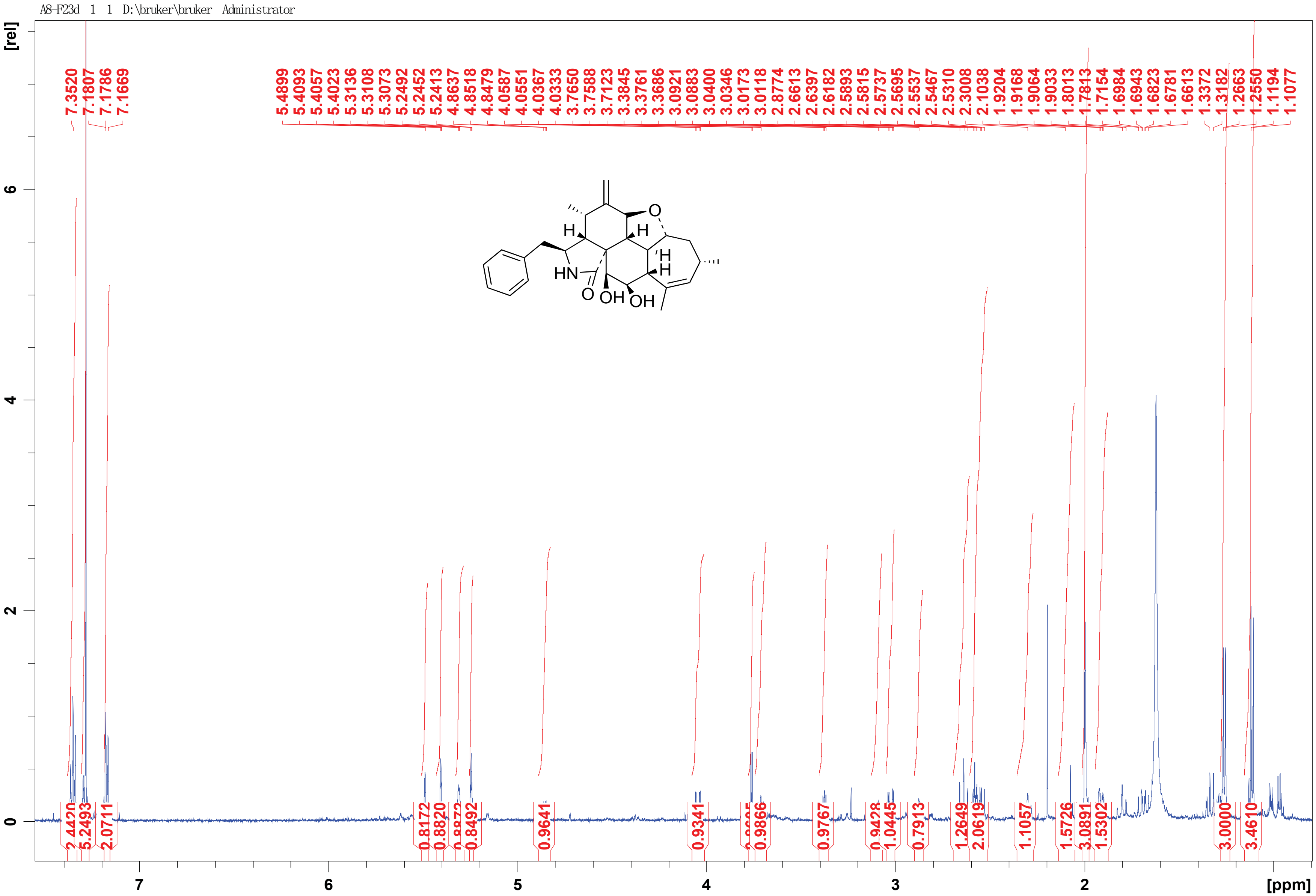

Figure S51. ^{1}H-NMR (600 MHz, $CDCl_3$) spectrum of phomopchalasin B (**10**)

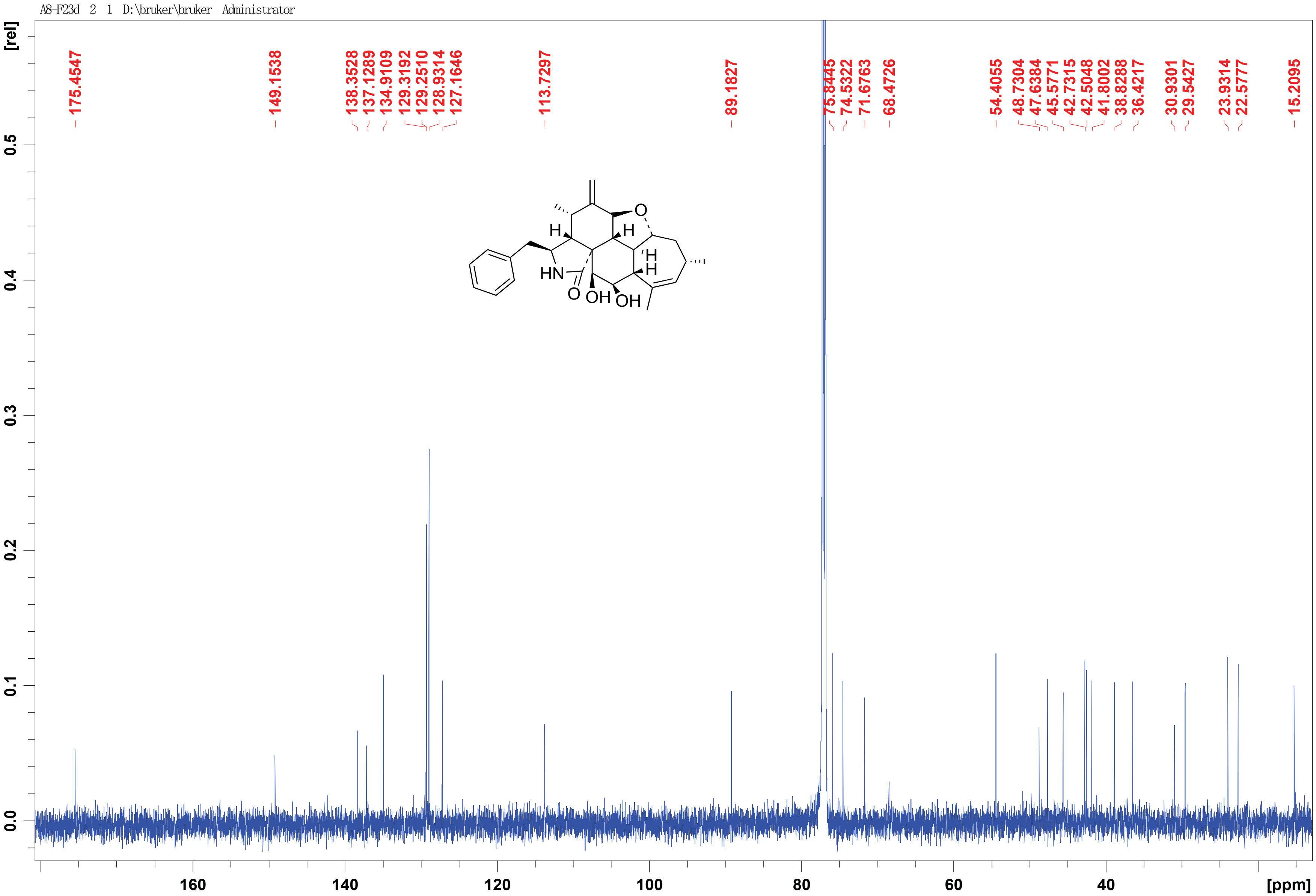

Figure S52. ^{13}C-NMR (150 MHz, $CDCl_3$) spectrum of phomopchalasin B (**10**)

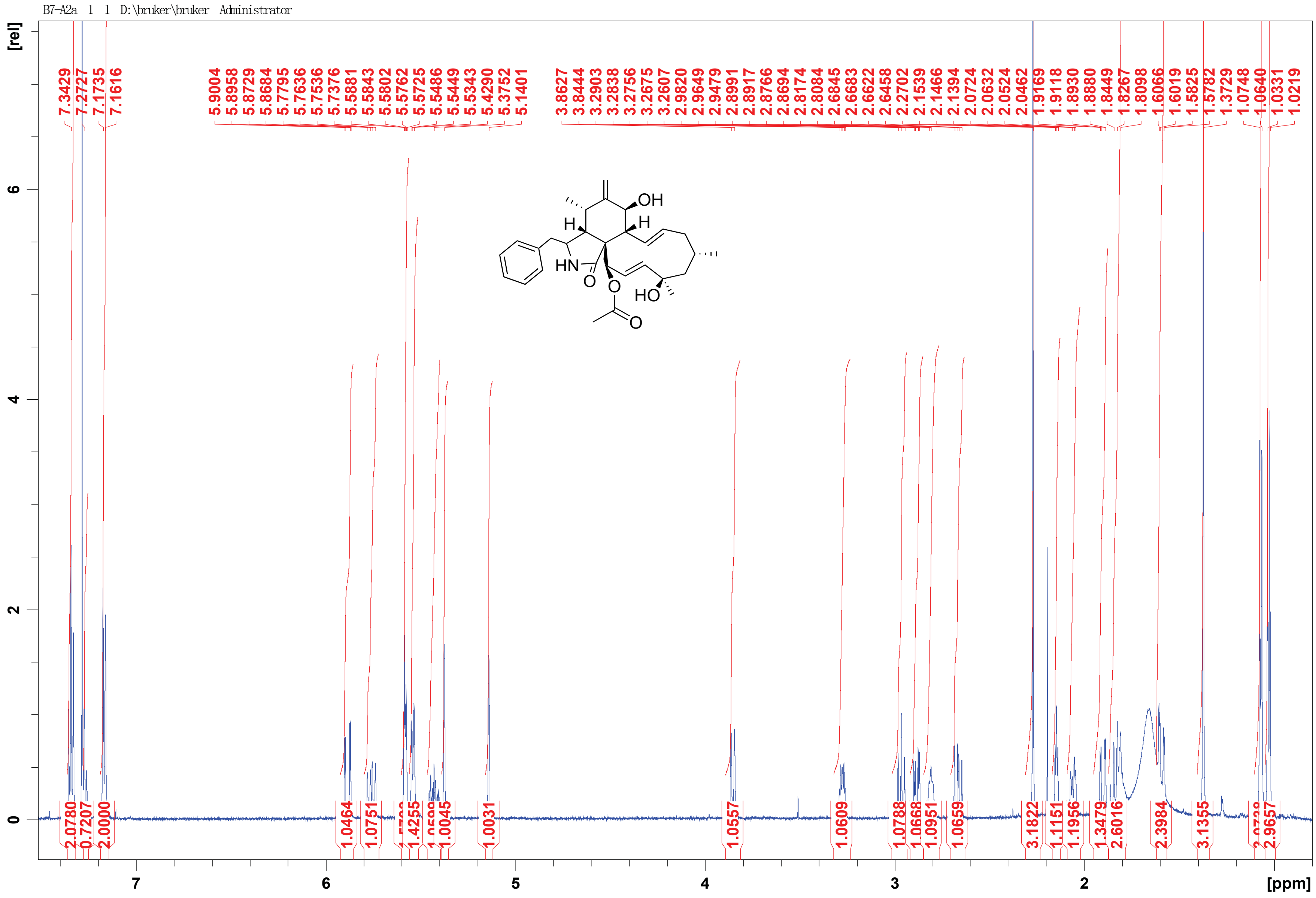

Figure S53. ¹H-NMR (600 MHz, $CDCl_3$) spectrum of cytochalasin H (**11**)

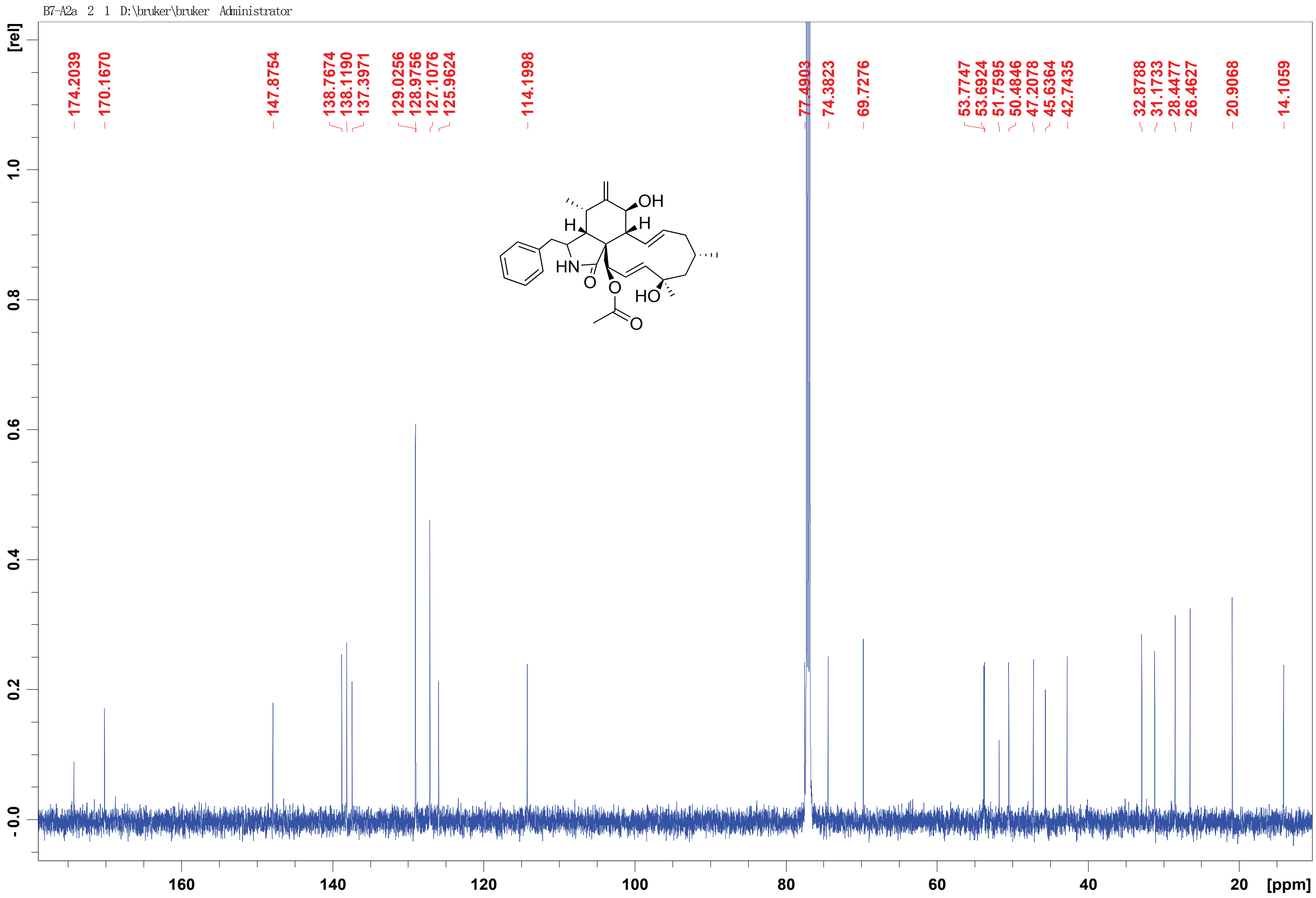

Figure S54. ^{13}C-NMR (150 MHz, $CDCl_3$) spectrum of cytochalasin H (**11**)

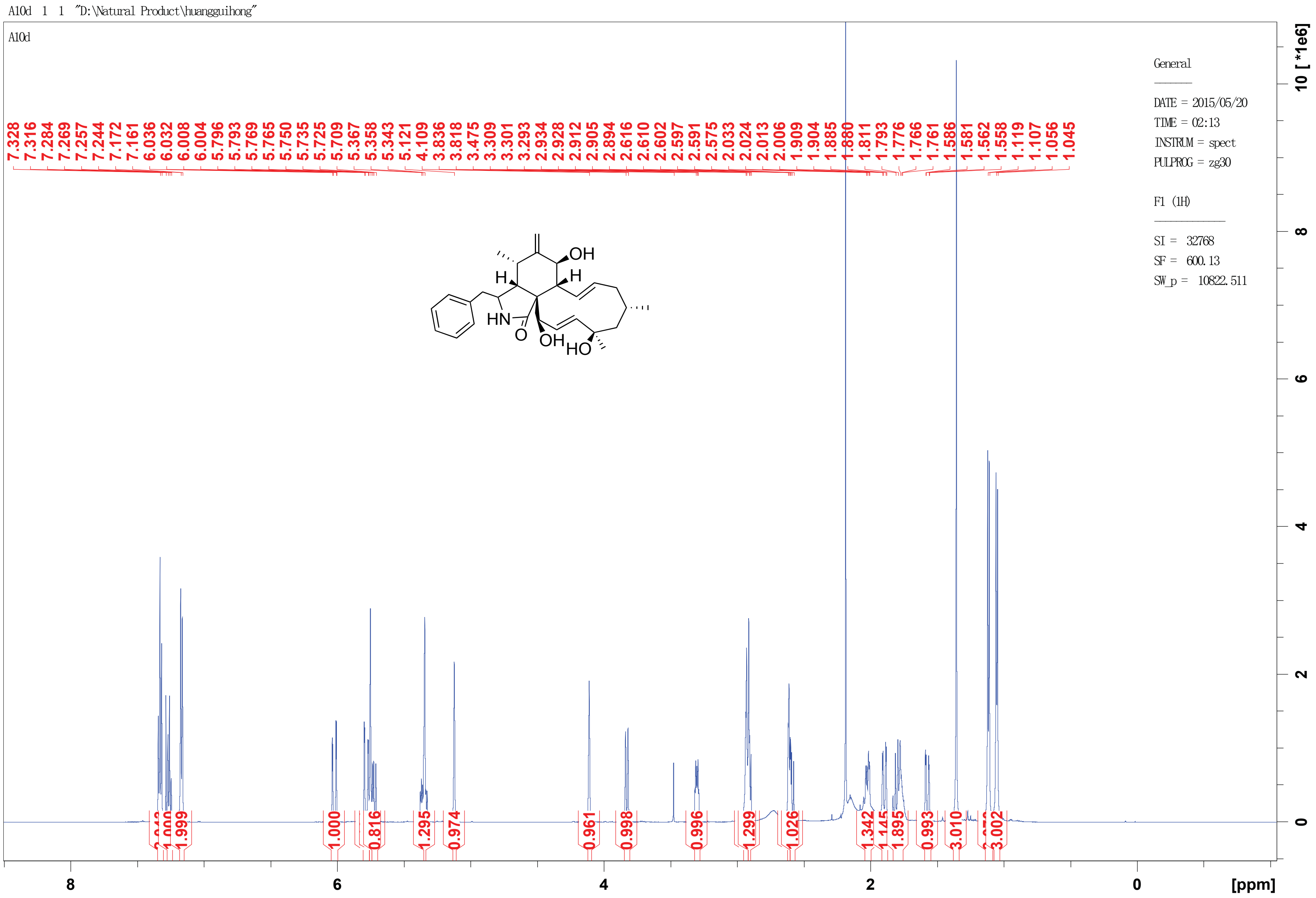

Figure S55. ^{1}H-NMR (600 MHz, $CDCl_3$) spectrum of cytochalasin J (**12**)

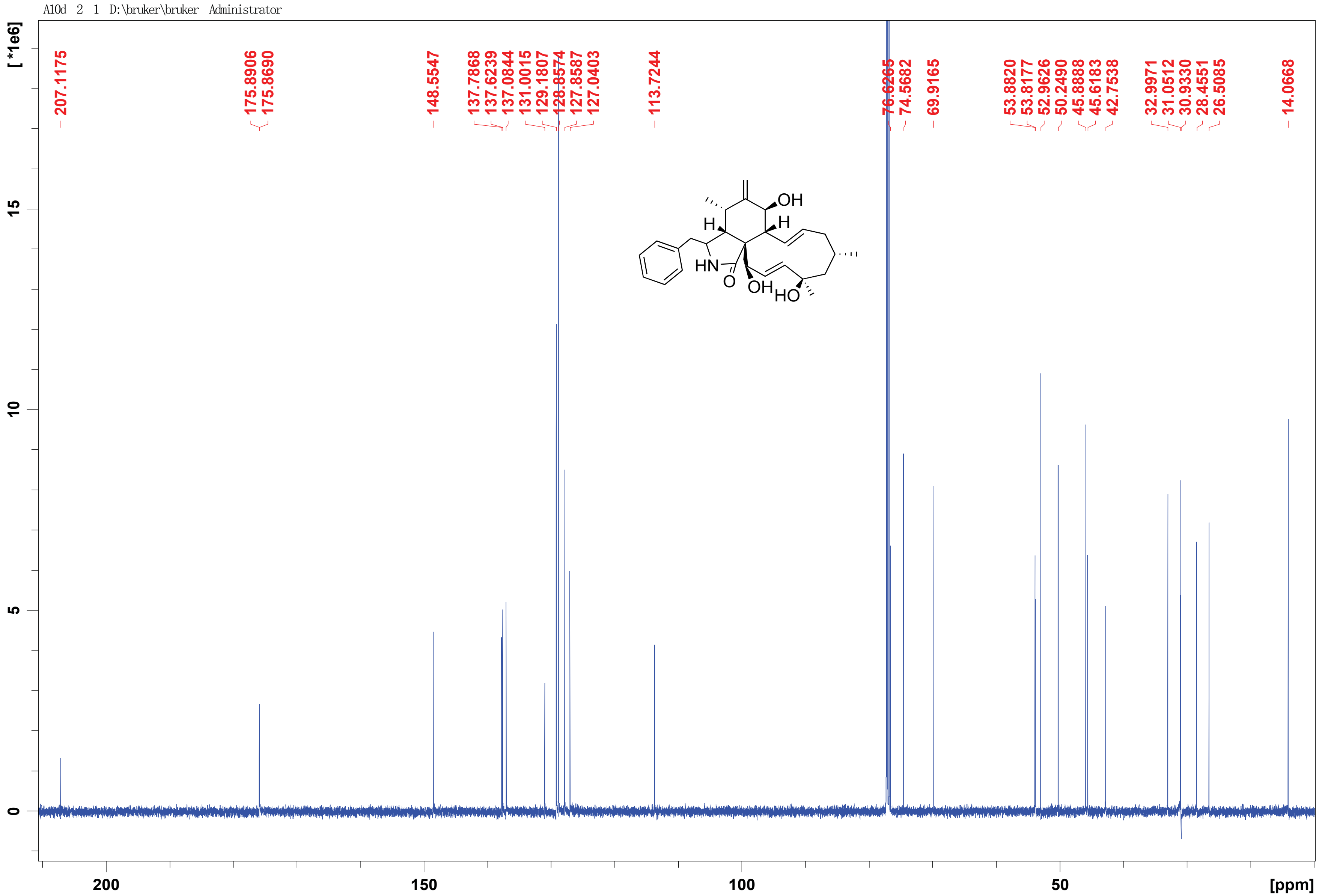

Figure S56. ^{13}C-NMR (150 MHz, $CDCl_3$) spectrum of cytochalasin J (**12**)

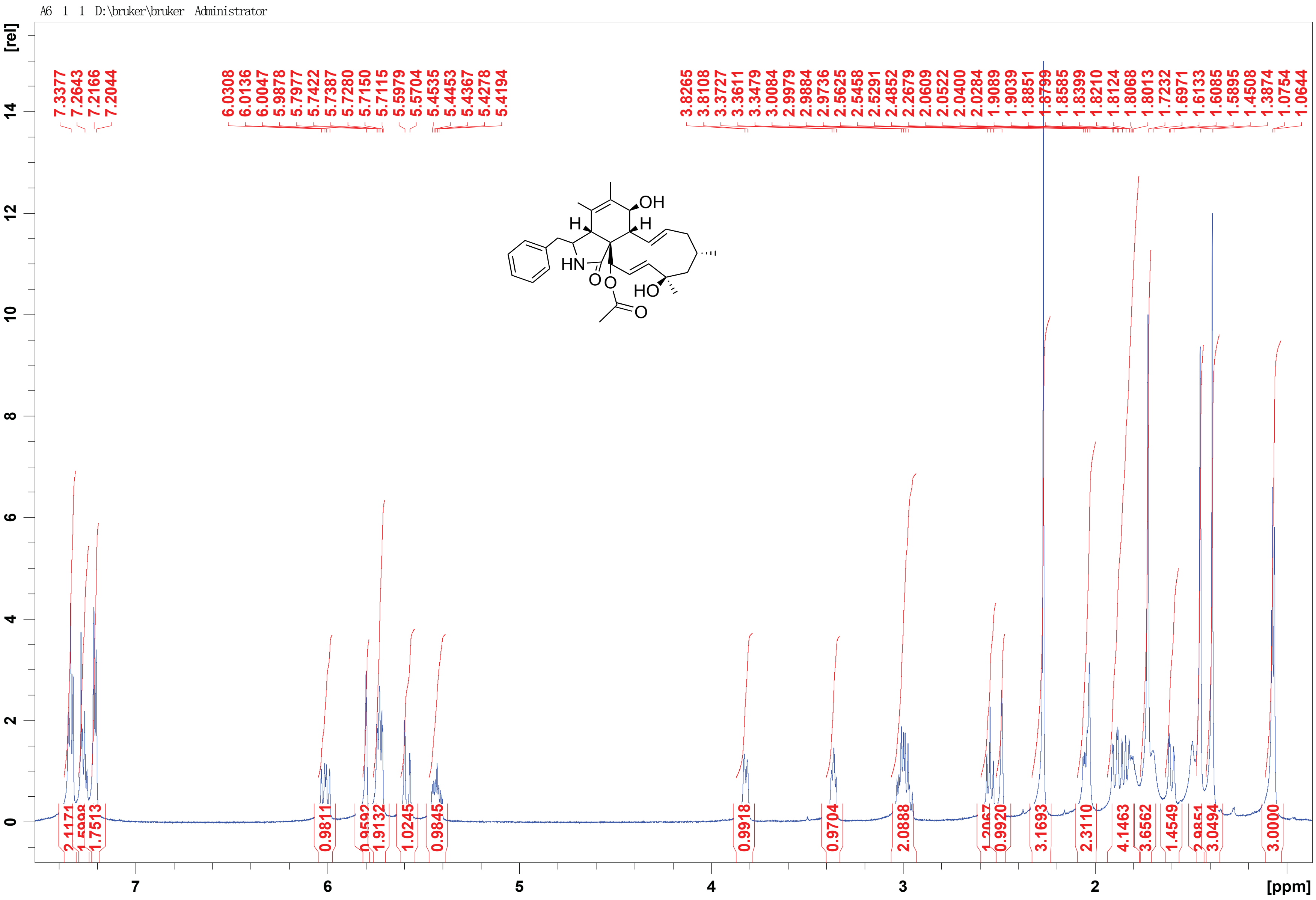

Figure S57. ^{1}H-NMR (600 MHz, $CDCl_3$) spectrum of cytochalasin N (**13**)

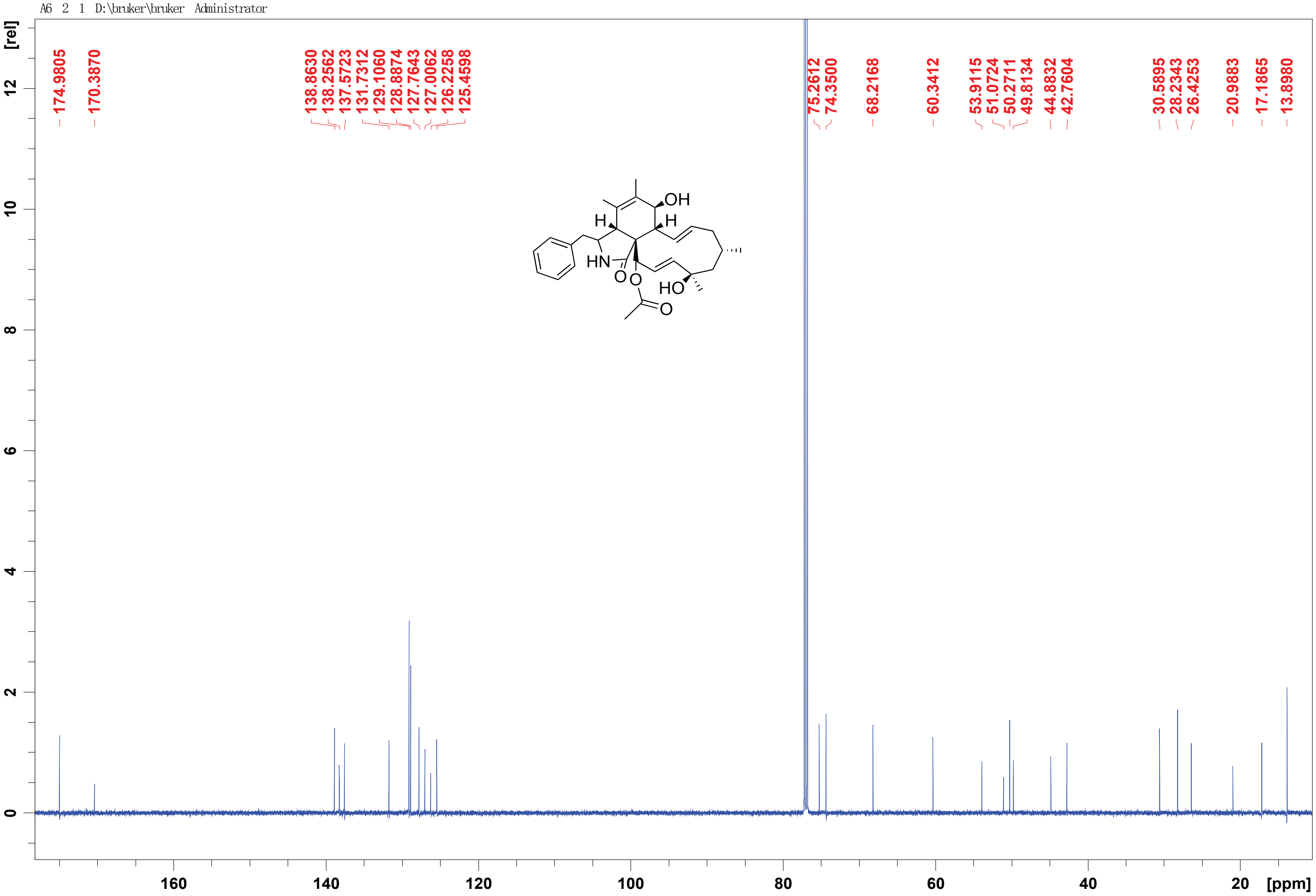

Figure S58. ^{13}C-NMR (150 MHz, $CDCl_3$) spectrum of cytochalasin N (**13**)

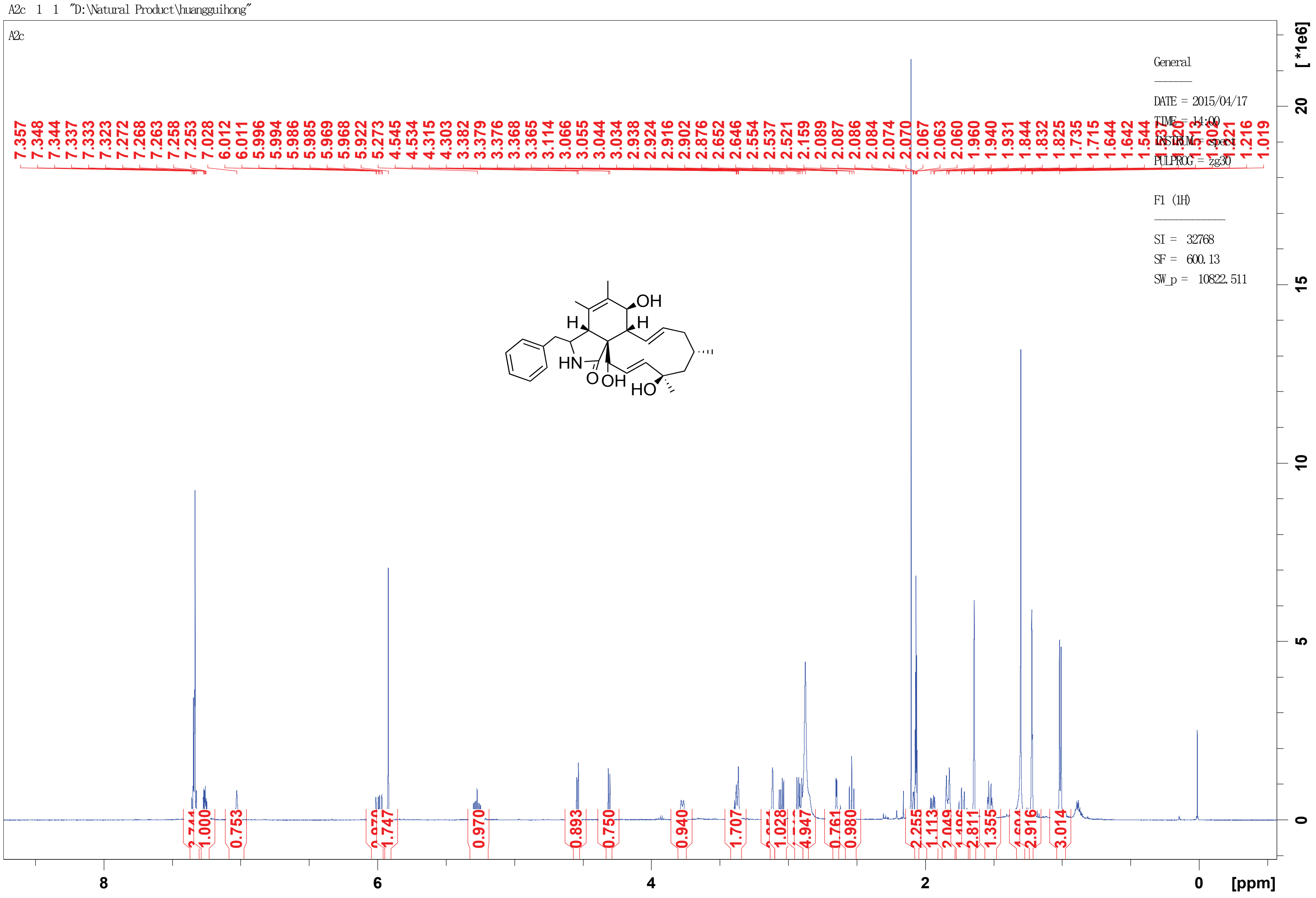

Figure S59. ^{1}H-NMR (600 MHz, $CDCl_3$) spectrum of cytochalasin O (**14**)

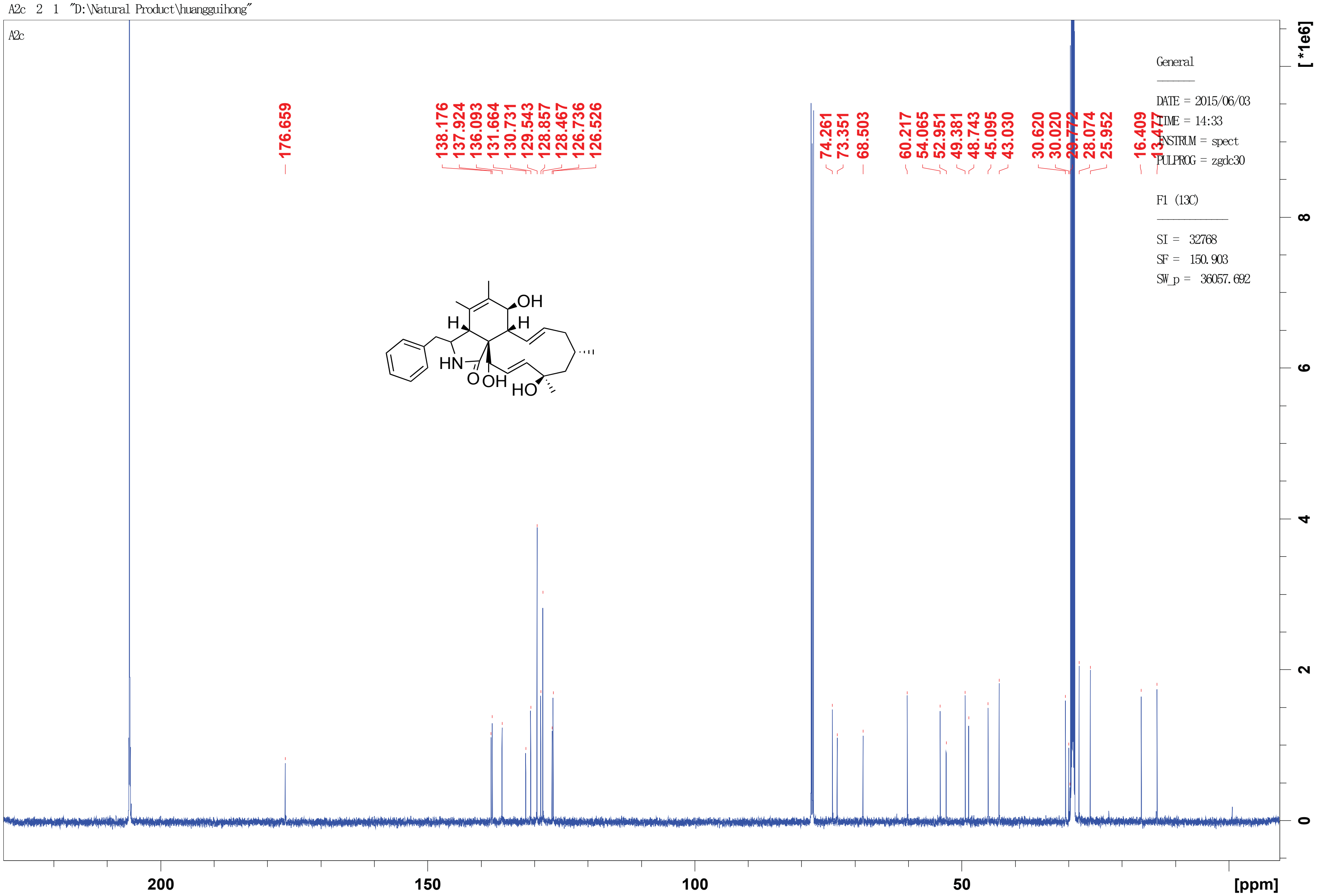

Figure S60. ^{13}C-NMR (150 MHz, $CDCl_3$) spectrum of cytochalasin O (**14**)

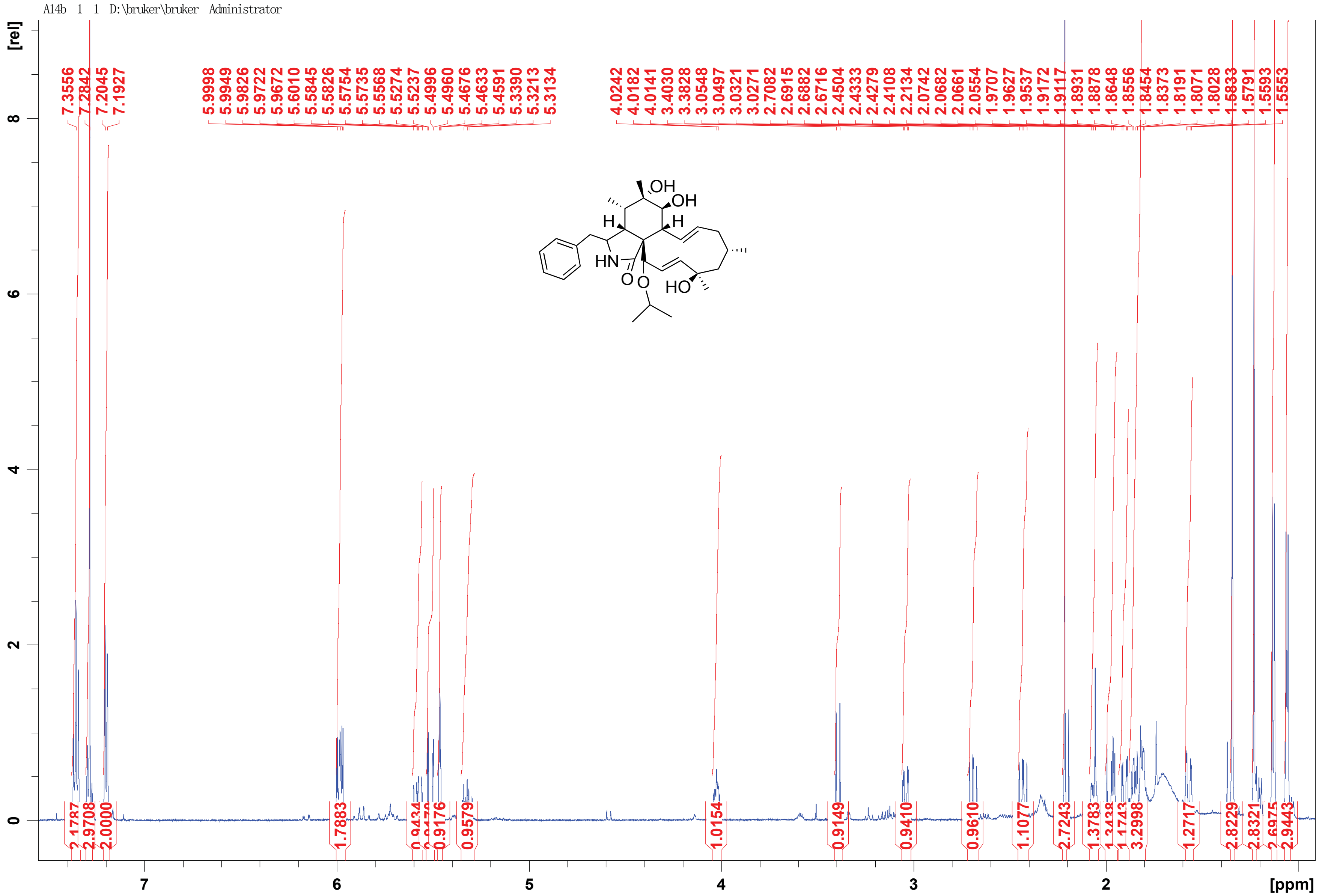

Figure S61. ^{1}H-NMR (600 MHz, $CDCl_3$) spectrum of cytochalasin N (**15**)

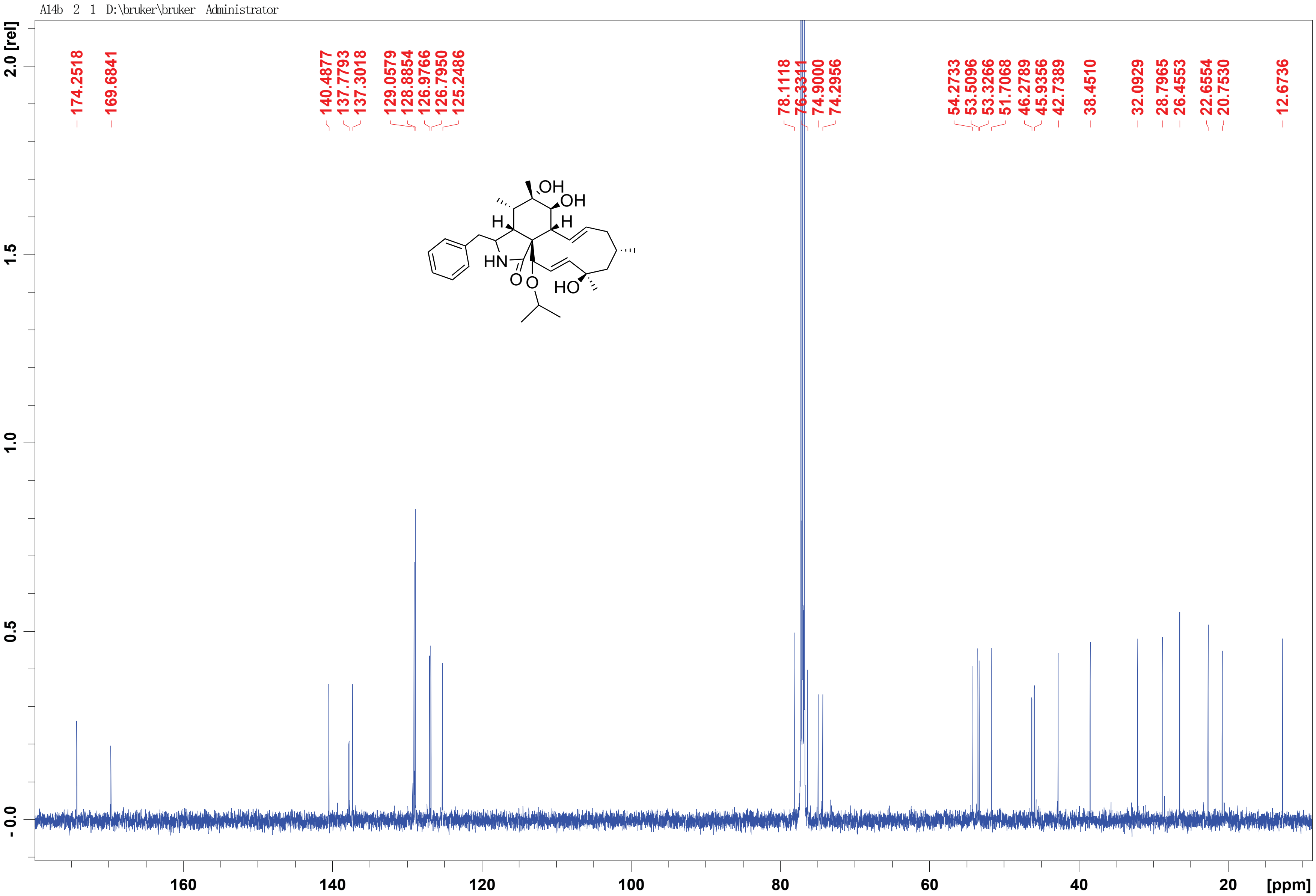

Figure S62. ^{13}C-NMR (150 MHz, $CDCl_3$) spectrum of cytochalasin N (**15**)

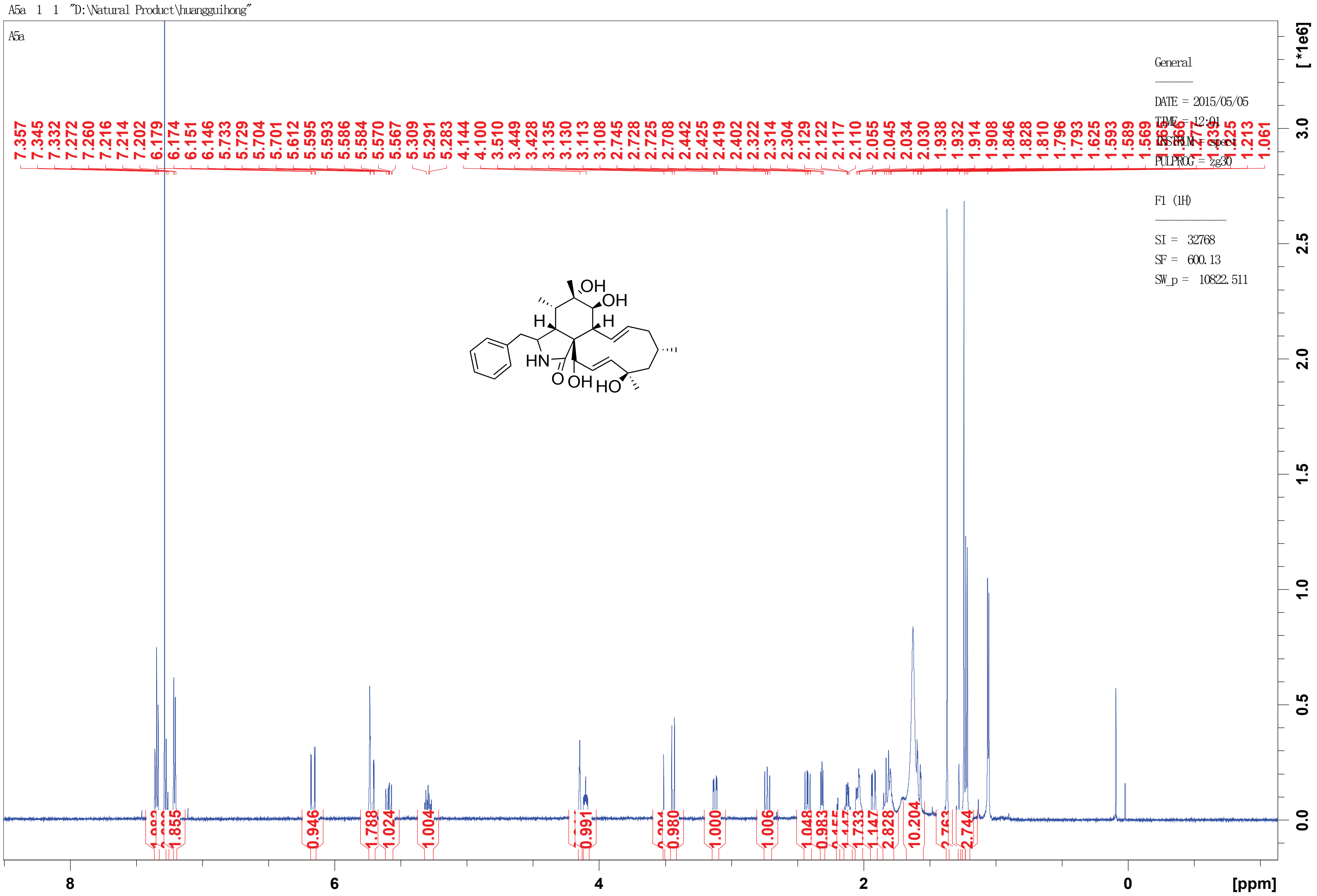

Figure S63. ^{1}H-NMR (600 MHz, $CDCl_3$) spectrum of cytochalasin Q (**16**)

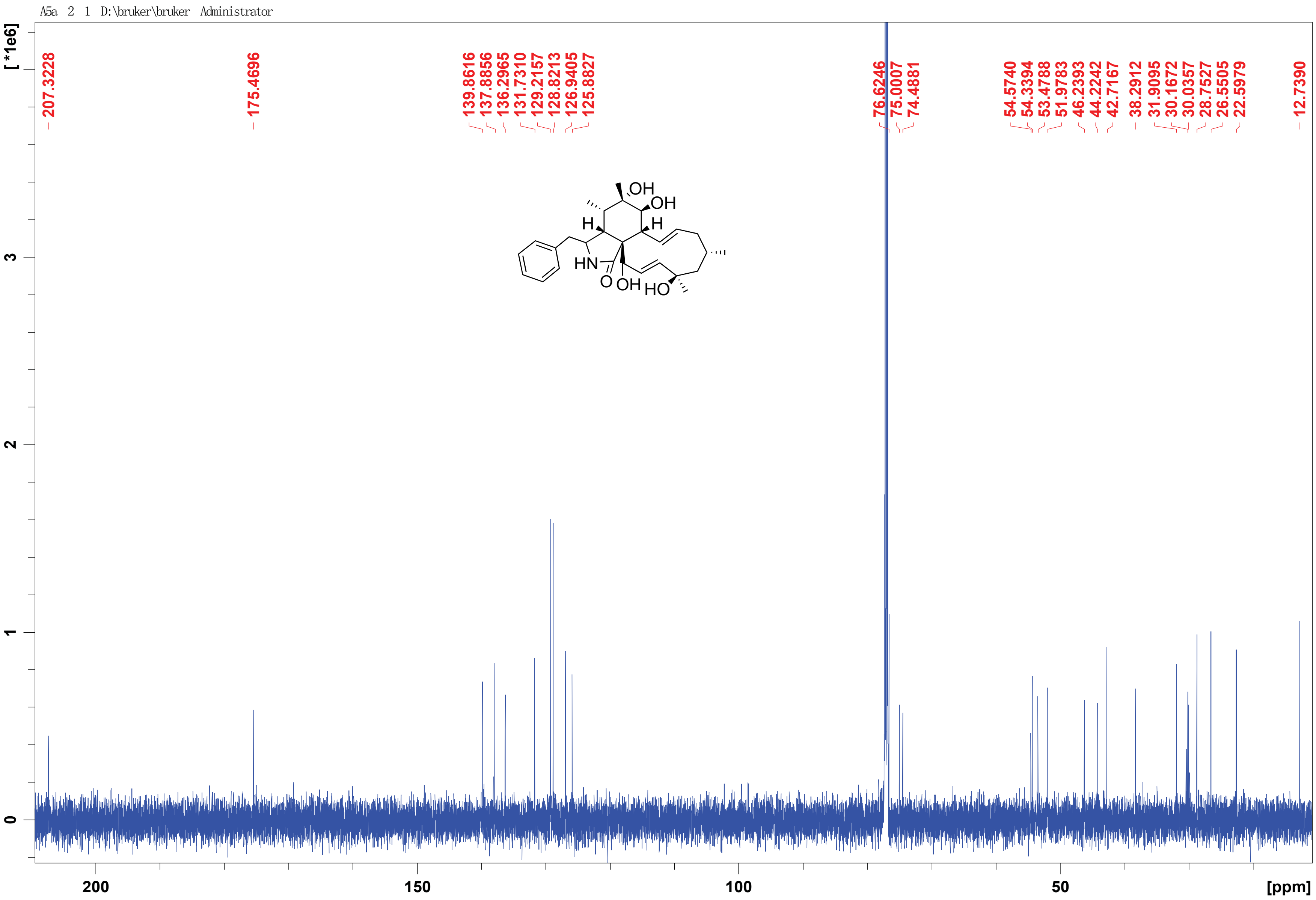

Figure S64. ^{13}C-NMR (150 MHz, $CDCl_3$) spectrum of cytochalasin Q (**16**)

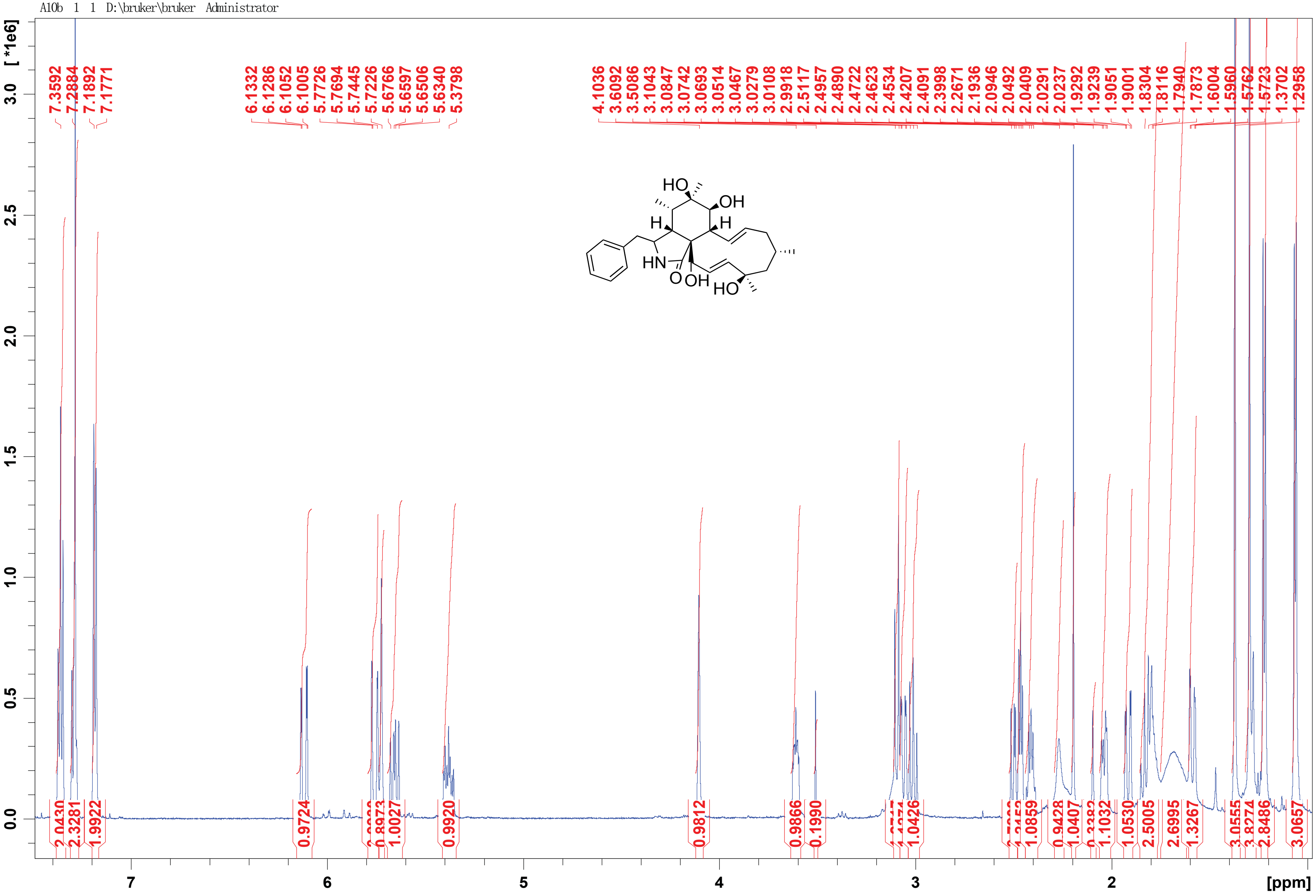

Figure S65. ^{1}H-NMR (600 MHz, $CDCl_3$) spectrum of cytochalasin R (**17**)

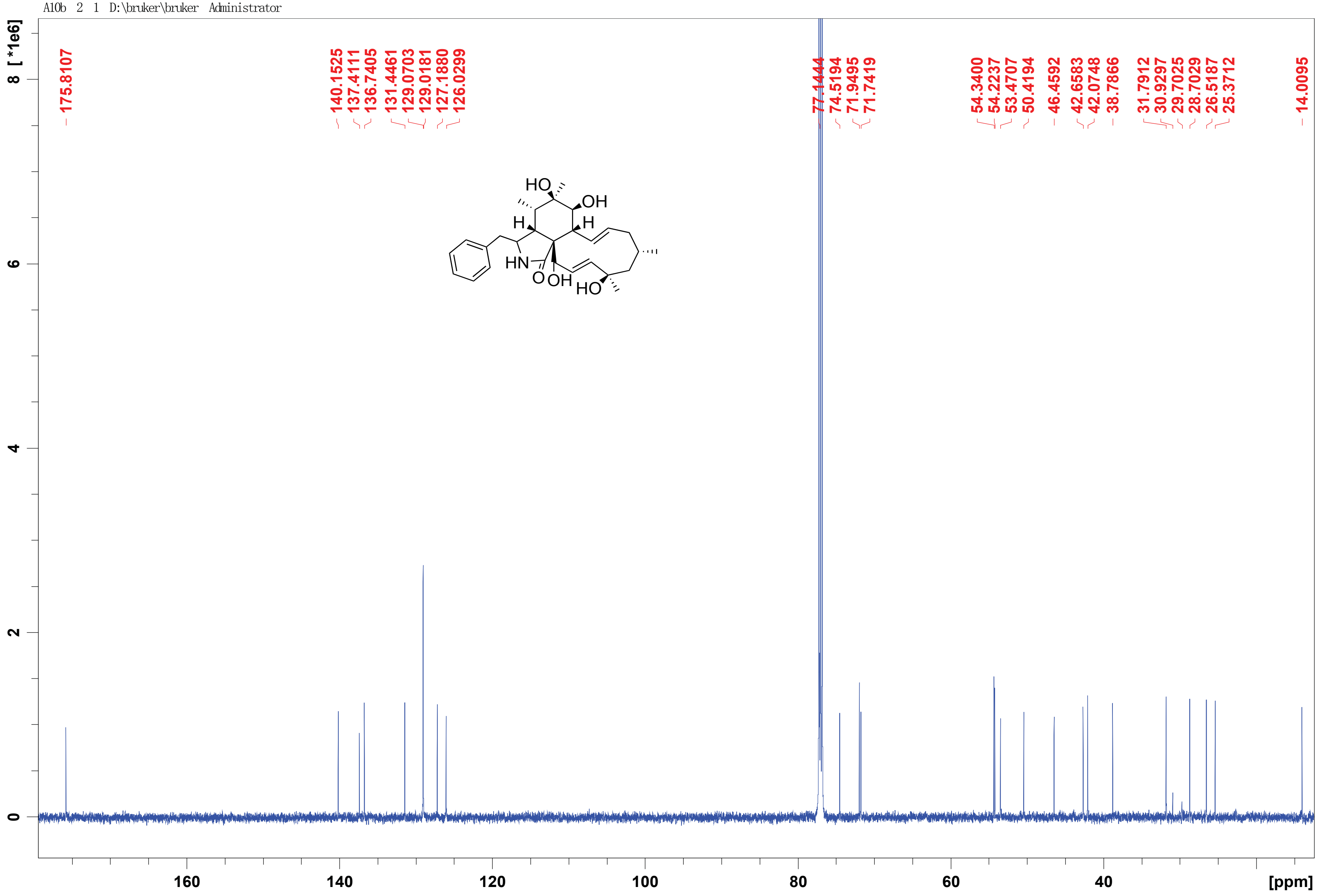

Figure S66. ^{13}C-NMR (150 MHz, $CDCl_3$) spectrum of cytochalasin R (**17**)

A15b 1 1 D:\bruker\bruker Administrator

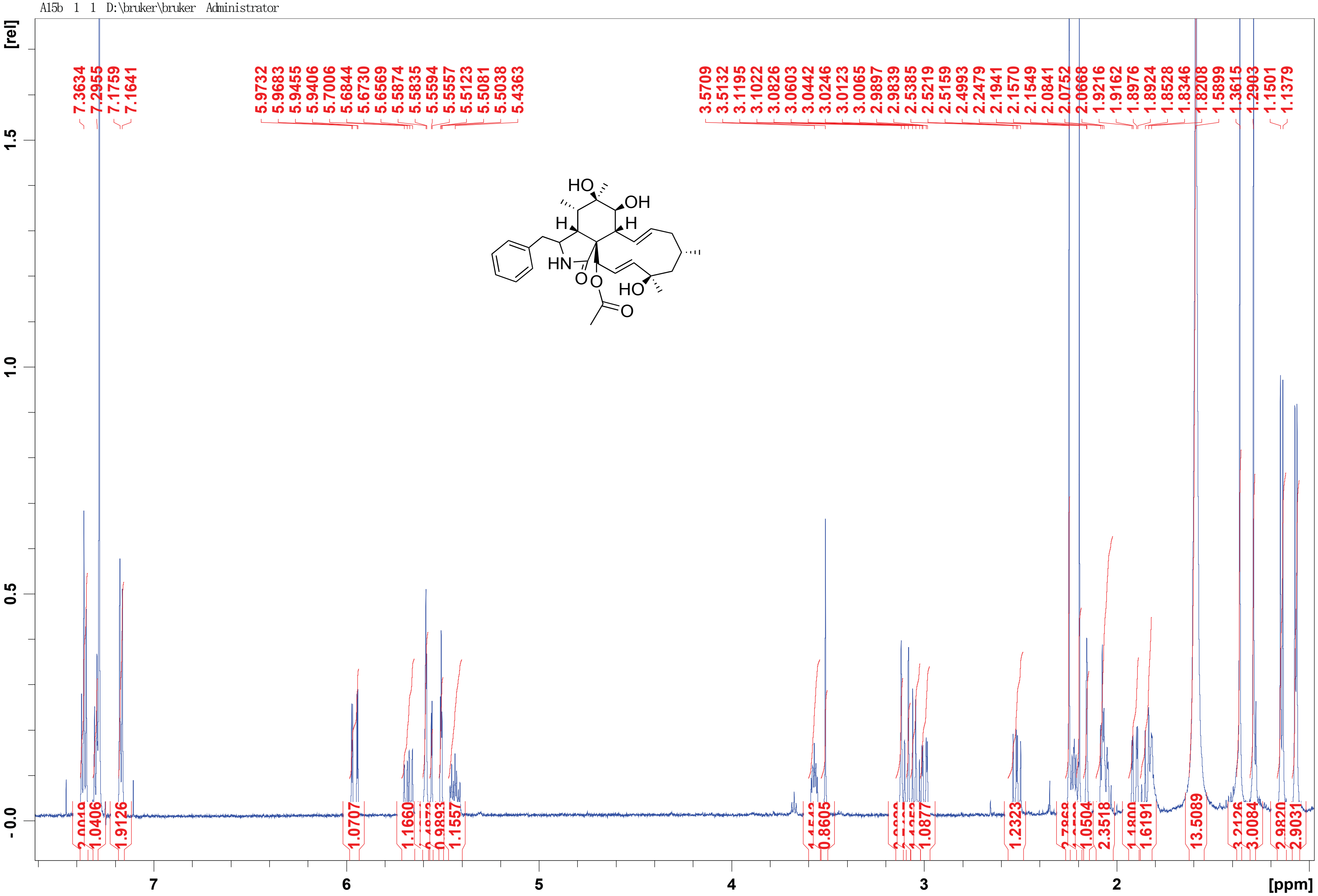

Figure S67. ^{1}H-NMR (600 MHz, $CDCl_3$) spectrum of cytochalasin R1 (**18**)

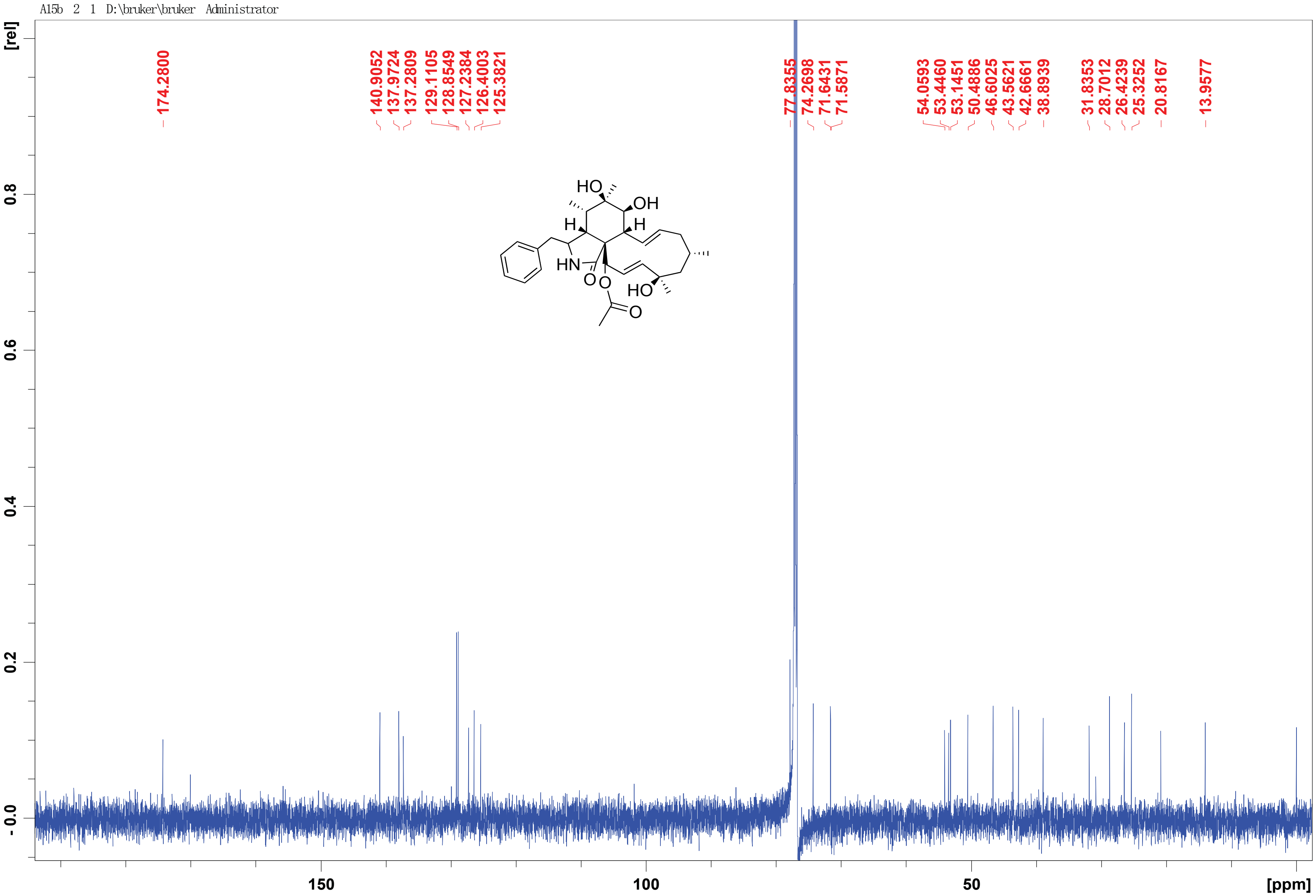

Figure S68. ^{13}C-NMR (150 MHz, $CDCl_3$) spectrum of cytochalasin R1 (**18**)

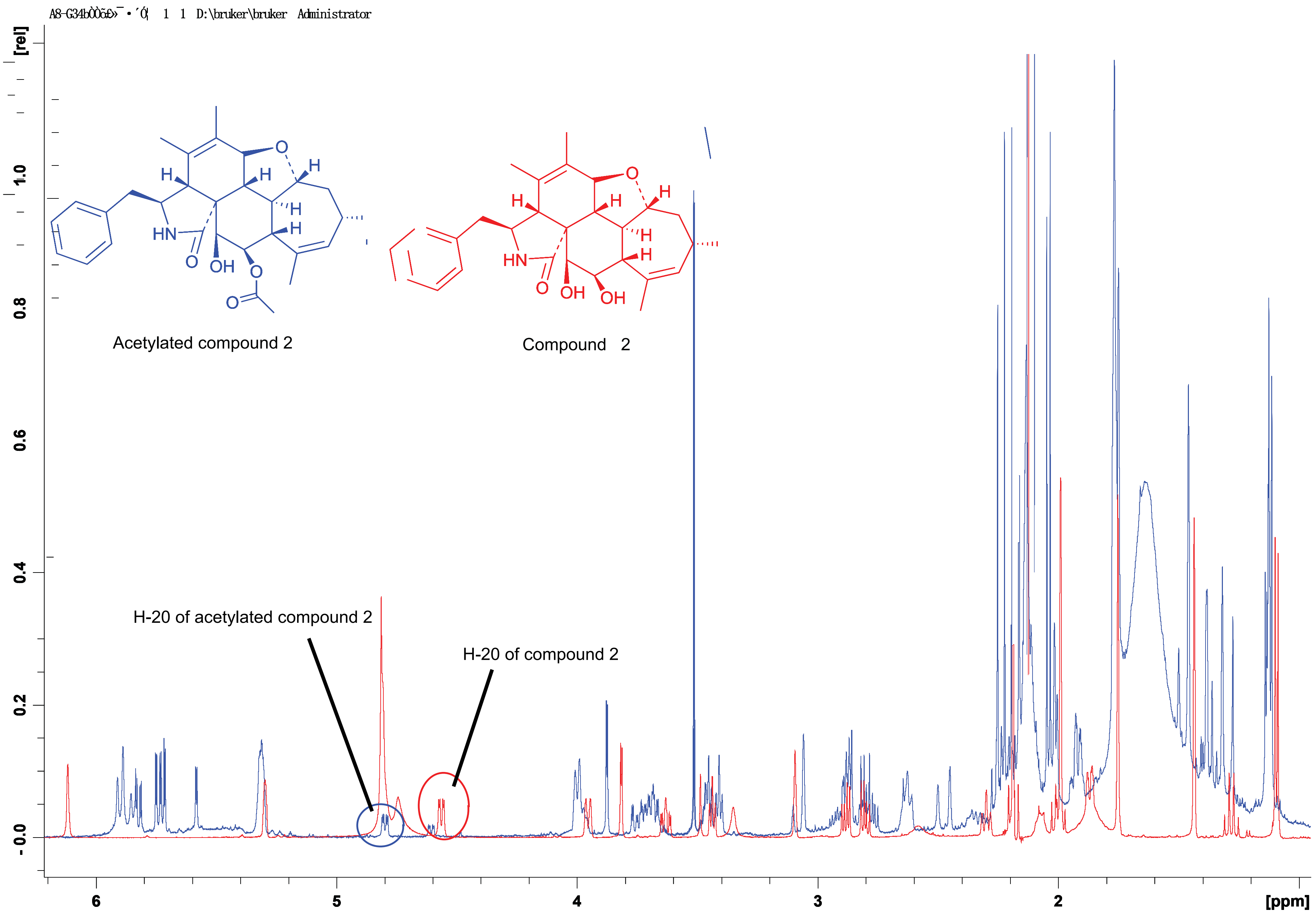

Figure S69. ^{1}H-NMR (600 MHz, $CDCl_3$) spectrum of acetylated phomopchalasin C_2 (**2A**)

National Center for Organic Mass Spectrometry in Shanghai
Shanghai Institute of Organic Chemistry
Chinese Academic of Sciences
High Resolution MS DATA REPORT

Instrument: Thermo Fisher Scientific LTQ FT Ultra

Card Serial Number : E150463

Sample Serial Number: A8-G33c

Operator : ZFJ Date: 2015/10/15

Operation Mode: DART-Positive

Elemental composition search on mass 434.27

m/z= 429.27-439.27

m/z	Theo. Mass	Delta (ppm)	RDB equiv.	Composition
434.2682	434.2676	1.36	12.0	$C_{26}H_{34}O_2N_4$
	434.2690	-1.73	11.5	$C_{28}H_{36}O_3N$
	434.2663	4.44	7.0	$C_{25}H_{38}O_6$

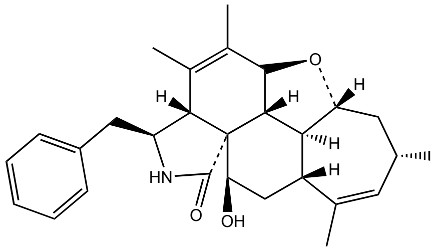

Chemical Formula: $C_{28}H_{35}NO_3$
Exact Mass: 433.26
Molecular Weight: 433.59

National Center for Organic Mass Spectrometry in Shanghai
Shanghai Institute of Organic Chemistry
Chinese Academic of Sciences
High Resolution MS DATA REPORT

Instrument: Thermo Fisher Scientific LTQ FT Ultra

Card Serial Number : E150465

Sample Serial Number: A8-G34b

Operator : ZFJ Date: 2015/10/15

Operation Mode: DART-Positive

Elemental composition search on mass 450.26

m/z= 445.26-455.26

m/z	Theo. Mass	Delta (ppm)	RDB equiv.	Composition
450.2629	450.2625	0.84	12.0	$C_{26}H_{34}O_3N_4$
	450.2639	-2.14	11.5	$C_{28}H_{36}O_4N$
	450.2612	3.81	7.0	$C_{25}H_{38}O_7$

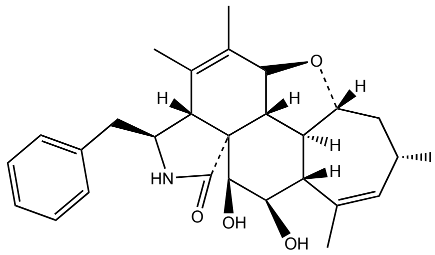

Chemical Formula: $C_{28}H_{35}NO_4$
Exact Mass: 449.26
Molecular Weight: 449.59

Shanghai Institute of Organic Chemistry
Chinese Academic of Sciences
High Resolution MS DATA REPORT

Instrument: Thermo Fisher Scientific LTQ FT Ultra

Card Serial Number : E151549

Sample Serial Number: A8-G31a

Operator :ZHUFJ Date: 2015/12/28

Operation Mode: DART Positive
Elemental composition search on mass 434.27

m/z= 429.27-439.27

m/z	Theo. Mass	Delta (ppm)	RDB equiv.	Composition
434.2689	434.2690	-0.16	11.5	$C_{28}H_{36}O_3N$

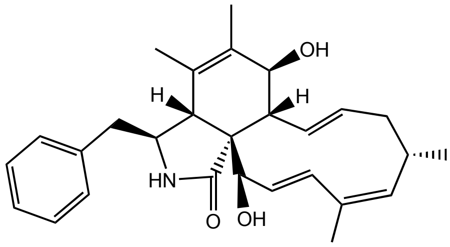

Chemical Formula: $C_{28}H_{35}NO_3$
Exact Mass: 433.26
Molecular Weight: 433.59

Shanghai Institute of Organic Chemistry
Chinese Academic of Sciences
High Resolution MS DATA REPORT

Instrument: Thermo Fisher Scientific LTQ FT Ultra

Card Serial Number : E151550

Sample Serial Number: A8-G31b

Operator :ZHUFJ Date: 2015/12/28

Operation Mode: DART Positive

Elemental composition search on mass 434.27

m/z= 429.27-439.27

m/z	Theo. Mass	Delta (ppm)	RDB equiv.	Composition
434.2686	434.2690	-0.78	11.5	$C_{28}H_{36}O_3N$

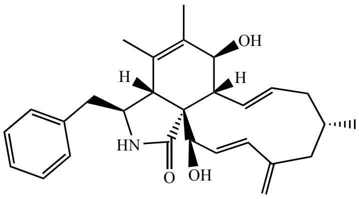

Chemical Formula: $C_{28}H_{35}NO_3$
Exact Mass: 433.26
Molecular Weight: 433.59

National Center for Organic Mass Spectrometry in Shanghai
Shanghai Institute of Organic Chemistry
Chinese Academic of Sciences
High Resolution MS DATA REPORT

Instrument: Thermo Fisher Scientific LTQ FT Ultra

Card Serial Number : E171220

Sample Serial Number: A10c

Operator :ZHUFJ Date: 2017/3/21

Operation Mode: DART Positive

Elemental composition search on mass 470.29

m/z= 465.29-475.29

m/z	Theo. Mass	Delta (ppm)	RDB equiv.	Composition
470.2897	470.2901	-0.89	9.5	$C_{28}H_{40}O_5N$
	470.2874	4.81	5.0	$C_{25}H_{42}O_8$

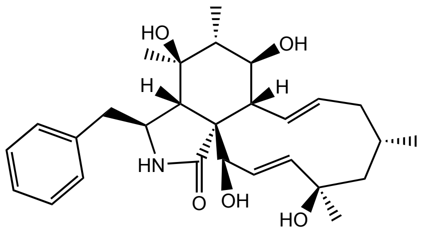

Chemical Formula: $C_{28}H_{39}NO_5$
Exact Mass: 469.28
Molecular Weight: 469.62

National Center for Organic Mass Spectrometry in Shanghai
Shanghai Institute of Organic Chemistry
Chinese Academic of Sciences
High Resolution MS DATA REPORT

Instrument: Thermo Fisher Scientific LTQ FT Ultra

Card Serial Number : E170257

Sample Serial Number: B7-A3b

Operator :zhufj Date: 2016/12/28

Operation Mode: DART Positive

Elemental composition search on mass 452.28

m/z= 447.28-457.28

m/z	Theo. Mass	Delta (ppm)	RDB equiv.	Composition
452.2799	452.2795	0.76	10.5	$C_{28}H_{38}O_4N$

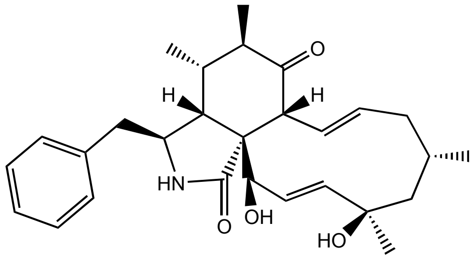

Chemical Formula: $C_{28}H_{37}NO_4$
Exact Mass: 451.27
Molecular Weight: 451.61

Shanghai Institute of Organic Chemistry
Chinese Academic of Sciences
High Resolution MS DATA REPORT

Instrument: Thermo Fisher Scientific LTQ FT Ultra

Card Serial Number : E151548

Sample Serial Number: A7b

Operator :ZHUFJ Date: 2015/12/28

Operation Mode: DART Positive

Elemental composition search on mass 512.30

m/z= 507.30-517.30

m/z	Theo. Mass	Delta (ppm)	RDB equiv.	Composition
512.3000	512.3007	-1.22	10.5	$C_{30}H_{42}O_6N$
	512.3012	-2.29	8.0	$C_{32}H_{49}Br$
	512.2980	4.01	6.0	$C_{27}H_{44}O_9$

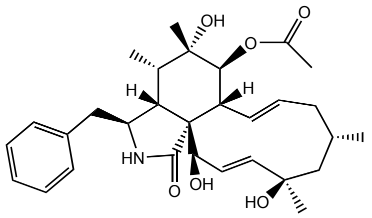

Chemical Formula: $C_{30}H_{41}NO_6$
Exact Mass: 511.29
Molecular Weight: 511.66

National Center for Organic Mass Spectrometry in Shanghai
Shanghai Institute of Organic Chemistry
Chinese Academic of Sciences
High Resolution MS DATA REPORT

Instrument: Thermo Fisher Scientific LTQ FT Ultra

Card Serial Number : E162572

Sample Serial Number: A8-F23b

Operator : ZHUFJ Date: 2016/08/31

Operation Mode: DART Positive

Elemental composition search on mass 466.30

m/z= 461.30-471.30

m/z	Theo. Mass	Delta (ppm)	RDB equiv.	Composition
466.2952	466.2952	0.07	10.5	$C_{29}H_{40}O_4N$
	466.2965	-2.79	15.5	$C_{30}H_{36}N_5$
	466.2938	2.95	11.0	$C_{27}H_{38}O_3N_4$

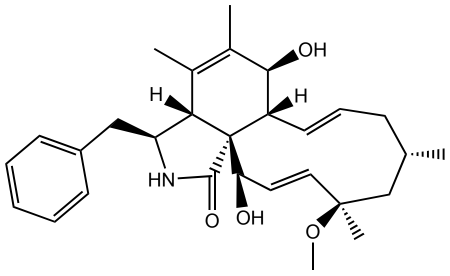

Chemical Formula: $C_{29}H_{39}NO_4$
Exact Mass: 465.29
Molecular Weight: 465.63

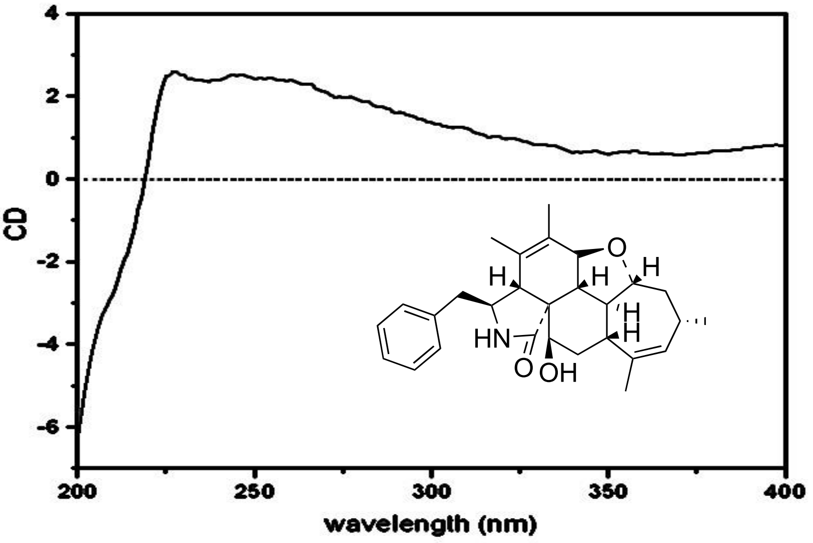

Figure S78 CD spectrum of phomopchalasin C_1 (**1**)

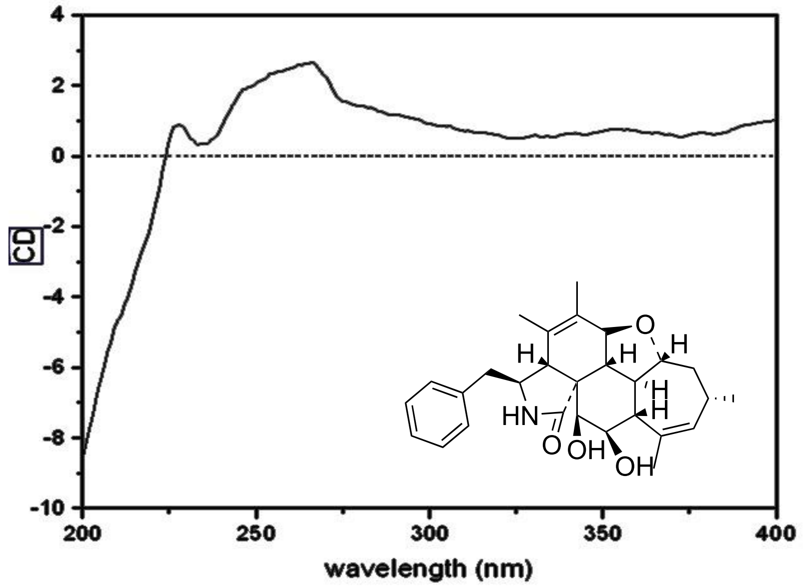

Figure S79 CD specturm of phomopchalasin C_2 (**2**)

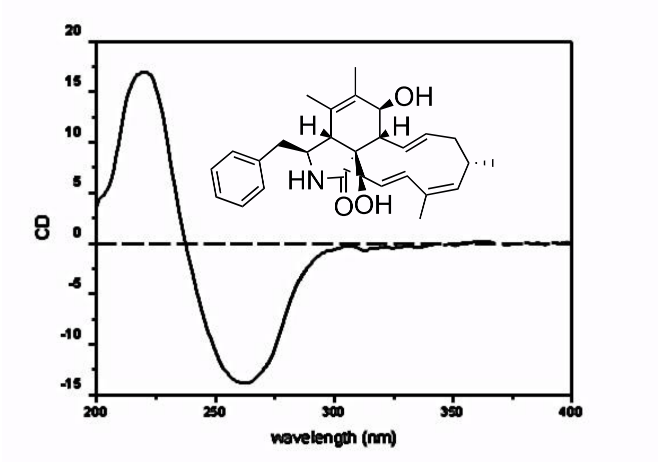

Figure S80 CD spectrum of phomopchalasin C_3 (**3**)

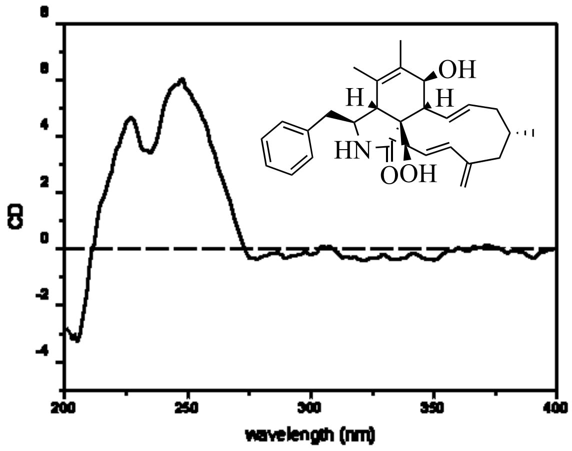

Figure S81 CD spectrum of phomopchalasin C_4 (**4**)

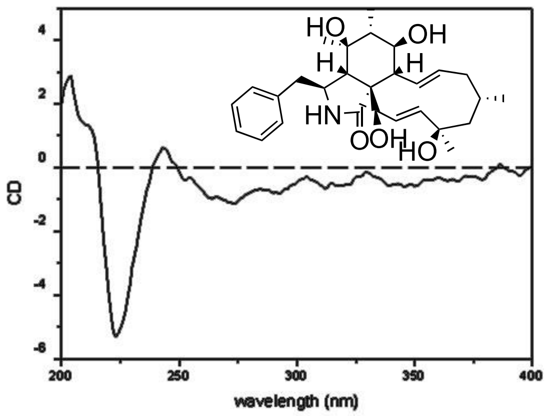

Figure S82   CD spectrum of phomopchalasin C_5 (**5**)

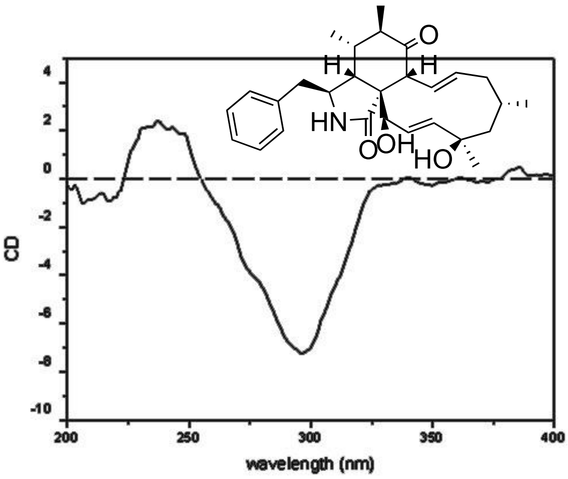

Figure S83   CD spectrum of phomopchalasin C_6 (**6**)

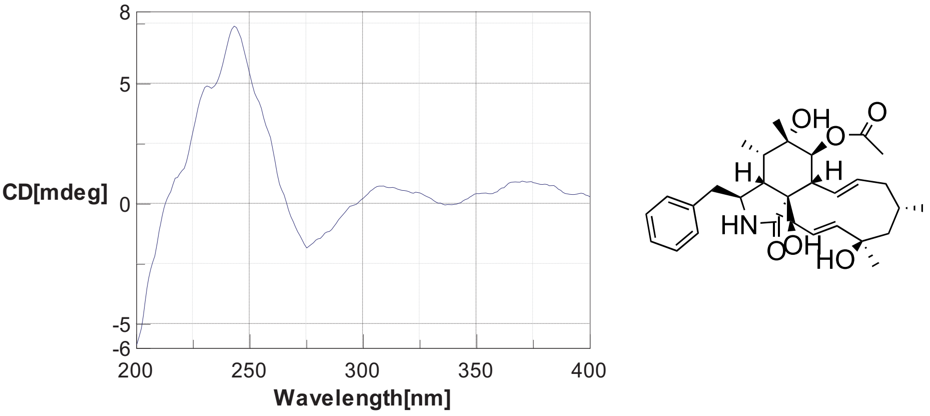

Figure S84 CD spectrum of phomopchalasin C_7 (**7**)

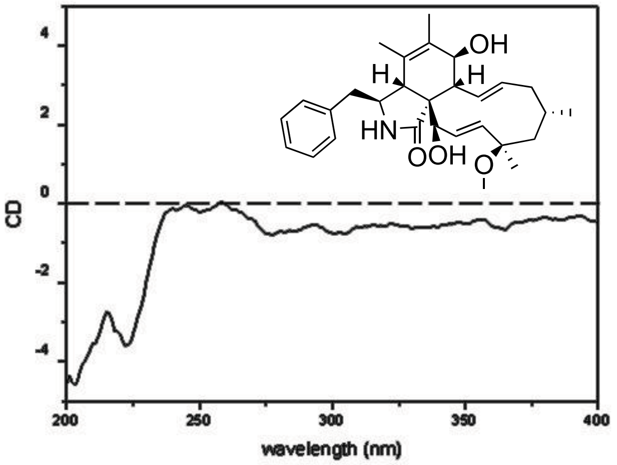

Figure S85 CD spectrum of phomopchalasin C_8 (**8**)

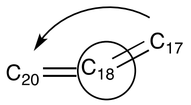

a

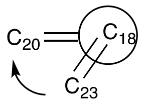

b

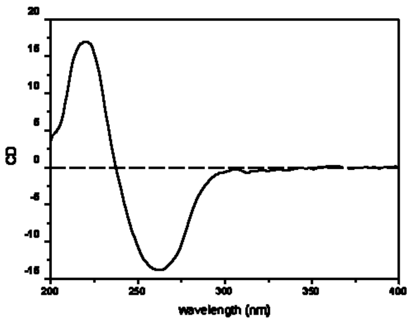

c

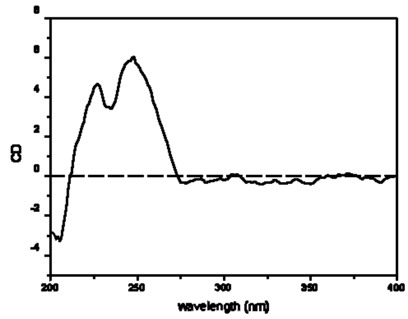

d

Figure S86. Cotton effect-spiral chirality correlation for compound **3** and **4**. (a) the left-handed spiral of conjugated diene of **3**; (c) negative cotton effect at 260 nm of **3**; (b) the right-handed spiral of conjugated diene of **4**; (d) positive cotton effect at 250 nm of **4**.

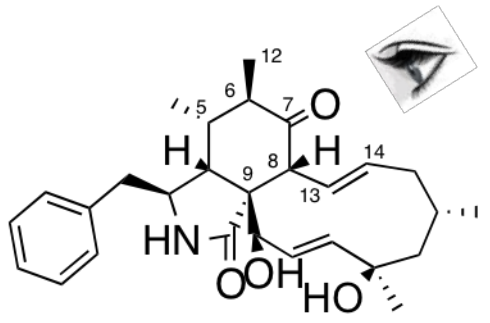

Figure S87. Illustration of the viewpoint of octant rule for compound **6**

Chart S1 Structure of compound **9-18**

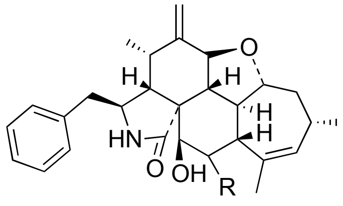

9 R = H cytochalasin J3
10 R = OH phomopchalasin B

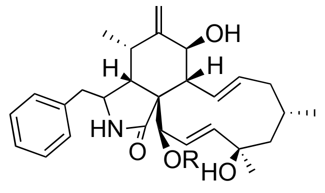

11 R = AC cytochalasin H
12 R = H cytochalasin J

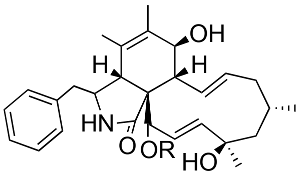

13 R = AC cytochalasin N
14 R = H cytochalasin O

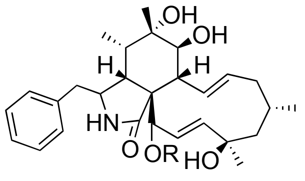

15 R = AC cytochalasin P
16 R = H cytochalasin Q

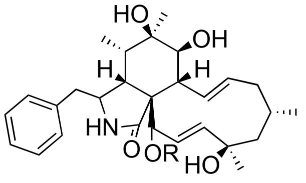

17 R = H cytochalasin R
18 R = AC cytochalasin R1
